# Supplementary material for: Depolymerization of robust polyetheretherketone to regenerate monomer units using sulfur reagents
Source: Commun Chem. 2023 Jan 24;6:14. doi: 10.1038/s42004-023-00814-8 (PMC9873933; doi:10.1038/s42004-023-00814-8)
Supplement: Supplementary file 2 — Supplementary information [file 42004_2023_814_MOESM2_ESM.pdf]

Supplementary Information

Depolymerization of robust polyetheretherketone to regenerate monomer units using sulfur reagents

Yasunori Minami,[1,2] Nao Matsuyama,[1] Yasuo Takeichi,[3] Ryota Watanabe,[4] Siby Mathew,[1] and Yumiko Nakajima[1]

[1] Interdisciplinary Research Center for Catalytic Chemistry (IRC3), National Institute of Advanced Industrial Science and Technology (AIST), Tsukuba Central 5, 1-1-1 Higashi, Tsukuba, Ibaraki 305-8565, Japan

[2] PRESTO, Japan Science and Technology Agency (JST), 1-1-1 Higashi, Tsukuba, Ibaraki 305-8565, Japan

[3] Department of Applied Physics, Graduate School of Engineering, Osaka University, 2-1 Yamadaoka, Suita, Osaka 565-0871, Japan

[4] Research Institute for Sustainable Chemistry, National Institute of Advanced Industrial Science and Technology (AIST), Tsukuba Central 5, 1-1-1 Higashi, Tsukuba, Ibaraki 305-8565, Japan

email: yasu-minami@aist.go.jp

Table of Contents

Supplementary Methods

General information……………………………………………………………………….....S3

Chemicals……………………………………………………………………………..…..S4

Supplementary Table 1 | Effect of Na_2S on the depolymerization of PEEK…………………….S5

Supplementary Table 2 | Effect of *n*-C_6H_{13}-SH/NaO*t*-Bu on the depolymerization of PEEK..…S6

Supplementary Table 3 | Effect of various thiols on the depolymerization of PEEK……..…….S7

Supplementary Table 4 | Effect of bases and solvents on the depolymerization of PEEK using $PhCH_2CH_2SH$…………………………………………………………………………...S8

Experimental procedures……...……………………………………………………….......S9

Spectrum data of products……….....………………………………………………….......S16

Experiments for the polymer synthesis……….......…………………………..………........S22

Additional experiments (**Supplementary Fig. 1-14**, **Supplementary Table 5**)…...….…………...S25

Relative free energies analyzed by DFT calculations (**Supplementary Fig. 15-17**).…………....S32

Supplementary references..…….……………………………………………….……..…S35

General information.

All manipulations of oxygen- and moisture-sensitive materials were conducted in a dry box under an argon atmosphere. Flash column chromatography was performed using Biotage Sfär Silica D - Duo 60 µm. Analytical TLC was performed on Merck Kieselgel 60 F254 (0.25 mm) plates. Visualization was accomplished with UV light (254 nm). HPLC was performed by JAI LC-9210NEXT. ^{1}H and $^{13}C\{^{1}H\}$ NMR spectra in $CDCl_3$ or acetone-d_6 solution were recorded with Bruker AVANCE III HD 600 spectrometer. The ^{1}H NMR (600 MHz) and $^{13}C\{^{1}H\}$ NMR (151 MHz) chemical shifts were reported in δ (ppm). ^{1}H NMR and $^{13}C\{^{1}H\}$ NMR spectra were referenced to the residual solvent signals or tetramethylsilane. ^{1}H NMR data are reported as follows: chemical shift, multiplicity (s = singlet, d = doublet, t = triplet, q = quartet, quint = quintet, sext = sextet, sept = septet, br = broad, m = multiplet), coupling constants (Hz), and integration. Melting points were measured by a MPA100 Optimelt Automated Melting Point System. High-resolution mass spectra (HRMS) were measured on a Bruker micrOTOF II mass spectrometer under positive electrospray ionization (ESI^{+}) conditions. The molecular weight of polymer products was determined by gel permeation chromatography (GPC) performed at 40 °C in chloroform. The GPC apparatus was JASCO 2000Plus series consisting of DG-2080-53, PU2080Plus, AS-2057Plus, CO-2065Plus, UV-2070Plus, RI-2031, two K-804L columns, and K-G4A guard column. Polystyrene standards (Shodex SM-105, SL-105) used for calibration in GPC were purchased from Shodex. A temperature-programmable micro-furnace pyrolyzer (PY-2020D, Frontier Lab, Japan) was directly coupled with a gas chromatography/mass spectrometry (GC/MS) system (QP2020, Shimadzu, Japan). A sample size of approximately 0.1 mg was used for the evolved gas analysis-mass spectroscopy (EGA-MS) measurements, a quantity small enough to achieve instant thermodynamic equilibrium during programmed heating. A given sample was placed in a deactivated stainless steel sample cup and heated in the pyrolyzer from 100 to 700 ˚C at a heating rate of 10 ˚C/min in a helium atmosphere. A proportion of the flow (1 mL/min), reduced by a GC splitter (50:1), was continuously introduced into the MS via a transfer capillary (UADTM-5M, 0.25 mm i.d.×5 m long, Frontier Lab, Japan). The transfer capillary was maintained at 300 ˚C in the GC oven to prevent condensation of less volatile products in the capillary. For the MS measurements, electro-ionization (EI) was carried out with an operating mass range of m/z 29 - 500 and a scan rate of 6 s/scan. The pyrolysis-gas chromatography/mass-spectrometry (Py-GC/MS) measurements were performed to identify and quantify the individually evolved products. In this case, the capillary transfer line for the EGA-MS system was replaced by a metal capillary separation column (Ultra Alloy+-5, 0.25 mm i.d. × 5 m long, Frontier Lab) coated with a 0.25-µm film of immobilized 5% diphenyl-95% dimethylpolysiloxane. The flash pyrolysis temperature was fixed at

600 ˚C throughout the entire data acquisition. After finishing each heating period, the column temperature was quickly raised to 40 ˚C (2 min hold) and then heated to 320 ˚C at the rate of 20 ˚C/min and held for 13 min. The other conditions were basically the same as those for the EGA-MS measurements described above. The S *K*-edge X-ray absorption near-edge structures (XANES) spectra were measured on the Photon Factory BL-9A beamline.

Chemicals.

All reactions were carried out under an argon atmosphere except of gram-scale reaction under reflux which was performed under nitrogen atmosphere. Unless otherwise noted, commercially available reagents were used without further purification. Powder, pellet, and film forms of polyetheretherketone (PEEK) (Powder: mean particle size 80micron, Cat. No. GF75065755. Pellet: average M_w ~20800, average M_n ~10300, Cat. No. 456640. Film: thickness 0.025 mm, Cat. No. GF55060231), bis(4-phenoxyphenyl)methanone (Cat. No. APO455830793), pellet form of polypropylene (isotactic, average M_w ~250,000, Cat. No. 182389), and pellet form of Nylon 6 (Cat. No. 181110) were purchased from Sigma–Aldrich Japan. Dehydrated 1,3-dimethyl-2-imidazolidinone (Cat. No. 11208-00) was purchased from the Kanto Chemicals. Dehydrated 1-methyl-2-pyrrolidone (Cat. No. 131-17615), *N*,*N*-dimethylacetamide (Cat. No. 042-32353), xylene (Cat. No. 240-00865), and pellet form of polystyrene (average of polymerization degree: 2,000. Cat. No. 198-12805) used in the study were purchased from the FUJIFILM Wako Chemicals. Carbon or glass fiber (30wt%)-enforced PEEK materials made from Ensinger (TECAPEEK CF30 or TECAPEEK GF30) was used after roughly ground, which were purchased from Monotaro (Cat. No. 3-3094-02 and 3-3095-01).

Supplementary Table 1 | Effect of Na_2S on the depolymerization of PEEK

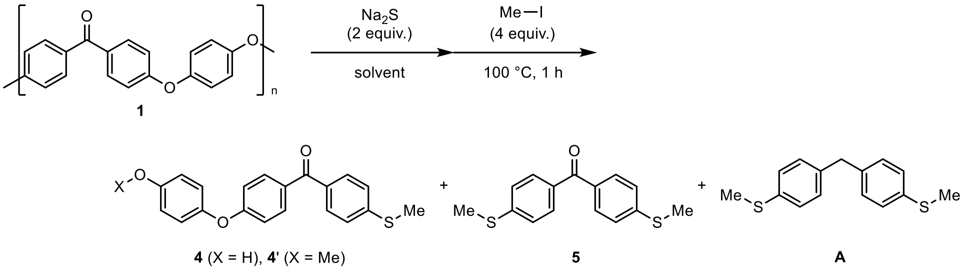

Entry	**1**	solvent (M)	temp (°C)	time (h)	**4** (%)	**4’** (%)	**5** (%)	**A** (%)
1	powder	NMP (0.5)	200	4	7	5	15	trace
2	powder	DMI (0.5)	150	17	44	ND	10	ND
3	powder	DMI (0.1)	150	19	12	trace	3	ND
4[a]	powder	DMI (0.1)	150	19	16	trace	2	ND
5	powder	DMI (0.5)	200	17	38	20	21	ND
6	pellet	DMI (0.5)	150	88	17	32	16	trace
7	pellet	DMI (0.5)	170	88	13	32	18	trace
8	pellet	DMI (0.5)	200	16	21	24	17	trace
9[b]	pellet	DMI (0.5)	200	17	22	ND	22	ND
10	pellet	NMP (0.5)	120	64	ND	ND	ND	ND
11	pellet	NMP (0.5)	150	88	33	25	20	trace
12	pellet	NMP (0.5)	170	88	18	55	19	trace
13	pellet	NMP (0.5)	200	17	61	ND	25	3
14[b,c]	pellet	NMP (0.05)	200	18	67	ND	25	trace

Conditions: A mixture of **1** (0.1 mmol relative to the molecular weight of the monomer, average M_w ~20800, average M_n ~10300), Na_2S, and NMP (relative to monomer of **1**) was stirred. Then, iodomethane (0.4 mmol) was added to this mixture and the resultant solution was stirred. Yields were determined by GC and NMR. [a]Tetrabutylammonium bromide as an additive (20 mol%). [b]Use of Na_2S (4 equiv.). [c]Use of K_2CO_3 as an additive (10 mol%).

Supplementary Table 2 | Effect of *n*-C_6H_{13}-SH/NaO*t*-Bu on the depolymerization of PEEK

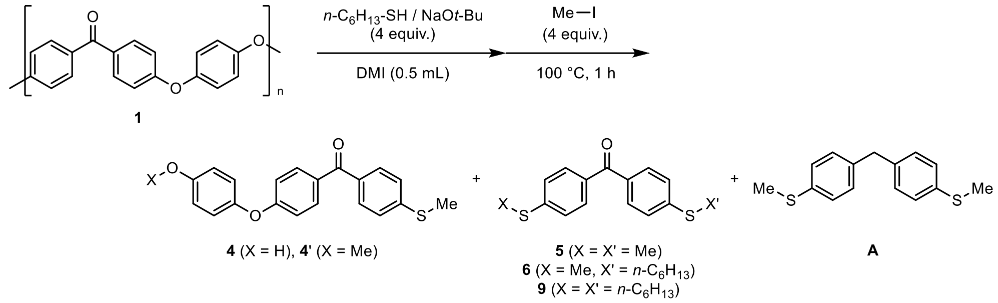

Entry	**1**	temp (°C)	time (h)	**4** (%)	**4’** (%)	**5** (%)	**6** (%)	**9** (%)	**A** (%)
1	powder	150	5	ND	4	26	67	3	ND
2	powder	150	17	trace	trace	51	38	1	ND
3	pellet	150	63	ND	ND	36	48	2	trace
4	pellet	170	15	ND	ND	35	43	3	3
5	pellet	170	64	ND	ND	74	19	2	1
6	pellet	200	5	ND	ND	61	12	2	1
7	pellet	200	90	ND	ND	35	1	trace	55
8	film	200	16	ND	ND	70	1	ND	29

Conditions: A mixture of **1** (0.1 mmol relative to the molecular weight of the monomer, average M_w ~20800, average M_n ~10300), *n*-hexanethiol (0.4 mmol), NaO*t*-Bu (0.4 mmol), and DMI (0.5 M relative to monomer of **1**) was stirred. Then, iodomethane (0.4 mmol) was added to this mixture and the resultant solution was stirred. Yields were determined by GC and NMR.

**Supplementary Table 3 | ** Effect of various thiols on the depolymerization of PEEK

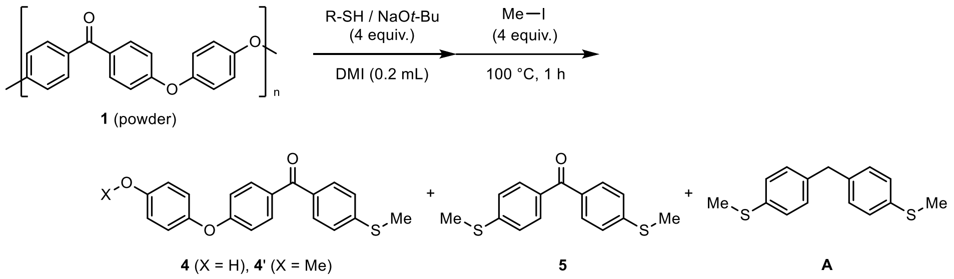

Entry	R	temp (°C)	time (h)	**4** (%)	**4’** (%)	**5** (%)	**A** (%)
1	$PhCH_2$	150	1	7	38	33	ND
2	$PhCH_2$	150	5	37	ND	56	ND
3	$PhCH_2$	150	22	41	ND	58	1
4	$PhCH_2$	150	64	15	ND	75	3
5	$PhCH_2$	150	208	4	9	83	2
6[a]	$PhCH_2$	100	20	ND	46	35	ND
7[a]	$PhCH_2$	100	64	ND	37	43	ND
8	$PhCH_2$	160	24	35	ND	51	4
9	$PhCH_2$	170	64	trace	ND	76	11
10[b]	$PhCH_2$	150	1	ND	52	22	3
11[c]	$PhCH_2$	150	1	39	20	38	ND
12	*p*-TolylCH_2	150	25	36	11	53	ND
13	*o*-TolylCH_2	150	16	ND	35	65	ND
14	PhCH(Me)	150	16	52	ND	37	ND
15	$PhCH_2CH_2$	150	24	trace	6	88	ND
16	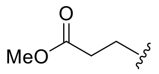	150	15	2	ND	ND	ND
17	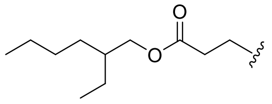	150	15	ND	9	trace	ND
18[d]	$HSCH_2CH_2$	150	22	17	ND	28	trace
19	$HOCH_2CH_2$	150	18	ND	6	7	ND

Conditions: A mixture of **1** (0.1 mmol relative to the molecular weight of the monomer, average M_w ~20800, average M_n ~10300), thiol (0.4 mmol), NaO*t*-Bu (0.4 mmol), and DMI (0.5 M relative to monomer of **1**) was stirred. Then, iodomethane (0.4 mmol) was added to this mixture and the resultant solution was stirred. Yields were determined by GC and NMR. [a]After adding iodomethane, the mixture was stirred for 3 h. [b]Use of LiO*t*-Bu instead of NaO*t*-Bu. [c]Use of KO*t*-Bu instead of NaO*t*-Bu. [d]2 equiv. of $HSCH_2CH_2SH$ and NaO*t*-Bu was used.

Supplementary Table 4 | Effect of bases and solvents on the depolymerization of PEEK using $PhCH_2CH_2SH$

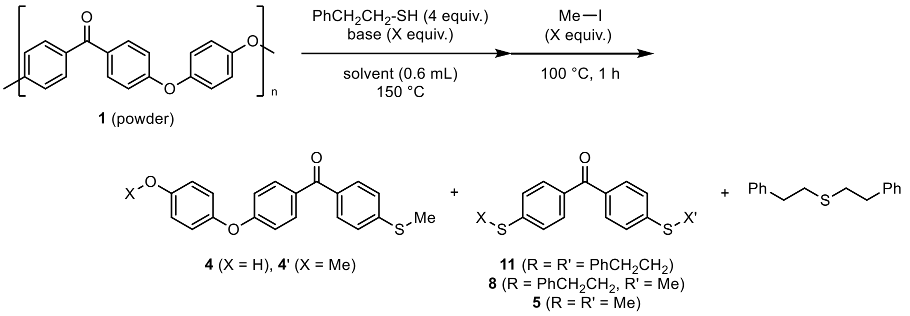

Entry	base (equiv)	solvent	time (h)	**4** (%)	**4'** (%)	**11** (%)	**8** (%)	**5** (%)	Sulfide (%)
1	NaO*t*-Bu (4)	DMI	24	trace	6	ND	ND	88	63
2[a]	NaO*t*-Bu (4)	DMI	25	3	6	ND	ND	86	76
3	NaO*t*-Bu (3)	DMI	1	3	7	trace	(60)	25	49
4	NaO*t*-Bu (3)	DMI	3	ND	3	trace	42	55	68
5	NaO*t*-Bu (3)	DMI	5	3	trace	trace	34	61	73
6	NaO*t*-Bu (3)	DMI	6	trace	2	trace	32	64	74
7	NaO*t*-Bu (3)	DMI	20	ND	trace	trace	trace	93 (84)	88
8	NaO*t*-Bu (2)	DMI	24	5	ND	ND	ND	73	54
9	K_3PO_4 (3)	DMI	20	ND	ND	ND	9	90	16
10	Et_3N (3)	DMI	20	ND	trace	ND	ND	ND	8
11	NaOH (3)	DMI	20	ND	ND	ND	ND	95	80
12	NaO*t*-Bu (3)	Xylene	20	ND	ND	ND	ND	ND	trace
13[b]	NaO*t*-Bu (3)	DMAc	20	ND	ND	ND	4	96 (93)	94
14	NaO*t*-Bu (3)	PhCN	20	ND	ND	44	6	ND	0.8
15[c]	NaO*t*-Bu (3)	DMAc	20	ND	ND	ND	ND	ND	ND

Conditions: A mixture of **1** (0.3 mmol relative to the molecular weight of the monomer, average M_w ~20800, average M_n ~10300), $PhCH_2CH_2SH$ (1.2 mmol), base (X equiv.), and solvent (0.6 mL) was stirred at 150 ºC. Then, iodomethane (X equiv.) was added to this mixture and the resultant solution was stirred at 100 ºC for 1 h. Yields were determined by GC and NMR. Numbers in parenthesis are isolated yields. [a]The reaction was examined under air atmosphere. [b]Styrene was observed in 4% yield. [c]30 ºC at first step.

Experimental procedures.

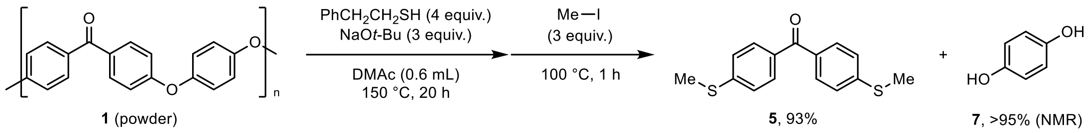

Depolymerization of PEEK powder by 2-phenylethanethiol and sodium *tert*-butoxide. *A general procedure for the depolymerization of PEEK. N,N*-Dimethylacetamide (0.60 mL) and 2-phenylethanethiol (167 mg, 1.21 mmol) were added to a mixture of PEEK powder (86.4 mg, 0.300 mmol relative to the molecular weight of the monomer) and sodium *tert*-butoxide (86.5 mg, 0.900 mmol) in a 3 mL vial in an argon atmosphere. The vial was closed with a screw cap, and the mixture was stirred at 150 °C for 20 h. After the liquid mixture cooled to room temperature, iodomethane (128 mg, 0.900 mmol) was added and stirred at 100 °C for 1 h. After ethyl acetate (1.5 mL) was added, the mixture was washed with aqueous HCl (2 M, 1 mL), water, and brine. At this time, the mixture was analyzed by ^{1}H NMR to determine the yields of hydroquinone (**7**) (>95%) and styrene (4%). The extracted organic layer was dried over $MgSO_4$ and concentrated *in vacuo*. The crude product was purified by column chromatography on silica gel (hexane/ethyl acetate, 96:4 to 7:3) to afford bis(4-(methylthio)phenyl)methanone (**5**) (93%, 75.9 mg).

The depolymerizations of PEEK followed by various functionalization on sulfer were examined according to the above procedure.

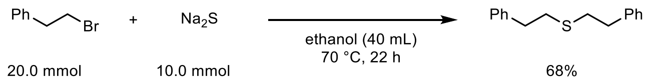

Preparation of di(phenylethyl)sulfide. Ethanol (40 mL) and 2-phenylbromoethane (3.70 g, 20.0 mmol) was added to sodium sulfide (781 mg, 10.0 mmol) in a 80 mL Schlenk flask at room temperature under nitrogen atmosphere. After stirring for 22 h at 70 ºC, the reaction mixture was cooled down to room temperature followed by filtered through Celite. After concentration in vacuo, the residue was purified by flash chromatography on silica gel using hexane as an eluent to give desired chemical in 68% yield (1.65 g, 6.82 mmol).

Di(phenylethyl)sulfide. CAS registry number: 27846-24-8.

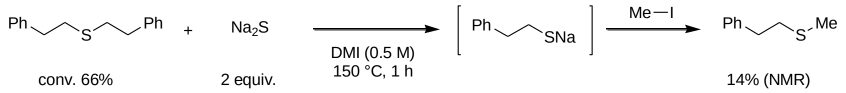

Reaction of di(phenylethyl)sulfide with sodium sulfide. To a mixture of sodium sulfide (15.6 mg, 0.200 mmol) and 1,3-dimethyl-2-imidazolidinone (0.2 mL) was added di(phenylethyl)sulfide (24.3

mg, 0.100 mmol) in a 3 mL vial under argon atmosphere. The vial was closed with a screw cap and stirred at 150 °C for 1 h. After the reaction mixture was cooled down to room temperature, iodomethane (57.0 mg, 0.402 mmol) was added to this mixture and stirred at 100 °C for 1 h. After ethyl acetate (1.5 mL) was added, the reaction mixture was washed with aq. HCl (2 M, 1 mL), water, and brine. The extracted organic layer was dried over $MgSO_4$ and concentrated in vacuo. The crude product was analyzed by ^1H NMR to determine the conversion of di(phenylethyl)sulfide (conv. 66%) and the yield of the products, methyl phenethyl sulfide (14%).

[2-(Methylthio)ethyl]benzene. CAS registry number: 5925-63-3.

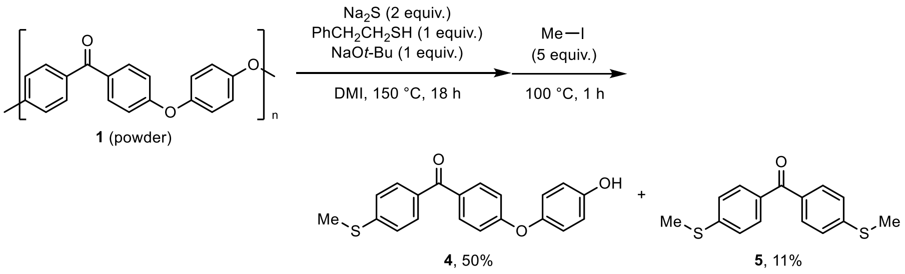

Depolymerization of PEEK powder by sodium sulfide, 2-phenylethanethiol, and sodium *tert*-butoxide. *For isolation of comonomer 4.* To a mixture of PEEK powder (28.9 mg, 0.10 mmol relative to the molecular weight of the monomer), sodium sulfide (15.7 mg, 0.201 mmol), and sodium *tert*-butoxide (9.8 mg, 0.102 mmol) was added 1,3-dimethyl-2-Imidazolidinone (0.20 mL) and 2-phenylethanethiol (14.4 mg, 0.104 mmol) in a 3 mL vial under argon atmosphere. The vial was closed with a screw cap and stirred at 150 °C for 18 h. After the reaction mixture was cooled down to room temperature, iodomethane (73.0 mg, 0.514 mmol) was added and stirred at 100 °C for 1 h. After ethyl acetate (1 mL) was added, the reaction mixture was washed with aq. HCl (2 M, 0.5 mL), water, and brine. The extracted organic layer was dried over $MgSO_4$ and concentrated in vacuo. The crude product was purified by column chromatography on silica-gel (hexane/ethyl acetate 9:1 to 7:3) to give (4-(4-hydroxyphenoxy)phenyl)(4-(methylthio)phenyl)methanone (**4**) (50%, 15.1 mg).

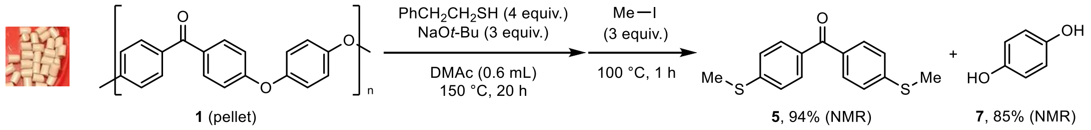

Depolymerization of PEEK pellets by 2-phenylethanethiol and sodium *tert*-butoxide. To a mixture of PEEK pellets (86.7 mg, 0.302 mmol relative to the molecular weight of the monomer), and sodium *tert*-butoxide (86.5 mg, 0.900 mmol) was added *N*,*N*-dimethylacetamide (0.60 mL) and 2-phenylethanethiol (167 mg, 1.21 mmol) in a 3 mL vial under argon atmosphere. The vial was closed with a screw cap and stirred at 150 °C for 20 h. After the liquid reaction mixture was cooled

down to room temperature, iodomethane (128 mg, 0.900 mmol) was added and stirred at 100 °C for 1 h. This mixture was analyzed by ^{1}H NMR to determine the yields of the products, **5** and hydroquinone (**7**) (94% and 85%), respectively, by using acetone-d_6 and 1,4-dioxane as an internal standard.

PEEK films was depolymerized and analyzed according to the same procedure.

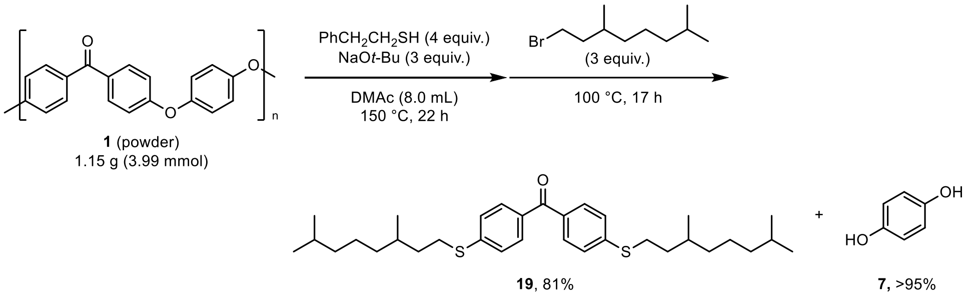

Gram scale depolymerization of PEEK (1). To a mixture of PEEK powder (1.15 g, 3.99 mmol relative to the molecular weight of the monomer), and sodium *tert*-butoxide (1.15 g, 12.0 mmol) was added *N*,*N*-dimethylacetamide (8.0 mL) and 2-phenylethanethiol (2.21 g, 16.0 mmol) in a 20 mL vial under argon atmosphere. The vial was closed with a screw cap and stirred at 150 °C for 22 h. After the liquid reaction mixture was cooled down to room temperature, 1-bromo-3,7-dimethyloctane (2.65 g, 12.0 mmol) was added and stirred at 100 °C for 17 h. The liquid reaction mixture was analyzed by ^{1}H NMR using acetone-d_6 to determine yields of products. After ethyl acetate (20 mL) was added, the reaction mixture was washed with aq. HCl (2 M, 10 mL), water, and brine. The extracted organic layer was dried over $MgSO_4$ and concentrated in vacuo. The crude product was purified by column chromatography on silica-gel (hexane/ethyl acetate 96:4 to 7:3) followed by removal of all volatiles under reduced pressure (ca. 0.5 Torr) at 170 °C to give bis(4-(3,7-dimethyl-*n*-octylthio)phenyl)methanone (**19**) (81%, 1.70 g).

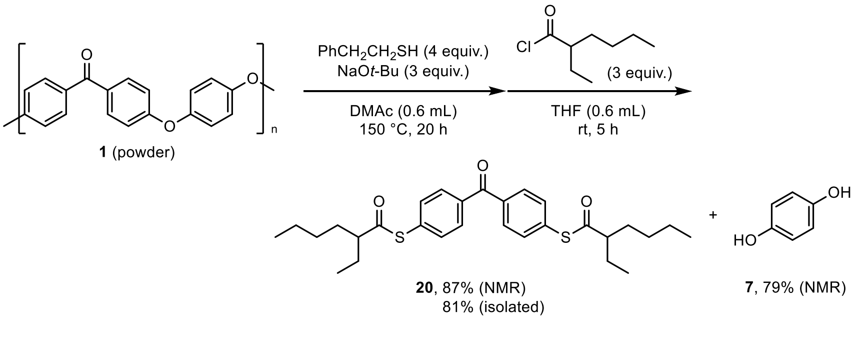

Depolymerization of PEEK (1) as a powder followed by the treatment with acid chloride. To a mixture of PEEK powder (86.1 mg, 0.299 mmol relative to the molecular weight of the monomer), and sodium *tert*-butoxide (87.0 mg, 0.905 mmol) was added *N*,*N*-dimethylacetamide (0.60 mL) and

2-phenylethanethiol (167 mg, 1.21 mmol) in a 3 mL vial under argon atmosphere. The vial was closed with a screw cap and stirred at 150 °C for 20 h. After the liquid reaction mixture was cooled down to room temperature, tetrahydrofuran (0.6 mL) and 2-ethylhexanoyl chloride (147 mg, 0.908 mmol) was added and stirred at room temperature for 5 h. The progress of the reaction was analyzed by ^{1}H NMR using acetone-d_6 and 1,4-dioxane as an internal standard to determine the yield of **20** and hydroquinone (**7**). After ethyl acetate (1.5 mL) was added, the reaction mixture was washed with aq. HCl (2 M, 1 mL), water, and brine. The extracted organic layer was dried over $MgSO_4$ and concentrated in vacuo. The crude product was purified by column chromatography on silica-gel (hexane/ethyl acetate 100:0 to 7:3) followed by the treatment of HPLC to give bis(4-(1-ethyl-*n*-penthylcarbonylthio)phenyl)methanone (**20**) (81%, 121 mg).

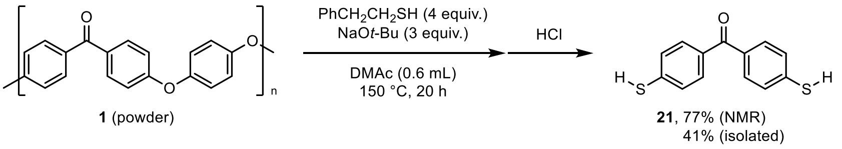

Depolymerization of PEEK (1) as a powder followed by the treatment with hydrogen chloride. To a mixture of PEEK powder (86.4 mg, 0.300 mmol relative to the molecular weight of the monomer), and sodium *tert*-butoxide (86.6 mg, 0.901 mmol) was added *N*,*N*-dimethylacetamide (0.60 mL) and 2-phenylethanethiol (167 mg, 1.21 mmol) in a 3 mL vial under argon atmosphere. The vial was closed with a screw cap and stirred at 150 °C for 20 h. Then, aq. HCl (2 M, 2.0 mL) was added and stirred for 1 h at room temperature. After dichloromethane was added, organic layer was extracted, dried over $MgSO_4$, and concentrated in vacuo. This crude product was analyzed by ^{1}H NMR to determine the yield of 4,4'-di(mercapto)benzophenone (**21**) (77%). The crude product was purified by on silica-gel (hexane/ethyl acetate 96:4 to 7:3) followed by recrystallization from dichloromethane/hexane to give **21** in 41% yield (31.8 mg).

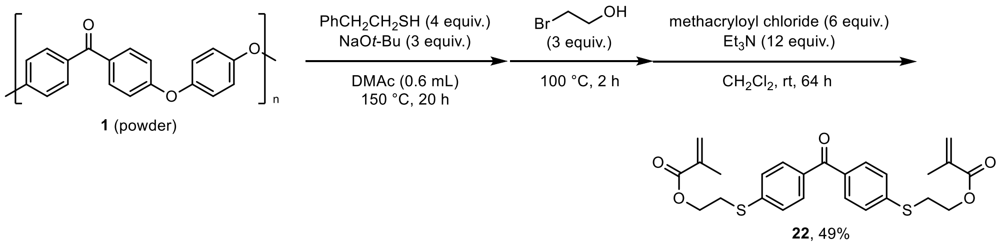

Three step, one-pot sequential depolymerization/alkylation/esterification. To a mixture of PEEK powder (86.4 mg, 0.300 mmol relative to the molecular weight of the monomer), and sodium *tert*-butoxide (86.5 mg, 0.900 mmol) was added *N*,*N*-dimethylacetamide (0.60 mL) and

2-phenylethanethiol (167 mg, 1.21 mmol) in a 3 mL vial under argon atmosphere. The vial was closed with a screw cap and stirred at 150 °C for 20 h. After the liquid reaction mixture was cooled down to room temperature, ethylene bromohydrin (128 mg, 0.910 mmol) was added to this mixture and stirred at 100 °C for 2 h. After the mixture was cooled down to room temperature, 4,4-dimethylaminopyridine (2.3 mg), dichloromethane (0.9 mL), triethylamine (365 mg, 3.61 mmol), and methacryloyl chloride (189 mg, 1.81 mmol) were added and stirred at room temperature for 64 h. After ethyl acetate (1.5 mL) was added, the reaction mixture was washed with aq. HCl (2 M, 1 mL), water, and brine. The extracted organic layer was dried over $MgSO_4$ and concentrated in vacuo. The crude product was purified by column chromatography on silica-gel (hexane/ethyl acetate 96:4 to 7:3) to give [{carbonylbis(4,1-phenylene)}bis(sulfanediyl)]bis(ethane-2,1-diyl) bis(2-methylacrylate) (**22**) (49%, 69.6 mg).

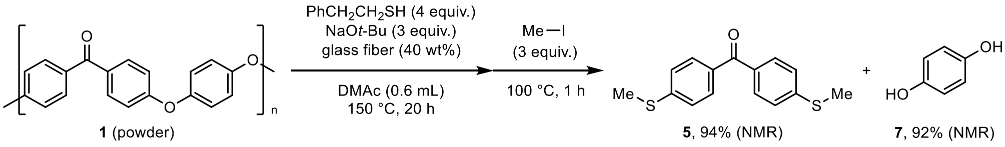

Depolymerization of PEEK (1) in the presence of glass fibers under the optimized conditions. To a mixture of PEEK powder (87.2 mg, 0.302 mmol relative to the molecular weight of the monomer), glass fiber (34.2 mg, 40 wt%), and sodium *tert*-butoxide (86.4 mg, 0.899 mmol) was added *N*,*N*-dimethylacetamide (0.60 mL) and 2-phenylethanethiol (167 mg, 1.21 mmol) in a 3 mL vial under argon atmosphere. The vial was closed with a screw cap and stirred at 150 °C for 20 h. After the liquid reaction mixture was cooled down to room temperature, iodomethane (128 mg, 0.900 mmol) was added to this mixture and stirred at 100 °C for 1 h. This mixture was analyzed by ^{1}H NMR to determine the yields of the products, **5** and hydroquinone (**7**) in 94% and 92% yields, respectively, by using acetone-d_6 and 1,4-dioxane as an internal standard.

PEEK pellets in the presence of other polymers were depolymerized and analyzed according to the same procedure.

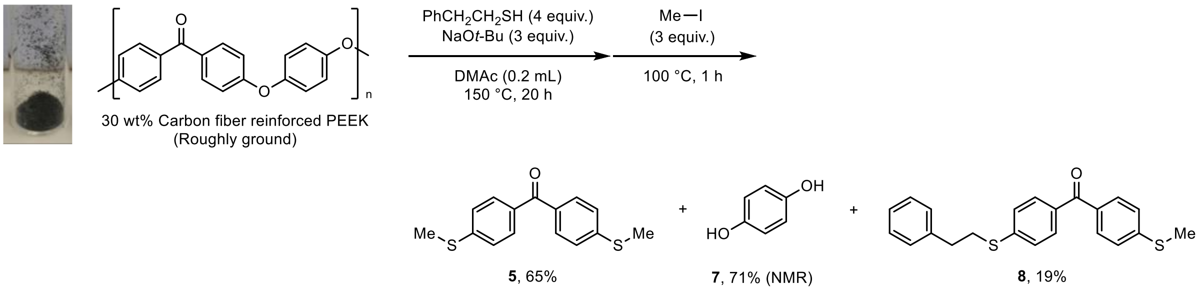

Depolymerization of 30 wt% carbon fiber reinforced PEEK (roughly ground) by 2-phenylethanethiol and sodium *tert*-butoxide. *N*,*N*-Dimethylacetamide (0.20 mL) and 2-phenylethanethiol (55.2 mg, 0.400 mmol) were added to a mixture of roughly ground 30 wt% Carbon fiber-reinforced PEEK (41.8 mg, 0.101 mmol relative to the molecular weight of the monomer) and sodium *tert*-butoxide (29.0 mg, 0.302 mmol) in a 3 mL vial in an argon atmosphere. The vial was closed with a screw cap, and the mixture was stirred at 150 °C for 20 h. After the liquid mixture cooled to room temperature, iodomethane (43.3 mg, 0.305 mmol) was added and stirred at 100 °C for 1 h. After ethyl acetate (1.0 mL) was added, the mixture was washed with aqueous HCl (2 M, 0.5 mL), water, and brine. At this time, the mixture was analyzed by ^{1}H NMR to determine the yields of hydroquinone (**7**) (71%) and styrene (7%). The extracted organic layer was dried over $MgSO_4$ and concentrated *in vacuo*. The crude product was purified by column chromatography on silica gel (hexane/ethyl acetate, 95:5 to 55:45) to afford bis(4-(methylthio)phenyl)methanone (**5**) (65%, 18.0 mg), 4-methylthiophenyl-4'-phenethylthiophenyl-methanone (**8**) (19%, 6.9 mg), and di(2-phenylethyl)sulfide (61%, 29.5 mg).

The same depolymerization was examined using 30 wt% glass fiber reinforced PEEK (roughly ground) (Table 2, Entry 6) and **5**, **8**, and di(2-phenylethyl)sulfide were isolated in 53%, 20%, and 62% yields, respectively.

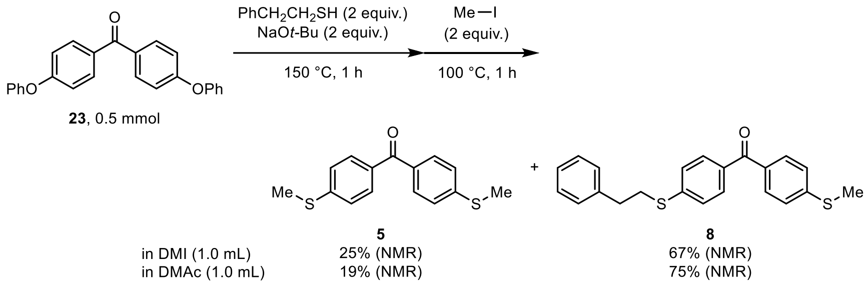

Reaction of 4,4'-diphenoxy-benzophenone (23) with 2-phenylethanethiol and sodium *tert*-butoxide. To a mixture of **23** (186 mg, 0.509 mmol), and sodium *tert*-butoxide (97.4 mg, 1.01 mmol) was added 1,3-dimethyl-2-imidazolidinone (1.0 mL) and 2-phenylethanethiol (139 mg, 1.01 mmol) in a 3 mL vial under argon atmosphere. The vial was closed with a screw cap and stirred at 150 °C for 1 h. Then, iodomethane (144 mg, 1.01 mmol) was added to this mixture and stirred at 100 °C for 1 h. After HCl in diethyl ether (1.0 M, 1 mL) was added, the reaction mixture was concentrated in vacuo. The crude product was analyzed by ^{1}H NMR to determine the yields of products: **5** (26%), 4-methylthio-4'-phenethylthiobenzophenone (**8**) (67%), styrene (37%), phenol (70%), and anisole (25%), respectively.

The same reaction was examined in *N,N,*-dimethylformamide (1.0 mL) and produced **5** (19%), **8** (75%), styrene (24%), phenol (64%), and anisole (18%), respectively.

Experiments in article Table 3, Entries 1 and 2 were performed according to above procedure.

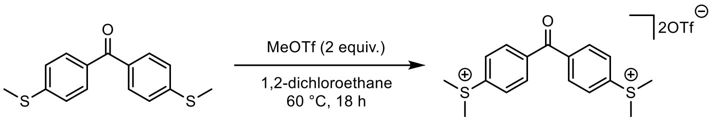

5

24, 97%

Reaction of bis(4-(methylthio)phenyl)methanone (5) with methyl trifluoromthanesulfonate.[1] To a mixture of **5** (1.00 g, 3.64 mmol) in 1,2-dichloroethane (3.65 mL) was added methyl trifluoromethanesulfonate (1.17 g, 7.28 mmol) in a 15 mL vial under argon atmosphere. The vial was closed with a screw cap and stirred at 60 °C for 18 h. After volatiles were removed under reduced pressure, the crude product was dissolved into acetone (4.2 mL) followed by precipitation into dichloroethane (42 mL) to give (carbonylbis(4,1-phenylene))bis(dimethylsulfonium) bistriflate (**24**) in 97% (2.14 g, 3.60 mmol) yield.

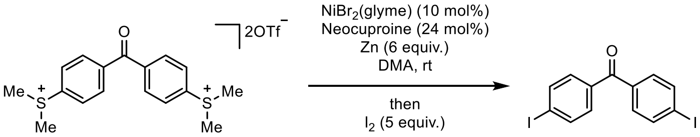

24

25, 25%

Iodination of (carbonylbis(4,1-phenylene))bis(dimethylsulfonium) bistriflate (24).[2] To a mixture of **24** (181 mg, 0.300 mmol), diglyme nickel dibromide (10.7 mg, 0.030 mmol), and neocuproine (2,9-dimethyl-1,10-phenanthroline) (15.1 mg, 0.073 mmol) was added zinc powder (118 mg, 1.80 mmol) and *N,N*-dimethylformamide (1.5 mL) in a 3 mL vial under argon atmosphere. The vial was closed with a screw cap and stirred at room temperature for 16 h. Then, iodine (381 mg, 1.50 mmol) was added to this mixture and stirred at room temperature for 1 h. After ethyl acetate (10 mL) was added, the reaction mixture was washed with water, and brine. The extracted organic layer was dried over Na_2SO_4 and concentrated in vacuo. The crude product was purified by HPLC to give bis(4-iodophenyl)methanone (**25**) in 25% yield (32.5 mg).

Spectrum data of the products.

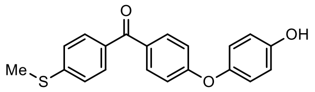

(4-(4-Hydroxyphenoxy)phenyl)(4-(methylthio)phenyl)methanone (4). A colorless solid. Rf = 0.55 (hexane:AcOEt = 1:1 (v/v)). mp = 141 – 144 °C. ^{1}H NMR (600 MHz, $CDCl_3$) δ 2.53 (s, 3H, SCH_3), 5.55 (br, 1H, OH), 6.87 (AA'BB', 2H, aromatic), 6.96-6.98 (m, 4H, aromatic), 7.29 (AA'BB', 2H, aromatic), 7.72 (AA'BB', 2H, aromatic), 7.76 (AA'BB', 2H, aromatic). ^{13}C NMR (151 MHz, $CDCl_3$) δ 14.9, 116.3, 116.6, 121.8, 124.9, 130.5, 131.5, 132.3, 134.0, 145.0, 148.5, 152.9, 162.6, 195.0. IR (neat) 1640, 1592, 1508, 1470, 1396, 1312, 1290, 1273, 1234, 1167, 1151, 1087, 930, 839, 770, 755, 675, 626 cm^{-1}. HRMS calcd for $C_{20}H_{16}O_3SNa$ (M + Na) 359.0718, found 359.0712.

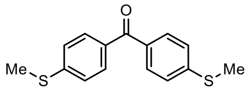

Bis(4-(methylthio)phenyl)methanone (5).[3,4,5,6] This compound is known (CAS registry number: 63084-99-1). ^{1}H NMR (600 MHz, $CDCl_3$) δ 2.54 (s, 6H, SCH_3), 7.28 (d, J = 8.0 Hz, 4H, aromatic), 7.71 (d, J = 8.0 Hz, 4H, aromatic). ^{13}C NMR (151 MHz, $CDCl_3$) δ 14.9, 124.8, 130.5, 133.7, 145.0, 195.0.

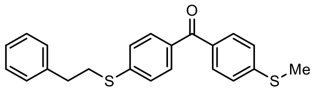

4-Methylthiophenyl-4'-phenethylthiophenyl-methanone (8). A colorless solid. Rf = 0.35 (hexane:AcOEt = 10:1 (v/v)). mp = 75 – 87 °C. ^{1}H NMR (600 MHz, $CDCl_3$) δ 2.54 (s, 3H, SCH_3), 3.00 (t, J = 8.1 Hz, 2H, CH_2), 3.26 (t, J = 8.1 Hz, 2H, CH_2), 7.23-7.26 (m, 3H, aromatic), 7.29 (AA'BB', 2H, aromatic), 7.32 (AA'BB', 2H, aromatic), 7.35 (AA'BB', 2H, aromatic), 7.70-7.73 (m, 4H, aromatic). ^{13}C NMR (151 MHz, $CDCl_3$) δ 14.9, 33.6, 35.2, 124.8, 126.5, 126.7, 128.6, 128.7, 130.5, 130.6, 133.8, 134.4, 139.8, 143.3, 145.1, 195.0. IR (neat) 3026, 2922, 1642, 1589, 1397, 1313, 1291, 1184, 1087, 1014, 963, 930, 845, 819, 750, 723, 698, 671 cm^{-1}. HRMS calcd for $C_{22}H_{20}OS_2Na$ (M + Na) 387.0853, found 387.0848.

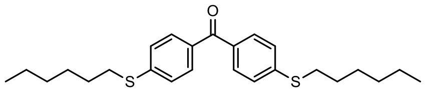

Bis(4-(*n*-hexylthio)phenyl)methanone (9).[7] This compound is known (CAS registry number: 854021-34-4). A colorless solid. Rf = 0.17 (hexane:AcOEt = 98:2 (v/v)). ^{1}H NMR (600 MHz, $CDCl_3$) δ 0.88 (t, J = 6.7 Hz, 6H, methyl), 1.28-1.32 (m, 8H, methylene), 1.44 (tt, 4H, J = 7.5 Hz, methylene), 1.69 (tt, J = 7.5 Hz, 4H, methylene), 2.98 (t, J = 7.5 Hz, 4H, SCH_2), 7.30 (d, 8.3 Hz, 4H,

aromatic), 7.68 (d, *J* = 8.3 Hz, 4H, aromatic). ^{13}C NMR (151 MHz, $CDCl_3$) δ 14.1, 22.6, 28.6, 28.8, 31.4, 32.0, 126.1, 130.5, 134.1, 144.1, 194.9.

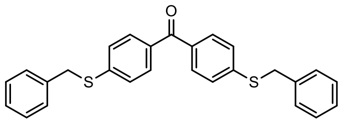

Bis(4-(phenylmethylthio)phenyl)methanone (10). This compound is known (CAS registry number: 939909-10-1). A colorless solid. ^{1}H NMR (600 MHz, $CDCl_3$) δ 4.23 (s, 4H, methylene), 7.47 (t, *J* = 7.4 Hz, 2H, aromatic), 7.31-7.34 (m, 8H, aromatic), 7.37 (d, 7.5 Hz, 4H, aromatic), 7.66 (d, *J* = 8.2 Hz, 4H, aromatic). ^{13}C NMR (151 MHz, $CDCl_3$) δ 37.5, 127.0, 127.7, 128.8, 128.9, 130.6, 134.7, 136.5, 143.5, 195.0.

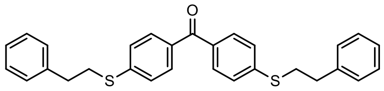

Bis(4-(2-phenylethylthio)phenyl)methanone (11). A light-brown solid. Rf = 0.35 (hexane:AcOEt = 10:1 (v/v)). mp = 100 – 103 °C. ^{1}H NMR (600 MHz, $CDCl_3$) δ 3.00 (t, *J* = 7.5 Hz, 4H, CH_2), 3.27 (t, *J* = 8.1 Hz, 4H, CH_2), 7.23-7.27 (m, 6H, aromatic), 7.33 (AA'BB'C, 4H, aromatic), 7.36 (AA'BB', 4H, aromatic), 7.72 (AA'BB', 4H, aromatic). ^{13}C NMR (151 MHz, $CDCl_3$) δ 33.6, 35.2, 126.5, 126.7, 128.6, 128.7, 130.6, 134.3, 139.8, 143.4, 195.0. IR (neat) 3029, 2925, 1731, 1642, 1588, 1496, 1454, 1398, 1316, 1291, 1184, 1087, 960, 931, 847, 749, 717, 698, 670 cm^{-1}. HRMS calcd for $C_{29}H_{26}OS_2Na$ (M + Na) 477.1323, found 477.1317.

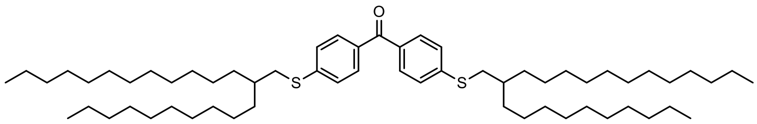

Bis(4-(2-(*n*-decyl)-*n*-tetradecylthio)phenyl)methanone (12). This chemical was isolated by column chromatography on silica-gel (hexane/ethyl acetate 96:4 to 7:3) followed by HPLC. A pale brown oil. Rf = 0.65 (hexane:AcOEt = 10:1 (v/v)). ^{1}H NMR (600 MHz, $CDCl_3$) δ 0.87 (t, *J* = 6.9 Hz, 12H, methyl), 1.22-1.33 (m, 72H, methylene), 1.36-1.45 (m, 8H, methylene), 1.66-1.71 (m, 2H, methylene), 2.97 (d, *J* = 6.4 Hz, 4H, SCH_2), 7.32 (AA'BB', 4H, aromatic), 7.69 (AA'BB', 4H, aromatic). ^{13}C NMR (151 MHz, $CDCl_3$) δ 14.1, 22.7, 26.6, 29.36, 29.38, 29.62, 29.65, 29.68, 29.69, 29.7, 29.9, 31.93, 31.94, 33.3, 37.0, 37.4, 126.3, 130.4, 134.1, 144.7, 194.9 (Several signals derived from alkyl carbons are overlapping with other signals.). IR (neat) 2923, 2853, 1653, 1590, 1458, 1398, 1313, 1287, 1179, 1088, 926, 845, 756, 734 cm^{-1}. HRMS calcd for $C_{61}H_{106}OS_2Na$ (M + Na) 941.7583, found 941.7577.

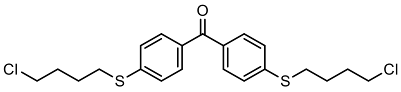

Bis(4-(4-chlorobutylthio)phenyl)methanone (13). This chemical was isolated by HPLC. A brown solid. Rf = 0.25 (hexane:AcOEt = 10:1 (v/v)). mp = 53 – 56 °C. ^{1}H NMR (600 MHz, $CDCl_3$) δ 1.86-1.91 (m, 4H, methylene), 1.93-1.98 (m, 4H, methylene), 3.04 (t, *J* = 7.1 Hz, 4H, SCH_2), 3.57 (d, *J* = 6.4 Hz, 4H, ClC*H*$_2$), 7.33 (AA'BB', 4H, aromatic), 7.71 (AA'BB', 4H, aromatic). ^{13}C NMR (151 MHz, $CDCl_3$) δ 26.1, 31.4, 31.5, 44.3, 126.6, 130.5, 134.4, 143.3, 194.8. IR (neat) 2954, 2868, 1642, 1588, 1399, 1321, 1289, 1180, 1090, 1013, 928, 846, 752, 718, 671, 651 cm^{-1}. HRMS calcd for $C_{21}H_{24}Cl_2OS_2Na$ (M + Na) 449.0543, found 449.0538.

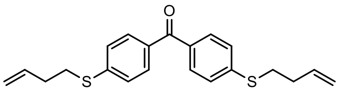

Bis(4-(3-butenylthio)phenyl)methanone (14). A pale yellow solid. Rf = 0.35 (hexane:AcOEt = 10:1 (v/v)). mp = 51 – 53 °C. ^{1}H NMR (600 MHz, $CDCl_3$) δ 2.44-2.48 (m, 4H, CH_2), 3.07 (t, *J* = 7.6 Hz, 4H, SCH_2), 5.09 (dq, *J* = 1.4, 10.1 Hz, 2H, ethenyl), 5.13 (dq, *J* = 1.6, 17.1 Hz, 2H, ethenyl), 5.13 (ddt, *J* = 6.7, 10.4, 17.1 Hz, 2H, ethenyl), 7.33 (AA'BB'C, 4H, aromatic), 7.71 (AA'BB', 4H, aromatic). ^{13}C NMR (151 MHz, $CDCl_3$) δ 31.4, 33.0, 116.8, 126.4, 130.6, 134.3, 135.9, 143.5, 195.0. IR (neat) 3081, 3980, 2931, 1638, 1588, 1429, 1398, 1316, 1292, 1183, 1087, 991, 922, 852, 818, 751, 675, 635 cm^{-1}. HRMS calcd for $C_{21}H_{22}OS_2Na$ (M + Na) 377.1010, found 377.1004.

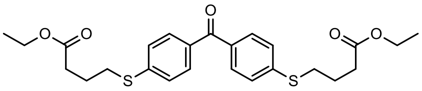

Bis(4-(3-ethoxycarbonylpropylthio)phenyl)methanone (15). A yellow oil. Rf = 0.25 (hexane:AcOEt = 10:1 (v/v)). ^{1}H NMR (600 MHz, $CDCl_3$) δ 1.26 (t, *J* = 7.2 Hz, 6H, CH_3), 2.03 (quint, *J* = 7.2 Hz, 4H, CH_2), 2.49 (t, *J* = 7.4 Hz, 4H, CH_2), 3.06 (t, *J* = 7.0 Hz, 4H, SCH_2), 4.14 (q, *J* = 7.2 Hz, 4H, OCH_2), 7.35 (AA'BB', 4H, aromatic), 7.70 (AA'BB', 4H, aromatic). ^{13}C NMR (151 MHz, $CDCl_3$) δ 14.3, 24.1, 31.4, 32.9, 60.6, 126.6, 130.6, 134.4, 143.1, 172.8, 194.9. IR (neat) 2979, 1731, 1650, 1589, 1398, 1374, 1314, 1288, 1180, 1142, 1088, 1037, 927, 848, 756, 674 cm^{-1}. HRMS calcd for $C_{25}H_{30}O_5S_2Na$ (M + Na) 497.1432, found 497.1427.

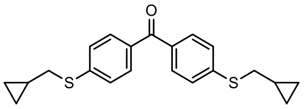

Bis(4-(cyclopropylmethylthio)phenyl)methanone (16). A colorless solid. Rf = 35 (hexane:AcOEt

= 10:1 (v/v)). mp = 110 – 112 °C. ^{1}H NMR (600 MHz, $CDCl_3$) δ 0.30-0.33 (m, 4H, cCH_2), 0.62-0.65 (m, 4H, cCH_2), 1.08-1.14 (m, 2H, cCH), 2.96 (d, J = 7.0 Hz, 4H, SCH_2), 7.34 (AA'BB', 4H, aromatic), 7.69 (AA'BB', 4H, aromatic). ^{13}C NMR (151 MHz, $CDCl_3$) δ 5.83, 10.1, 38.0, 126.4, 130.5, 134.2, 144.2, 195.0. IR (neat) 2979, 1737, 1642, 1587, 1316, 1290, 1088, 1013, 925, 835, 751 cm^{-1}. HRMS calcd for $C_{21}H_{22}OS_2Na$ (M + Na) 377.1010, found 377.1007.

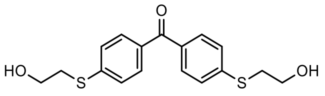

Bis(4-(2-hydroxyethylthio)phenyl)methanone (17).[8,9] This product was isolated from the column chromatography followed by recrystallization from ethyl acetate/hexane. This compound is known (CAS registry number: 345290-68-8). A pale brown powder. Rf = 0.36 (hexane:AcOEt = 1:1 (v/v)). ^{1}H NMR (600 MHz, $CDCl_3$/MeOD(1/9, v/v)) 3.18 (t, J = 6.7 Hz, 4H, methylene), 3.77 (t, J = 6.7 Hz, 4H, methylene), 7.42 (dd, J = 1.7, 8.5 Hz, 4H, aromatic), 7.68 (dd, J = 1.4, 8.5 Hz, 4H, aromatic). ^{13}C NMR (151 MHz, $CDCl_3$/MeOD(1/9, v/v)) 33.8, 60.0, 126.1, 130.2, 134.0, 143.9, 195.4.

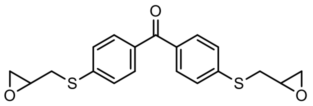

Bis[4-(oxiranylmethylthio)phenyl]methanone (18). This chemical was isolated by column chromatography on silica-gel (hexane/ethyl acetate 9:1 to 6:4) followed by HPLC. A colorless solid. Rf = 0.45 (hexane:AcOEt = 1:1 (v/v)). mp = 78 – 84 °C. ^{1}H NMR (600 MHz, $CDCl_3$) 2.65 (dd, J = 2.6, 4.8 Hz, 2H, methylene), 2.83-2.85 (m, 2H, methylene), 3.14 (dd, J = 6.8, 15.8 Hz, 2H, methylene), 3.22-3.25 (m, 4H), 7.43 (AA'BB', 4H, aromatic), 7.71 (AA'BB', 4H, aromatic). ^{13}C NMR (151 MHz, $CDCl_3$) 35.0, 47.3, 50.7, 127.4, 130.6, 134.9, 142.3, 194.8. IR (neat) 1639, 1589, 1398, 1316, 1293, 1186, 1085, 931, 847, 814, 748, 670 cm^{-1}. HRMS calcd for $C_{19}H_{19}O_3S_2$ (M + H) 359.0776, found 359.0770.

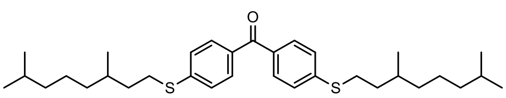

Bis(4-(3,7-dimethyl-*n*-octylthio)phenyl)methanone (19). A brown oil. Rf = 0.40 (hexane:AcOEt = 10:1 (v/v)). ^{1}H NMR (600 MHz, $CDCl_3$) δ 0.86 (d, J = 6.6 Hz, 12H, methyl), 0.94 (d, J = 6.6 Hz, 6H, methyl), 1.12-1.16 (m, 6H, methylene), 1.22-1.34 (m, 6H, methylene), 1.50-1.56 (m, 4H, methylene), 1.58-1.63 (m, 2H, methylene), 1.69-1.75 (m, 2H, methylene), 2.97 (ddd, J = 6.3, 9.6, 12.5 Hz, 2H, SCH_2), 3.05 (ddd, J = 6.3, 9.6, 12.5 Hz, 2H, SCH_2), 7.32 (AA'BB', 4H, aromatic), 7.70 (AA'BB', 4H,

aromatic). ^{13}C NMR (151 MHz, $CDCl_3$) δ 19.4, 22.6, 22.7, 24.7, 28.0, 30.0, 32.4, 35.9, 36.9, 39.2, 126.2, 130.5, 134.2, 144.1, 194.9. IR (neat) 2954, 2925, 1651, 1589, 1464, 1399, 1312, 1287, 1179, 1088, 926, 845, 823, 755, 673, 640 cm^{-1}. HRMS calcd for $C_{33}H_{50}OS_2Na$ (M + Na) 549.3201, found 549.3196.

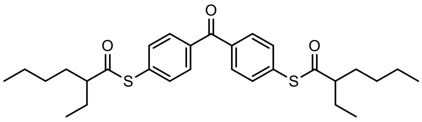

Bis(4-(1-ethyl-*n*-penthylcarbonylthio)phenyl)methanone (20). A colorless oil. Rf = 0.60 (hexane:AcOEt = 10:1 (v/v)). ^{1}H NMR (600 MHz, $CDCl_3$) δ 0.92 (t, *J* = 7.1 Hz, 6H, methyl), 1.00 (t, *J* = 7.4 Hz, 6H, methyl), 1.32-1.38 (m, 8H, methylene), 1.51-1.65 (m, 4H, methylene), 1.72-1.81 (m, 4H, methylene), 2.59-2.63 (m, 2H, methyne), 7.54 (AA'BB', 4H, aromatic), 7.83 (AA'BB', 4H, aromatic). ^{13}C NMR (151 MHz, $CDCl_3$) δ 11.8, 13.9, 22.7, 26.0, 29.5, 32.2, 56.3, 130.4, 133.7, 133.9, 137.5, 195.1, 200.3. IR (neat) 2960, 2931, 2873, 1707, 1663, 1591, 1459, 1396, 1307, 1282, 1178, 1148, 976, 926, 853, 820, 758, 710, 675 cm^{-1}. HRMS calcd for $C_{29}H_{38}O_3S_2Na$ (M + Na) 521.2160, found 521.2155.

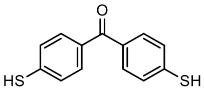

Di(4-mercaptophenyl)methanone (21). This compound is known (CAS registry number: 37089-83-1). ^{1}H NMR (600 MHz, $CDCl_3$) δ 3.63 (s, 2H, mercapto), 7.32 (AA'BB', 4H, aromatic), 7.65 (AA'BB', 4H, aromatic). ^{13}C NMR (151 MHz, $CDCl_3$) δ 128.1, 130.8, 134.5, 138.0, 194.7.

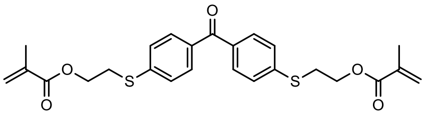

[{Carbonylbis(4,1-phenylene)}bis(sulfanediyl)]bis(ethane-2,1-diyl) bis(2-methylacrylate) (22).[S8,S9] This compound is known (CAS registry number: 104583-82-6). ^{1}H NMR (600 MHz, $CDCl_3$) δ 1.93 (dd, *J* = 0.9, 1.5 Hz, 6H, methyl), 3.30 (t, *J* = 7.0 Hz, 4H, methylene), 4.39 (t, *J* = 7.0 Hz, 4H, methylene), 5.58-5.59 (m, 2H, methylene), 6.09-6.10 (m, 2H, methylene), 7.43 (AA'BB', 4H, aromatic), 7.72 (AA'BB', 4H, aromatic). ^{13}C NMR (151 MHz, $CDCl_3$) δ 18.3, 30.8, 62.8, 126.2, 127.0, 130.7, 134.8, 135.9, 142.1, 167.2, 194.7.

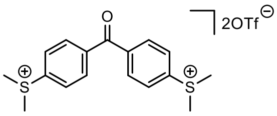

(Carbonylbis(4,1-phenylene))bis(dimethylsulfonium) bistriflate (24). A colorless solid. mp = 152 – 160 °C. ^{1}H NMR (600 MHz, acetone-d_6) δ 3.60 (s, 12H, SCH_3), 8.13 (AA'BB', 4H, aromatic), 8.41 (AA'BB', 4H, aromatic). ^{13}C NMR (151 MHz, acetone-d_6) δ 29.0, 122.3 (q, J = 322 Hz, triflate), 131.3, 131.8, 132.3, 141.9, 194.2. IR (neat) 1673, 1259, 1165, 1027, 932, 859, 756, 633 cm^{-1}. HRMS calcd for $C_{18}H_{20}O_4F_3S^+$ [M − OTf]$^+$: 453.0470, found 453.0471.

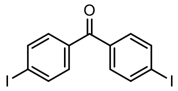

Bis(4-iodophenyl)methanone (25).[10] This compound is known (CAS registry number: 5630-56-8). ^{1}H NMR (600 MHz, $CDCl_3$) δ 7.49 (AA'BB', 4H, aromatic), 7.86 (AA'BB', 4H, aromatic). ^{13}C NMR (151 MHz, acetone-d_6) δ 100.5, 131.3, 136.4, 137.8, 1951.

Experiments for the polymer synthesis.

Polymerization of 4,4'-diiodobenzophenone (25) with 2,2-bis(4-hydroxyphenyl)propane (26)

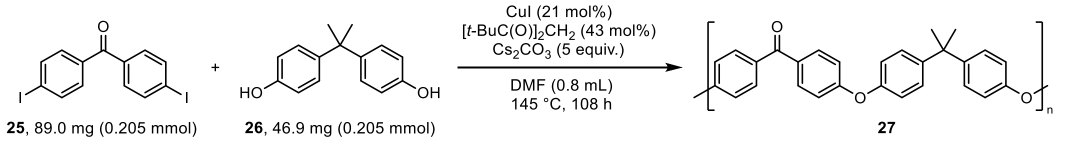

To a mixture of **25** (89.0 mg, 0.205 mmol) and 2,2-bis(4-hydroxyphenyl)propane (46.9 mg, 0.205 mmol) was added copper iodide (8.4 mg, 0.044 mmol), cesium carbonate (324.3 mg, 0.995 mmol), *N,N*-dimethylformamide (DMF, 0.8 mL), and dipivaloylmethane (16.3 mg, 0.088 mmol) in a 3 mL vial under argon atmosphere. The vial was closed with a screw cap, and the mixture was stirred at 145 °C for 108 h. After the liquid mixture was cooled down to room temperature. 1M HCl (1.5 mL) and diethyl ether (1.0 mL) were added to the reaction mixture. The generated precipitate was filtered and washed methanol/water/acetone and dried in vacuo. The desired polymer was obtained (72.1 mg, 87%) as a pale yellow solid by reprecipitation from CH_2Cl_2/MeOH and washing with Et_2O followed by dried in vacuo at 70 °C.

Poly[oxy-1,4-phenylenecarbonyl-1,4-phenyleneoxy-1,4-phenylene(1-methylethylidene)-1,4-phenylene]. This polymer is known (CAS registry number: 41205-96-3).[11] ^{1}H NMR (600 MHz, $CDCl_3$) δ 1.71 (s, 6H, methyl), 6.99 (d, J = 8.3 Hz, 4H, aromatic), 7.02 (d, J = 8.4 Hz, 4H, aromatic), 7.26 (d, J = 8.0 Hz, 4H, aromatic), 7.78 (d, J = 8,2 Hz, 4H, aromatic). ^{13}C NMR (151 MHz, $CDCl_3$) δ 31.0, 42.4, 117.1, 119.6, 128.3, 132.18, 132.22, 146.7, 153.5, 161.4, 194.3. IR (neat) 2965, 2937, 1652, 1592, 1498, 1307, 1278, 1160, 1014, 928, 873 cm^{-1}. M_w = 24039, M_n = 6891, PDI = 3.49.

Of note, employed PEEK could not be analyzed by using the Tosoh HLC-8321GPC/HT with TSKgel GMH_{HR}-H (S) HT2 column at 220 °C (1-Chloronaphthalene as the eluent) due to insolubility toward organic solvent even if 1-chloronaphthalene at 220 °C.

Polymerization of 4,4'-dimercaptobenzophenone (21) with azelaoyl chloride (28)

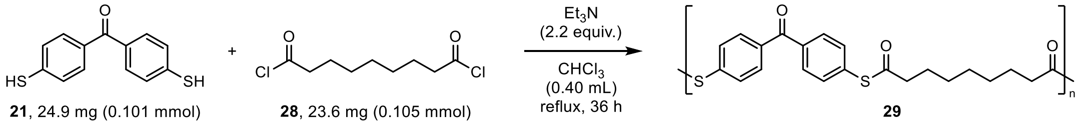

This method is based on the literature procedure.[12] Triethylamine (22.3 mg, 0.221 mmol) was added to **21** (24.9 mg, 0.101 mmol) in chloroform (0.4 mL) in a 10 mL test tube in an nitrogen atmosphere. After the mixture was stirred at room temperature for 10 min, azelaoyl chloride (23.6 mg, 0.105 mmol) was added to the resulting yellow solution and stirred at 75 °C (reflux) for 36 h.

After the liquid colorless reaction mixture was cooled down to room temperature, methanol (0.05 mL) was added to this mixture and stirred at room temperature for 1 h. The mixture was poured into methanol/acetone (1:1, 0.01 mL) solution to precipitate a colorless solid. This solid was washed with methanol, water, and acetone, and dried in vacuo at 60 °C to obtain colorless solid polymer (37.8 mg, 94%).

Poly[thio-1,4-phenylenecarbonyl-1,4-phenyleneothio-azelaoyl]. ^{1}H NMR (600 MHz, $CDCl_3$) δ 1.38-1.43 (m, 6H, methylene), 1.74 (tt, J = 7.2, 7.6 Hz, 4H, methylene), 2.70 (t, J = 7.5 Hz, 4H, methylene), 7.54 (d, J = 8,2 Hz, 4H, aromatic), 7.82 (d, J = 8,2 Hz, 4H, aromatic). ^{13}C NMR (151 MHz, $CDCl_3$) δ 25.4, 28.7, 28.9, 44.0, 130.5, 133.4, 134.0, 137.5, 195.1, 196.2. IR (neat) 2935, 2858, 1704, 1654, 1590, 1396, 1308, 1283, 1014, 959, 927, 757 cm^{-1}. M_w = 49641, M_n = 24388, PDI = 2.04.

Polymerization of 4,4’-dimercaptobenzophenone (21) with isophthaloyl chloride

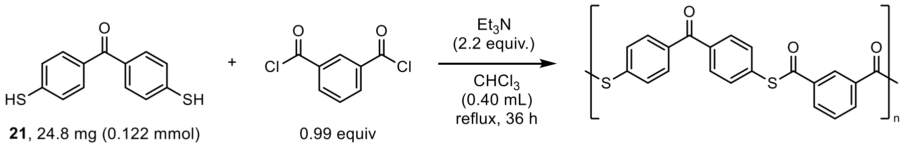

This method is based on the literature procedure.[12] Triethylamine (22.3 mg, 0.221 mmol) was added to **21** (24.8 mg, 0.101 mmol) in chloroform (0.4 mL) in a 10 mL test tube in an nitrogen atmosphere. After the mixture was stirred at room temperature for 10 min, isophthaloyl chloride (20.3 mg, 0.100 mmol) was added to the resulting yellow solution and stirred at 75 °C (reflux) for 36 h. After the reaction mixture with colorless precipitation was cooled down to room temperature, methanol (0.05 mL) was added to this mixture and stirred at room temperature for 1 h. The precipitation product was filtered, then washed with methanol, water, and acetone, and dried in vacuo at 60 °C to obtain a colorless solid (36.4 mg, 96%). This solid was insoluble toward chloroform and DMSO.

Poly[thio-1,4-phenylenecarbonyl-1,4-phenyleneothio-isophthaloyl]. IR (neat) 1671, 1589, 1394, 1279, 1240, 1145, 1094, 985, 927, 889, 845, 759, 719, 679 cm^{-1}.

Analysis of this product was attempted by using the Tosoh HLC-8321GPC/HT with TSKgel GMH_{HR}-H (S) HT2 column at 220 °C (1-Chloronaphthalene as the eluent). But, this analysis could not be done due to collapsing during this process.

Reaction of bis[4-(oxiranylmethylthio)phenyl]methanone (18) with ethylenediamine

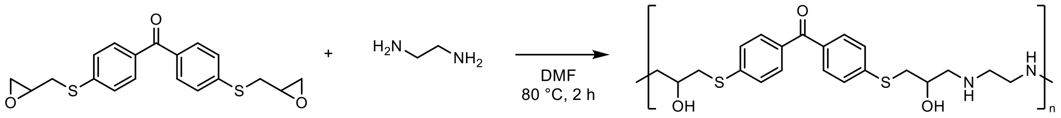

18, 30.0 mg (0.0837 mmol) **30**, 5.1 mg (0.084 mmol) proposed structure

This method is based on the literature procedure.[13] *N*,*N*-Dimethylformamide (0.10 mL) and ethylenediamine (5.1 mg, 0.084 mmol) were added to **18** (30.0 mg, 0.0837 mmol) in a 3 mL vial in an argon atmosphere. The mixture was stirred at 80 °C for 2 h. After the reaction mixture was evaporated, the precipitated product was washed with chloroform. The collected solid was dried in vacuo to get a colorless solid sample (34.5 mg). This sample was insoluble toward chloroform and DMSO.

Poly[2-hydroxypropylthio-1,4-phenylenecarbonyl-1,4-phenyleneothio-(2-hydroxypropyl)-aminoethylamino]. IR (neat) 2826, 2384, 2316, 1644, 1586, 1550, 1403, 1283, 1185, 1085, 926, 832, 754, 675 cm^{-1}.

For GPC measurement, the obtained sample (20.0 mg) was acetylated with acetic anhydride (0.16 ml) and triethylamine (0.16 ml) under 80 °C at 39 h. The collected orange solid was washed with $NaHCO_3$ aq. / methanol / acetone, and dried in vacuo at 60 °C. The obtained solid sample was insoluble toward chloroform and DMSO (even at 100 °C).

Both samples could not be analyzed by using the Tosoh HLC-8321GPC/HT with TSKgel GMH_{HR}-H (S) HT2 column at 220 °C due to collapsing during this process.

Additional experiments.

Reaction of PEEK powder (**1**) with *N*-methylaniline was attempted based on the reported methodologies via aryl carbon-oxygen bond cleavages; *t*-Bu-P_4 catalyzed amination[14] and nickel-catalyzed protocol[15] (Supplementary Fig. 1). However, no amination products were observed in both cases.

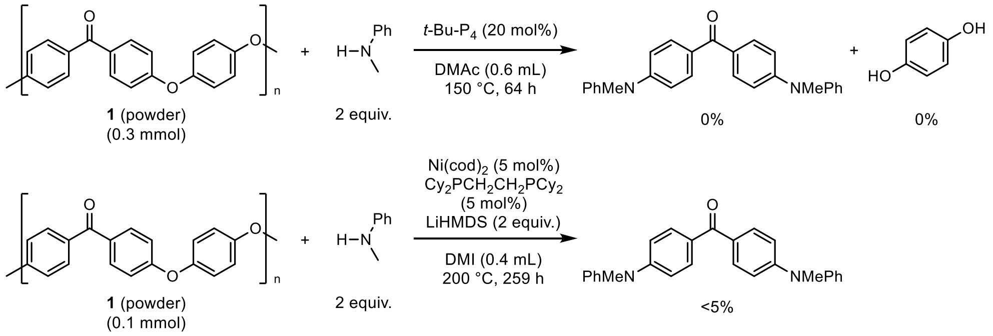

Supplementary Fig. 1 | Examining Reaction of PEEK with N-methylaniline.

The reaction of **23** with 2 equiv. of 2-phenylethanethiol and sodium *tert*-butoxide at 150 ºC was examined for longer reaction time (16 h). As a result, the yield of final product **5** was increased to 70% (Supplementary Fig. 2). In addition, styrene was generated in 45% yield. On the other hand, this reaction of **23** with 2-phenylethanethiol was attempted at 28 ºC for 16 h, followed by the quench at room temperature to give **11** and anisole in both 95% yields, respectively (Supplementary Fig. 3).

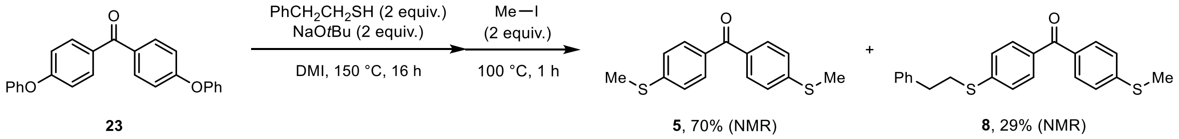

Supplementary Fig. 2 | Reaction of 4,4’-diphenoxy-benzophenone with 2 equiv. of 2-phenylethanethiol at 150 ºC for 16 h.

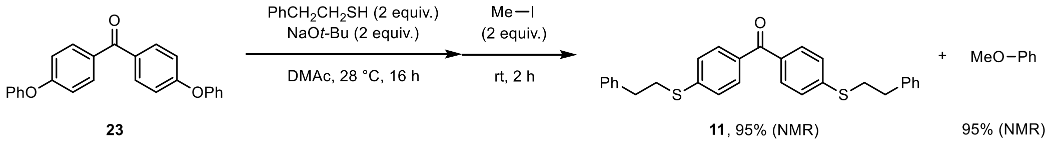

Supplementary Fig. 3 | Reaction of 4,4’-diphenoxy-benzophenone with 2 equiv. of 2-phenylethanethiol at 28 ºC.

It is known that the combination between elemental sulfur and base generate $S^{\cdot -}$ radical anion.[16,17] To verify the possibility that the sulfur radical anion react with PEEK, the reaction of PEEK with elemental sulfur and sodium *tert*-butoxide was examined (Supplementary Fig. 4).[18] In this case, ketone type comonomer **4'** was afforded in low yield whereas carbonyl-reduced comonomers **B** were observed mainly. Especially, final depolymerization product **5** and its reduced **A** were hardly generated. Also, the reaction of PEEK with Na_2S and TEMPO was attempted. But no monomer products were detected (Supplementary Fig. 5). These results suggest that the sulfur radical anion methodology is not suitable for the depolymerization of PEEK, demonstrating that sulfur radical anion species is not generated during the depolymerization of PEEK by a thiol and a base.

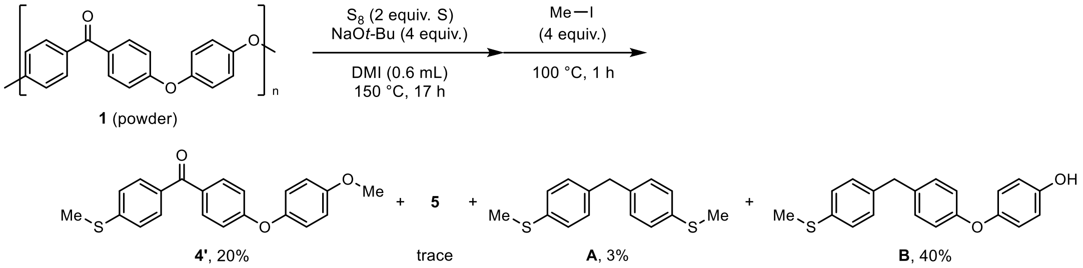

Supplementary Fig. 4 | Reaction of PEEK with sulfur and sodium *tert*-butoxide.

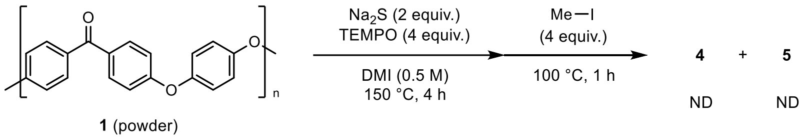

Supplementary Fig. 5 | Reaction of PEEK with sodium sulfide in the presence of TEMPO.

The reaction of **11** with 2 equiv. of sodium *tert*-butoxide at 150 ºC for 16 h occurred, leading to the production of **5** and styrene in good yield (Supplementary Fig. 6). Moreover, when 2 equiv. of 2-phenylethanethiol was added to the above conditions, the intermediate **8** and di(2-phenylethyl)sulfide was observed whereas the yield of styrene was decreased. These results support the proposed two pathways from **11** to **5** in Fig. 5.

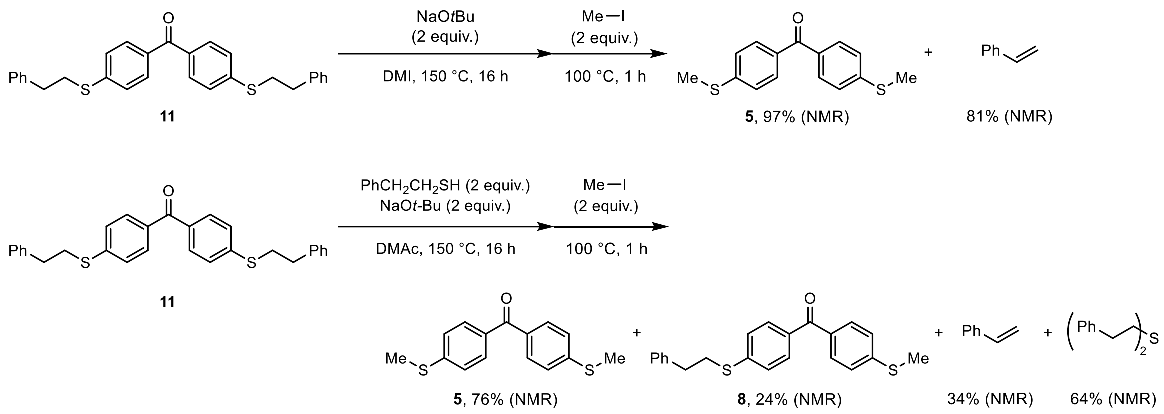

Supplementary Fig. 6 | Transformation of **11** to **5**.

We also checked the early stage of the depolymerization of PEEK pellets under optimized conditions. When the depolymerization was examined for 1 h, small amounts of **5** and **7** was observed in 7% and 24% yields, respectively (Supplementary Table 5, Entry 1). In this case, the size of remained PEEK pellets have not decreased much (Supplementary Fig. 7a and 7c). The depolymerization for 6 h increased the yields of **5** and **7** (Supplementary Table 5, Entry 2). At this time, the pellet sizes and amounts were smaller than before-use and after 1 h (Supplementary Fig. 7b and 7c). In any event, the pellet size has decreased, but the pellets remained. These observations support that the surface moieties of the PEEK materials reacted with the thiolate without dissolving.

Supplementary Table 5 | Transformation of **11** to **5**.

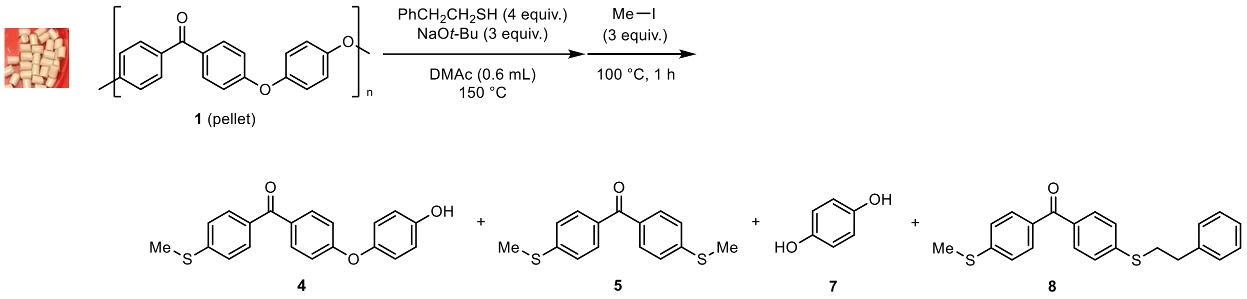

Entry	time (h)	**4** (%)	**5** (%)	**7** (%)	**8** (%)	Used Pellets (g)	Collected Pellets (g)
1	1	3	7	24	13	86.4 mg	63.6 mg
2	6	4	48	69	18	86.4 mg	25.7 mg

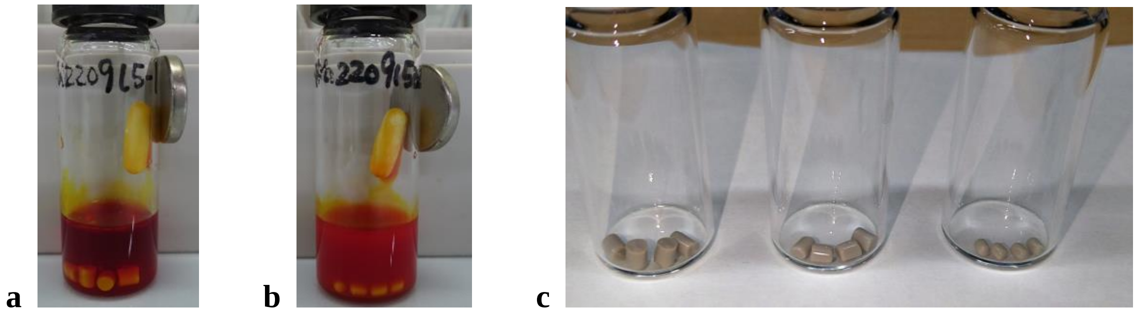

Supplementary Fig. 7 | Reaction mixture after the depolymerization of PEEK pellets before the treatment with iodomethane. **a.** 1 h (Table 6, Entry 1). **b.** 6 h (Table 6, Entry 2). **c**. Reft: PEEK pellet (84.3 mg) prior to use, Middle: collected pellets after 1 h (63.6 mg), collected pellets after 6 h (25.7 mg).

To observe the early stage of this depolymerization, we attempted the reaction of PEEK powder with 2 equiv. of Na_2S at higher temperature, 200 °C for 1 and 2 h followed by the quench with iodomethane (Supplementary Fig. 8). At least, monomers and comonomers were hardly observed. But, obtained crude solid samples as well as PEEK powder were analyzed by EGA-MS and Py-GC/MS. Supplementary Fig. 9a shows the evolution profiles of pyrolysis products from the original PEEK and crude samples from the reaction of PEEK with Na_2S for 1 or 2 h observed by EGA-MS in total ion chromatogram (TIC) mode. The TIC curve of the original PEEK clearly shows single-stage degradation between 550-650 °C. For the crude samples for both 1 h and 2 h, the gas components were detected in low temperature region at 100-600 °C. The generation of gas components from the treated samples can be due to the degradation of PEEK to afford low-weight molecules generated by random scission of the main chains. To identify and quantify the thermal degradation products of the PEEK samples, the Py-GC/MS analysis were performed. Supplementary Fig. 9b shows pyrogram of the pyrolysis products for the PEEK samples. As a result, crude samples did not contain main pyrolysis products from PEEK such as phenol, 4-hydroxyphenyl phenyl ether, and 4-hydroxybenzophenone whereas comonomer **4** was detected (Supplementary Fig. 9). Thus, these results suggests that the degradation of PEEK itself starts at the early stage even using less-reactive Na_2S than aliphatic thiol but requires a long time to generate observable products, monomers and comonomers, even at higher temperature.

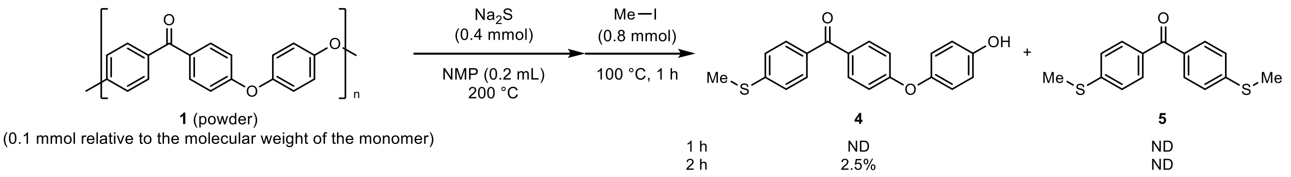

Supplementary Fig. 8 | Reaction of PEEK powder with 4 equiv. of sodium sulfide.

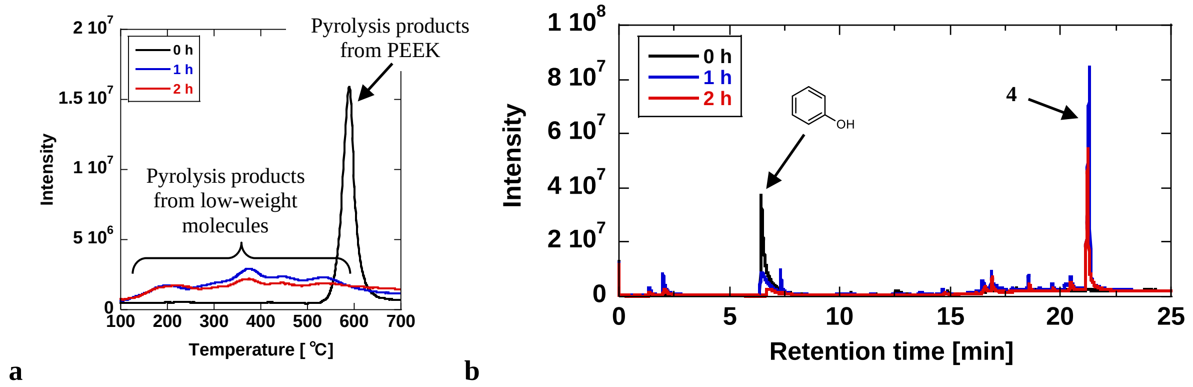

Supplementary Fig. 9 | a. Evolution profiles. **b.** Pyrograms of the pyrolysis products from the original PEEK and two crude samples shown in Supplementary Fig. 8 in TIC mode.

Next, we attempted the reaction of PEEK powder with 0.1 equiv. of Na_2S at 10 min or 1 h at 150 °C followed by the quench with iodomethane, and obtained colorless powder materials insoluble toward various solvents (Supplementary Fig. 10). These powder samples were analyzed by S *K*-edge X-ray absorption near-edge structures (XANES) at the Photon Factory BL-9A beamline. As a result, the peak derived from sulfides (~2470 eV) was observed from obtained colorless powders (with identified peaks (~2480 eV) in all cases) (Supplementary Fig. 11). In the case of PEEK resin, the former signal (~2470 eV) was not observed. At least, these results suggest that the depolymerization of PEEK was initiated on the surface moiety of PEEK. In addition, in solid-state ^{13}C NMR analysis of the obtained powders, no signal derived from the methyl carbon on sulfur was detected probably due to small amount of the methylthio group.

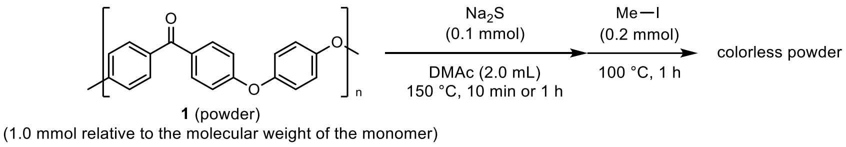

Supplementary Fig. 10 | Reaction of PEEK powder with 0.1 equiv. of sodium sulfide.

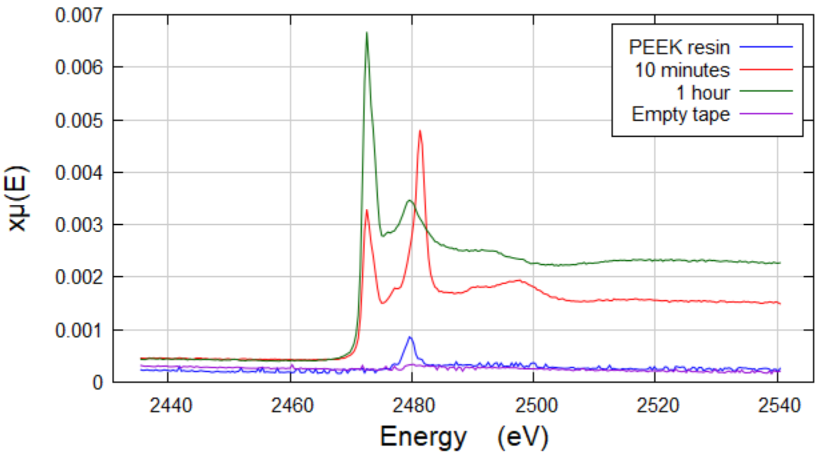

Supplementary Fig. 11 | S *K*-edge XANES spectra of PEEK resin, colorless powder from the reaction with Na_2S at 10 minutes, and 1 h. Spectrum of an empty tape used for binding the specimens is also shown to confirm negligible contribution from the experimental environment.

A DMAc solution including **23**, 2-ethylhexanethiol, and sodium *tert*-butoxide was visibly yellow, which is more colorful than other solutions without **23**, the thiol, or sodium *tert*-butoxide (Supplementary Fig. 12). This observation indicates that this yellow color of the solution containing **23**, 2-ethylhexanethiol, and sodium *tert*-butoxide is assumed to result from the formation of an electron donor–acceptor (EDA) complex formed by the association of the thiolate anion and **23**.[19-22] Similarly, surface of colorless PEEK film was turned to yellow by adding 2-ethylhexanethiol, sodium *tert*-butoxide, and DMAc (Supplementary Fig. 13c), whose color was different from the cases without sodium *tert*-butoxide or the thiol (Supplementary Fig. 13a and 13b). Moreover, the combination employing 2-pheneyethane thiol instead of 2-ethylhexanethiol with sodium *tert*-butoxide and DMAc turned the surface color of PEEK film to yellow (Supplementary Fig. 14). These results also indicate that the EDA complex is formed on the PEEK surface.

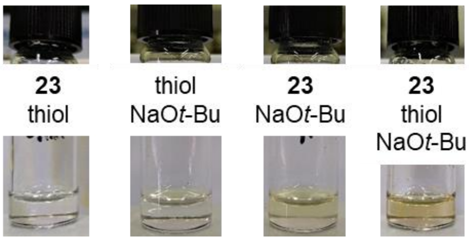

Supplementary Fig. 12 | Various combinations of **23** (0.05 mmol), 2-ethylhexanethiol (0.05 mmol), and sodium *tert*-butoxide (0.05 mmol) in DMAc (0.50 mL).

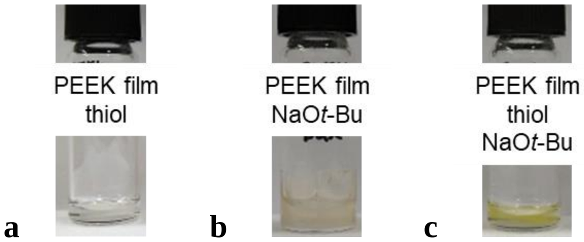

Supplementary Fig. 13 | a. Combination of PEEK film, 2-ethylhexanethiol (0.1 mmol), and DMAc (0.25 mL). **b**. Combination of PEEK film, sodium *tert*-butoxide (0.15 mmol), and DMAc (0.50 mL). **c**. Combination of PEEK film, 2-ethylhexanethiol (0.1 mmol), sodium *tert*-butoxide (0.1 mmol), and DMAc (0.25 mL).

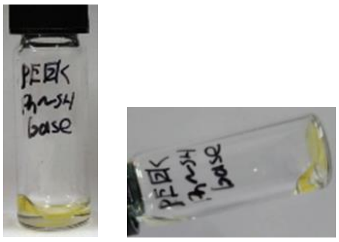

Supplementary Fig. 14 | Combination of PEEK film, 2-phenylethanethiol (0.1 mmol), and sodium *tert*-butoxide (0.1 mmol) in DMAc (0.25 mL).

Relative free energies of proposed intermediates analyzed by DFT calculations.

We investigated thermodynamic factors of the key carbon−oxygen bond and carbon−sulfur bond cleaving reactions employing model substrates by DFT calculations (Supplementary Fig. 15, 16, and 17). All DFT calculations were performed in Gaussian 09.[23] All structures were optimized in and characterized by a opt + freq calculation with solvation treatment (SMD) using DMF which is similar to the DMI, at the B3LYP/6-31G(d)* level of theory.

We checked the carbon-oxygen bond-cleaving reaction of 4-methoxybenzophenone with sodium sulfide or sodium methylthiolate (Supplementary Fig. 15). DFT calculations suggested that these processes are thermodynamically disfavored. On the contrary, when 4-(4-phenoxyphenoxy)benzophenone was used instead of 4-methoxybenzophenone, these substitution reactions were favored due to the stability of the eliminated sodium arylate.

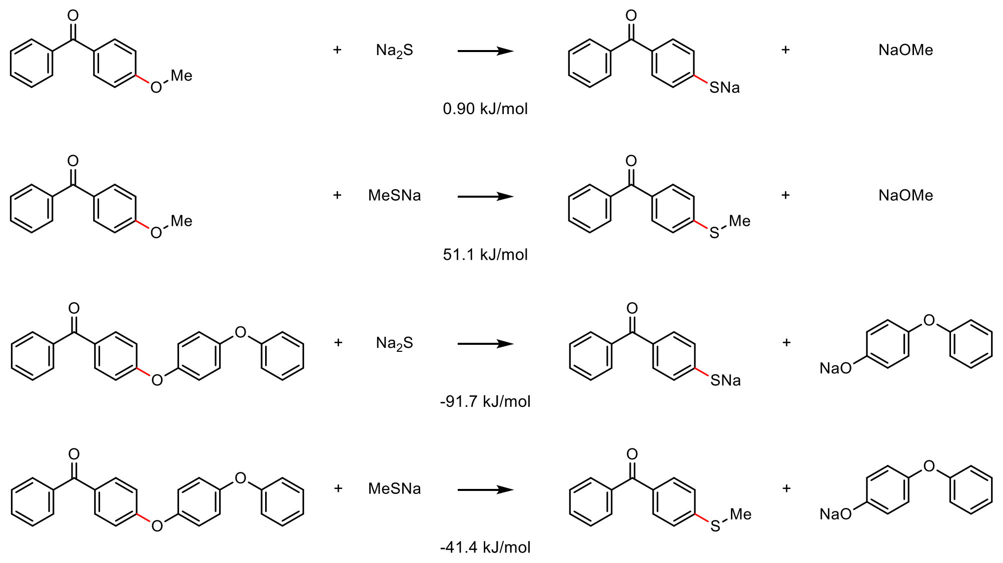

Supplementary Fig. 15 | Relative free energies (*G*, kcal mol^{-1}) from DFT calculation (RB3LYP/6-31G*) in DMF under model reactions.

Next, we checked the possibility of the depolymerization of PEEK by sulfur nucleophiles and analyzed the carbon-oxygen bond cleaving reaction and the generation of thiolates using key monomer substrates (Supplementary Fig. 16). The substitution reaction of $-OC_6H_4OH$ to alkylthio group was thermodynamically favored in a manner similar to the above case. However, the substitution of anionic aryloxy group ($-OC_6H_4ONa$) was disfavored process, supporting that the plausible comonomer showed the tolerance to the substitution to form benzophenone monomers. In this process, opposite sulfur groups hardly affect this substitution. Transformation from methylthio group to sodium arylthiolates by using sodium methyl thiolate reagent was thermodynamically

favored, demonstrating that disodium benzophenone dithiolate, (4-$NaSC_6H_4)_2CO$ (**3**) is finally formed during the depolymerization of PEEK.

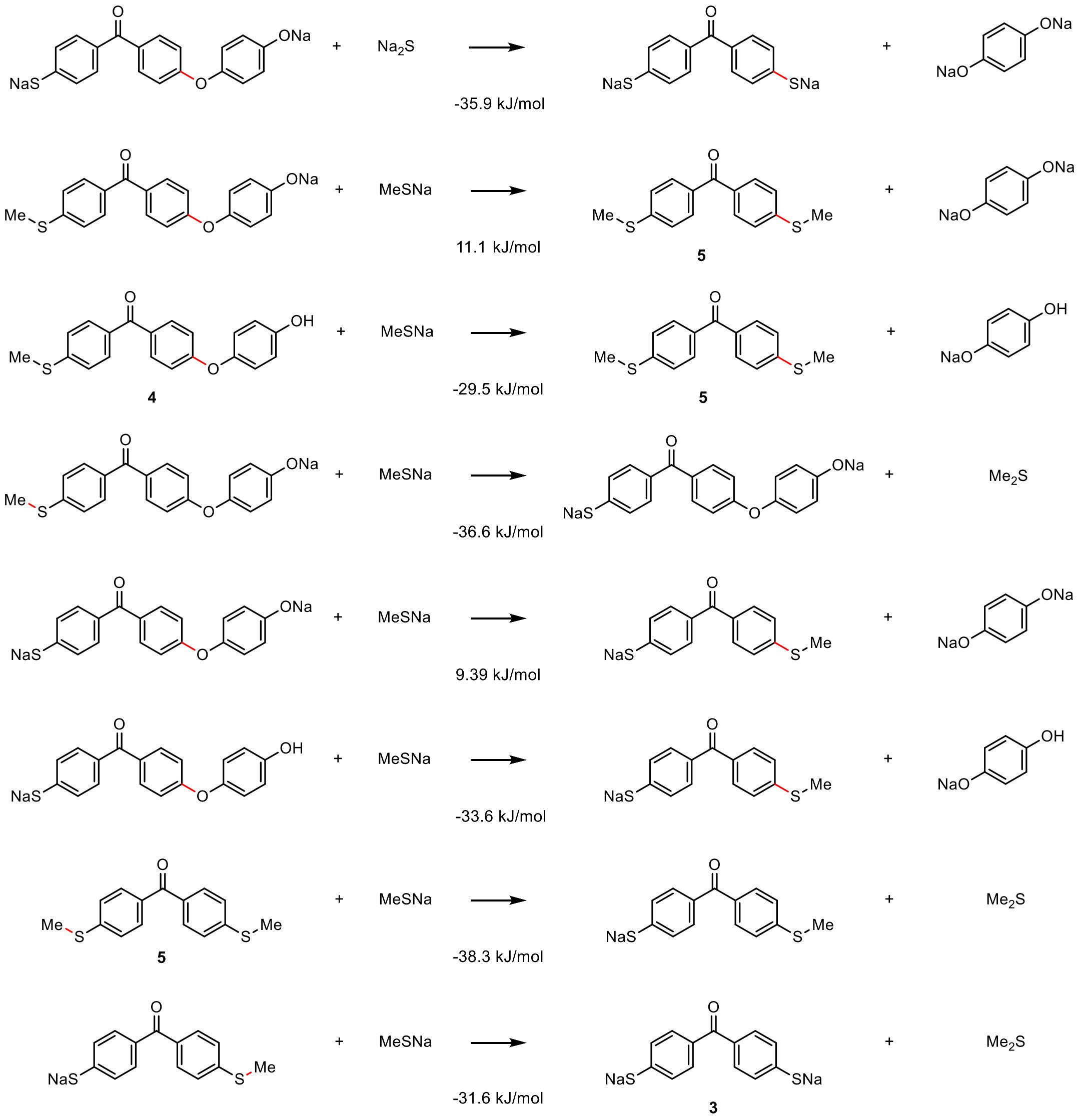

Supplementary Fig. 16 | Relative free energies (*G*, kcal mol^{-1}) of proposed intermediates and the products from DFT calculation (RB3LYP/6-31G*) in DMF.

Anion exchange reactions from sodium arylate to sodium methylthiolate were checked (Supplementary Fig. 17). In cases employing co-monomer-type sodium arylates as activating reagent for thiols, these processes were estimated to be disadvantageous thermodynamically. However, it seems that the depolymerization conditions at 150 ºC overcomes these barriers. On the other hand, the conversion employing hydroquinone disodium salt is thermodynamically favorable.

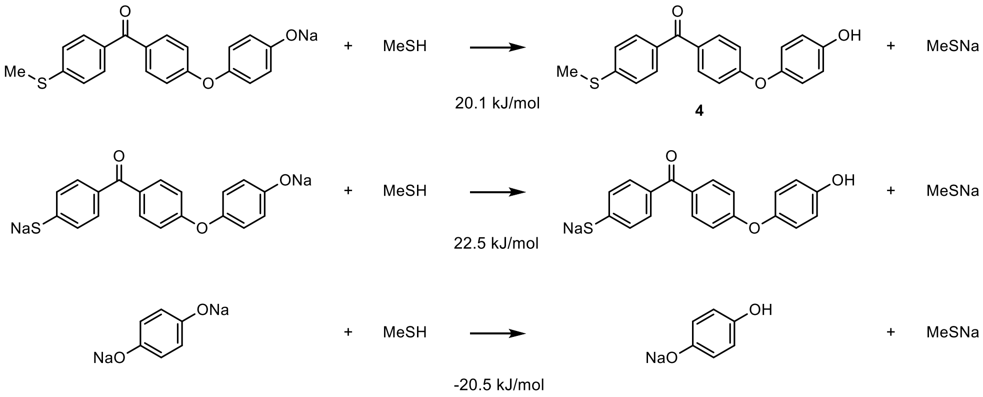

Supplementary Fig. 17 | Relative free energies (*G*, kcal mol^{-1}) from DFT calculation (RB3LYP/6-31G*) in under anion exchange reactions.

Supplementary References.

1) Minami, H., Otsuka, S., Nogi, K. & Yorimitsu, H. Palladium-Catalyzed Borylation of Aryl Sulfoniums with Diborons. *ACS Catal. 8*, 579-583 (2018).
2) Yamada, K., Yanagi, T. & Yorimitsu, H. Generation of organozinc reagents from arylsulfonium salts using a nickel catalyst and zinc dust. *Org. Lett. 22*, 9712–9718 (2020).
3) Lucas, P., El Mehdi, N., Ho, H. A., Belanger, D. & Breau, L. Expedient Synthesis of Symmetric Aryl Ketones and of Ambient-Temperature Molten Salts of Imidazole. *Synthesis* 1253-1258 (2000).
4) Gawel, P., Dengiz, C., Finke, A. D., Trapp, N., Boudon, C., Gisselbrecht, J.-P. & Diederich, F. Synthesis of Cyano-Substituted Diaryltetracenes from Tetraaryl[3]cumulenes. *Angew. Chem. Int. Ed. 53*, 4341-4345 (2014).
5) Rérat, A., Michon, C., Agbossou-Niedercorn, F. & Gosmini, C. Synthesis of Symmetrical Diaryl Ketones by Cobalt-Catalyzed Reaction of Arylzinc Reagents with Ethyl Chloroformate. *Eur. J. Org. Chem.* 4554-4560 (2016).
6) Delcaillau, T., Boehm, P. & Morandi, B. Nickel-Catalyzed Reversible Functional Group Metathesis between Aryl Nitriles and Aryl Thioethers. *J. Am. Chem. Soc. 143*, 3723-3728 (2021).
7) Improvements in or relating to the manufacture of thio- or sulphonyl-substituted basic benzhydryl-ethers and thio-ethers. United Kingdom Patent GB729619 (1955).
8) Tamura, Y. & Hayakawa, S. Method For Producing Sulfur-containing Acrylic Compound. Japanese Patent JP2001172253 (2001).
9) Tamura, Y. & Hayakawa, S. Method For Producing Sulfur-containing Acrylic Compound. Japanese Patent JP2001172255 (2001).
10) Li, Z., Siklos, M., Pucher, N., Cicha, K., Ajami, A., Husinsky, W., Rosspeintner, A., Vauthey, E., Gescheidt, G., Stampfl, J. & Liska, R. Synthesis and Structure-Activity Relationship of Several Aromatic Ketone-Based Two-Photon Initiators. *J. Polym. Sci. Part A 49*, 3688-3699 (2011).
11) Salunke, A. K., Sharma, M., Kute, V. & Banerjee, S. Synthesis of Novel Copoly(ether ether ketones): Property Evaluation and Microstructure Analysis by NMR. *J. App. Poly. Sci. 96*, 1292–1305 (2005).
12) Fukuda, N. Suzuki, M., Hirano, H., Agari, Y. & Kadota, J. Epoxy Resin Composition. Japanese Patent JP 2012144622 (2011).
13) Uneyama, J., Ogawa, R., Tsuge, A. & Endo, T. Investigation of the hardener with latent and rapid curing based on phenol-amine salts for applications to cyanate ester resins. *J. Appl. Polym. Sci. 138*, e51286 (2021).

14) Shigeno, M., Hayashi, K., Nozawa-Kumada, K. & Kondo, Y. Organic Superbase *t*-Bu-P4 Catalyzes Amination of Methoxy(hetero)arenes. *Org. Lett. 21*, 5505-5508 (2019).

15) Tobisu, M., Takahira, T., Morioka, T. & Chatani, N. Nickel-Catalyzed Alkylative Cross-Coupling of Anisoles with Grignard Reagents via C−O Bond Activation. *J. Am. Chem. Soc. 138*, 6711-6714 (2016).

16) Chivers, T. & Elder, P. Ubiquitous trisulfur radical anion: fundamentals and applications in materials science, electrochemistry, analytical chemistry and geochemistry. *Chem. Soc. Rev. 42*, 5996-6005 (2013).

17) Leghié, P., Lelieur, J. P. & Levillain, E. Comments on the mechanism of the electrochemical reduction of sulphur in dimethylformamide. *Electrochem. Commun. 4*, 406-411 (2002).

18) Zahg, G., Yi, H., Chen, H., Bian, C., Liu, C. & Lei, A. Trisulfur Radical Anion as the Key Intermediate for the Synthesis of Thiophene via the Interaction between Elemental Sulfur and NaO*t*Bu. *Org. Lett. 16*, 6156-6159 (2014).

19) Al-Khalil, S. I., Bowman, W. R., K., Gaitonde, K., Marley, M. A. & Richardson, G. D. Radical-nucleophilic substitution ($S_{RN}1$) reactions. Part 7. Reactions of aliphatic α-substituted nitro compounds. *J. Chem. Soc. Perkin Trans. 2*, 1557-1565 (2001).

20) Argüello, J. E., Schmidt, L. C. & Peñéñory, A. B. Reactivity of sulfur centered nucleophiles in photoinduced reactions with 1-bromonaphthalene. *ARKIVOC* 411-419 (2003).

21) Liu, B., Lim, C.-H. & Miyake, G. M. Visible-Light-Promoted C–S Cross-Coupling via Intermolecular Charge Transfer. *J. Am. Chem. Soc. 139*, 13616-13619 (2017).

22) Li, G., Yan, Q., Gan, Z., Li, Q., Dou, X. & Yang, D. Photocatalyst-Free Visible-Light-Promoted C(sp^2)−S Coupling: A Strategy for the Preparation of S-Aryl Dithiocarbamates. *Org. Lett. 21*, 7938-7942 (2019).

23) Frisch, M. J., Trucks, G. W., Schlegel, H. B., Scuseria, G. E., Robb, M. A., Cheeseman, J. R., Scalmani, G., Barone, V., Mennucci, B., Petersson, G. A., Nakatsuji, H., Caricato, M., Li, X., Hratchian, H. P., Izmaylov, A. F., Bloino, J., Zheng, G., Sonnenberg, J. L., Hada, M., Ehara, M., Toyota, K., Fukuda, R., Hasegawa, J., Ishida, M., Nakajima, T., Honda, Y., Kitao, O., Nakai, H., Vreven, T., Montgomery, J. A., Jr., Peralta, J. E., Ogliaro, F., Bearpark, M., Heyd, J. J., Brothers, E., Kudin, K. N., Staroverov, V. N., Kobayashi, R., Normand, J., Raghavachari, K., Rendell, A., Burant, J. C., Iyengar, S. S., Tomasi, J., Cossi, M., Rega, N., Millam, J. M., Klene, M., Knox, J. E., Cross, J. B., Bakken, V., Adamo, C., Jaramillo, J., Gomperts, R., Stratmann, R. E., Yazyev, O., Austin, A. J., Cammi, R., Pomelli, C., Ochterski, J. W., Martin, R. L., Morokuma, K., Zakrzewski, V. G., Voth, G. A., Salvador, P., Dannenberg, J. J., Dapprich, S., Daniels, A. D., Farkas, Ö., Foresman, J. B., Ortiz, J. V., Cioslowski, J. & Fox, D. J. *Gaussian 09* (Gaussian, Inc., Wallingford CT, 2009).
