# Supplementary material for: Depolymerization of robust polyetheretherketone to regenerate monomer units using sulfur reagents
Source: Commun Chem. 2023 Jan 24;6:14. doi: 10.1038/s42004-023-00814-8 (PMC9873933; doi:10.1038/s42004-023-00814-8)
Supplement: Supplementary file 3 — Description of Additional Supplementary Files [file 42004_2023_814_MOESM3_ESM.pdf]

Description of Additional Supplementary Files

File Name: Supplementary Data 1

Description: original ^{1}H and ^{13}C NMR spectra and GPC charts

File Name: Supplementary Data 2

Description: computed energy values and optimized molecular geometries
