# Supplementary material for: Depolymerization of robust polyetheretherketone to regenerate monomer units using sulfur reagents
Source: Commun Chem. 2023 Jan 24;6:14. doi: 10.1038/s42004-023-00814-8 (PMC9873933; doi:10.1038/s42004-023-00814-8)
Supplement: Supplementary file 4 — Supplementary Data 1 [file 42004_2023_814_MOESM4_ESM.pdf]

Supplementary Data 1

NMR charts of new chemicals.

^{1}H NMR (600 MHz) and ^{13}C NMR (151 MHz) spectra of **4** ($CDCl_3$)

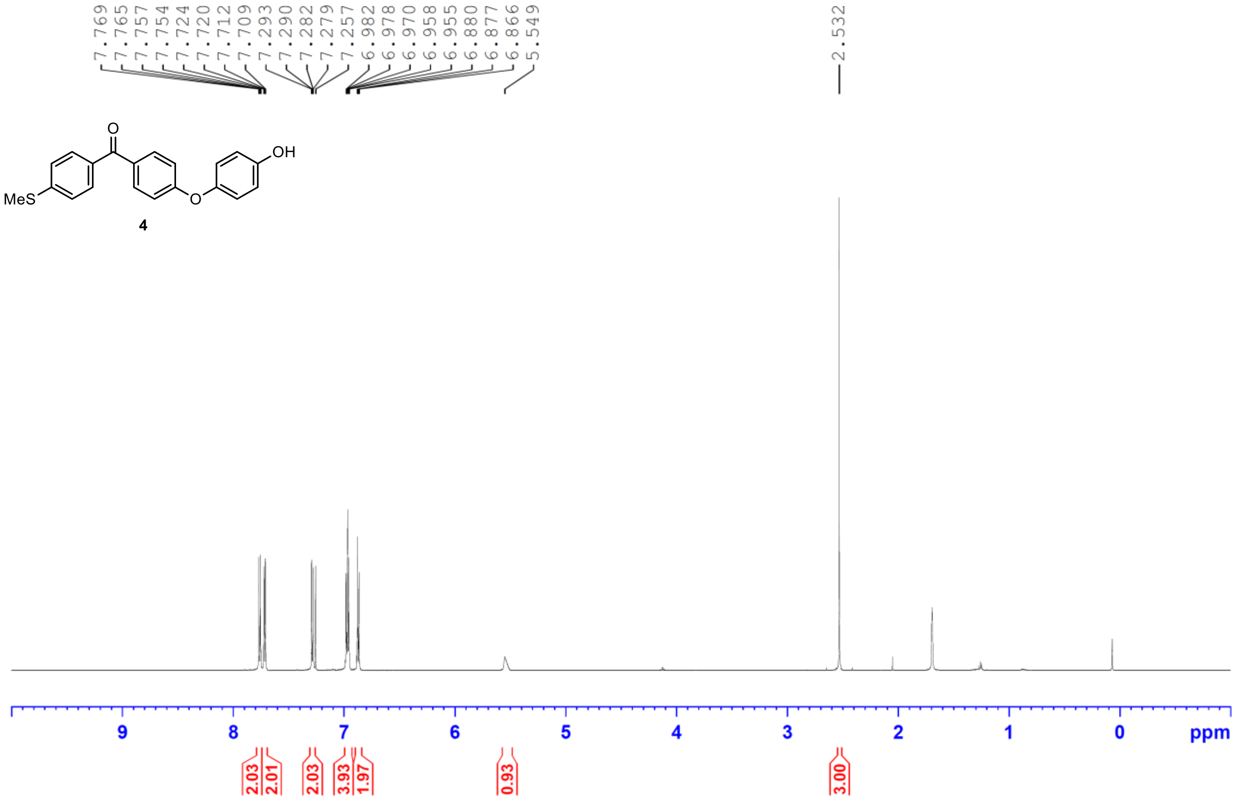

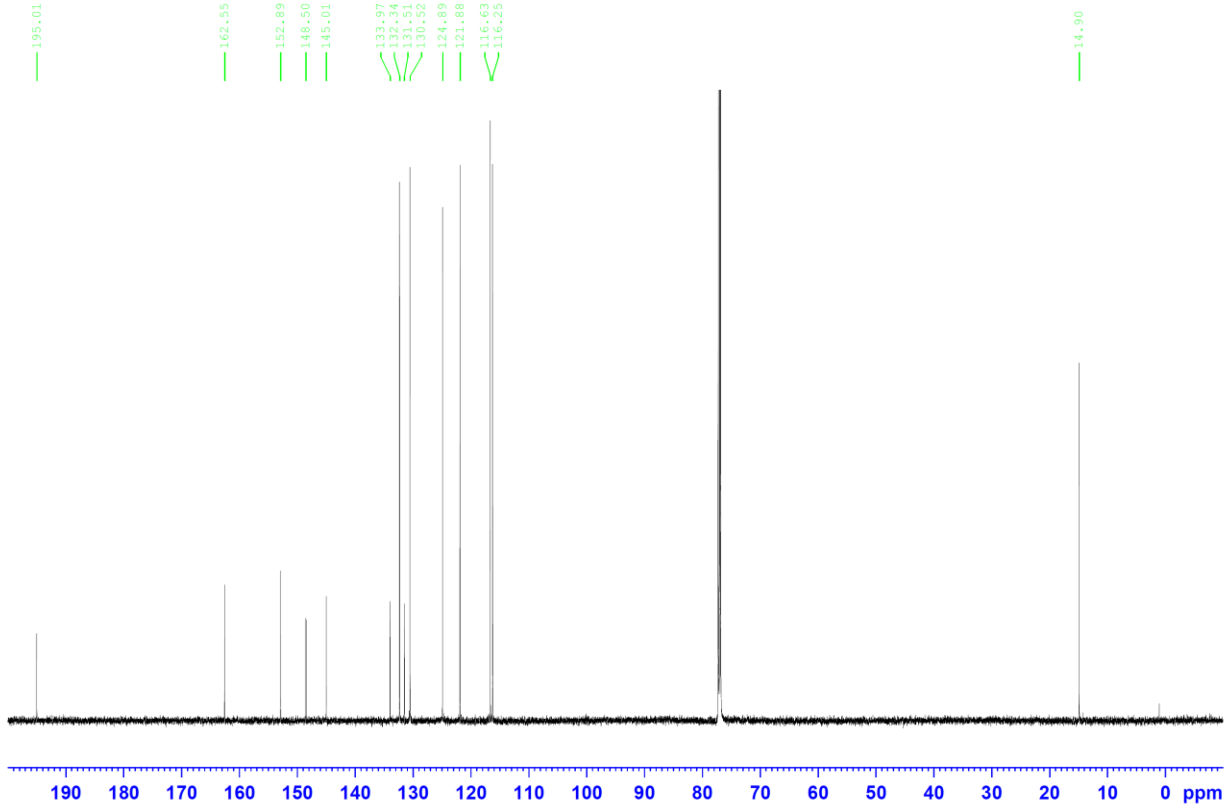

^{1}H NMR (600 MHz) and ^{13}C NMR (151 MHz) spectra of **5** ($CDCl_3$)

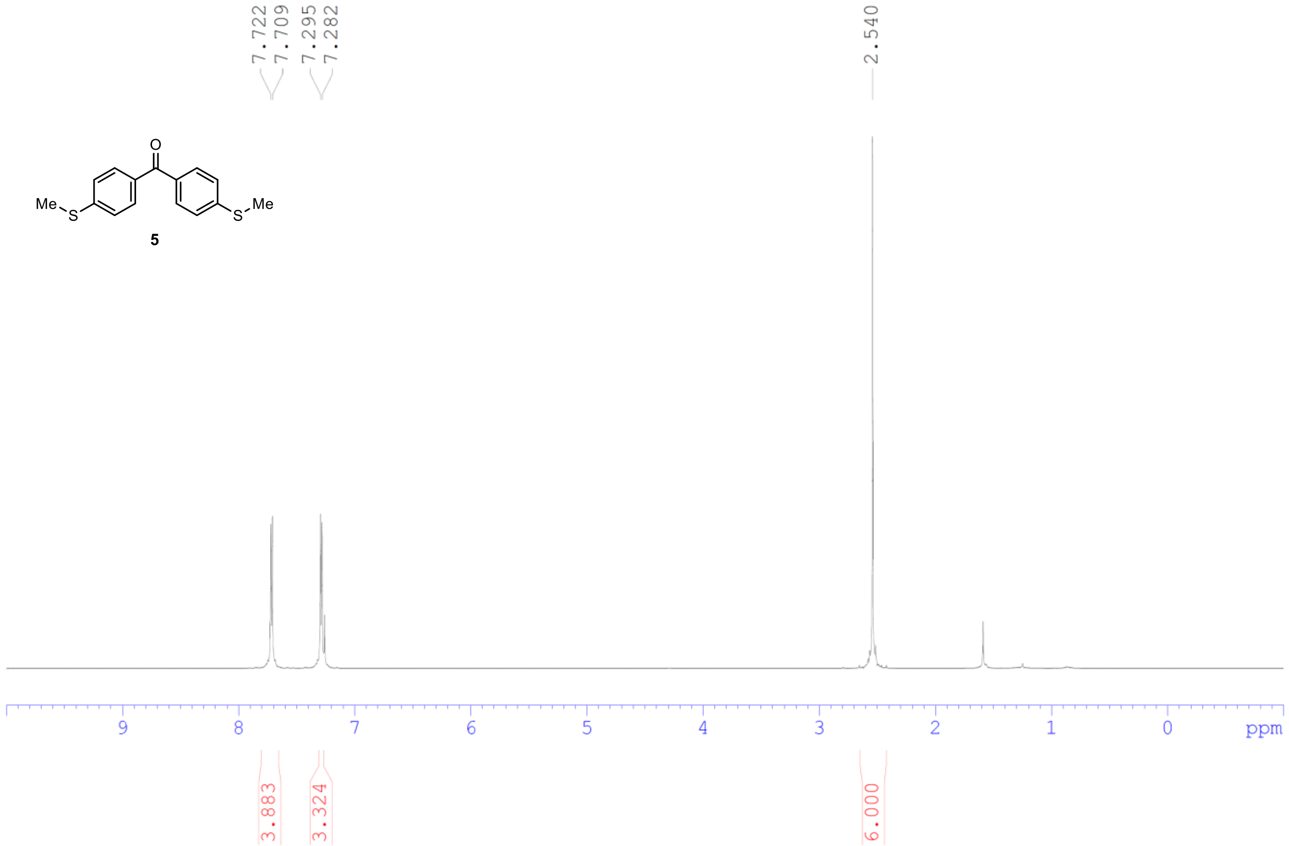

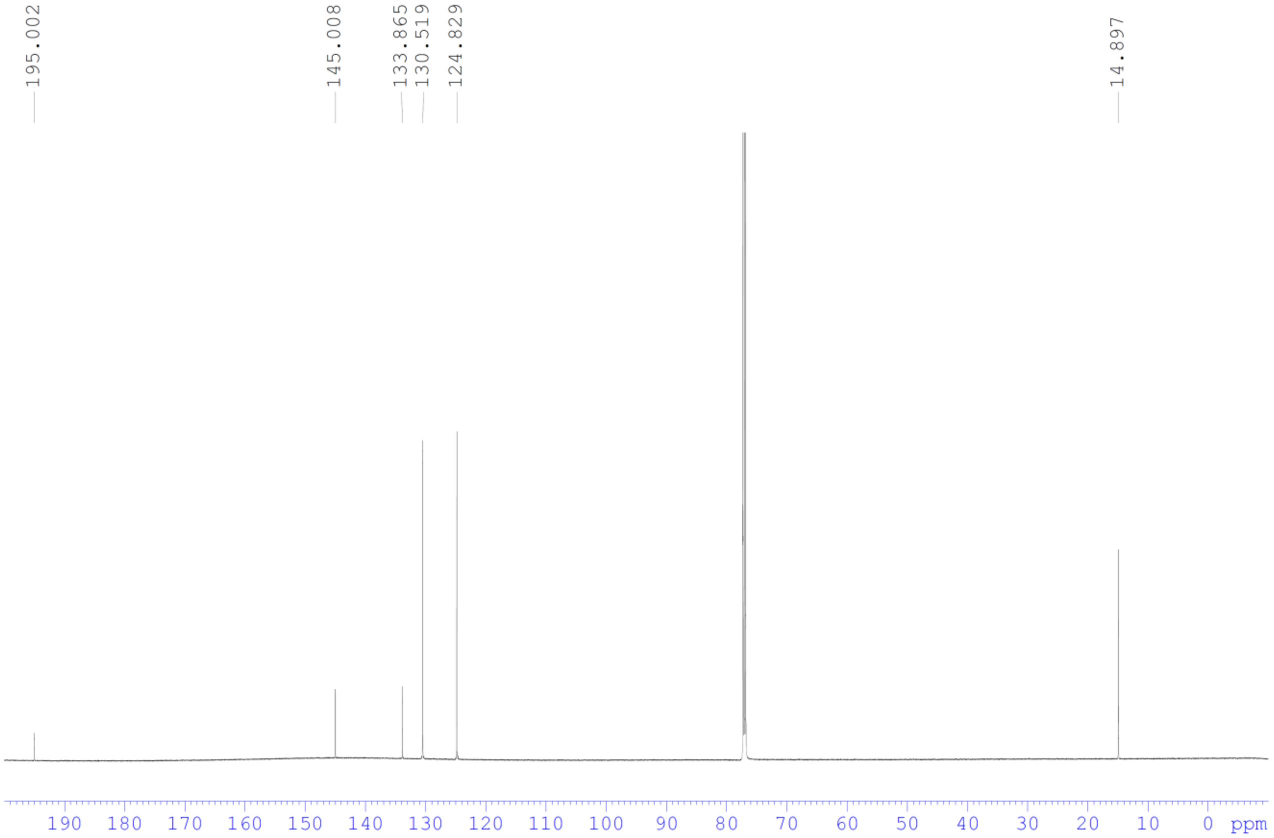

^{1}H NMR (600 MHz) and ^{13}C NMR (151 MHz) spectra of **8** ($CDCl_3$)

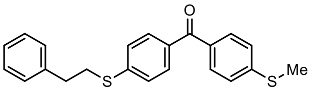

8

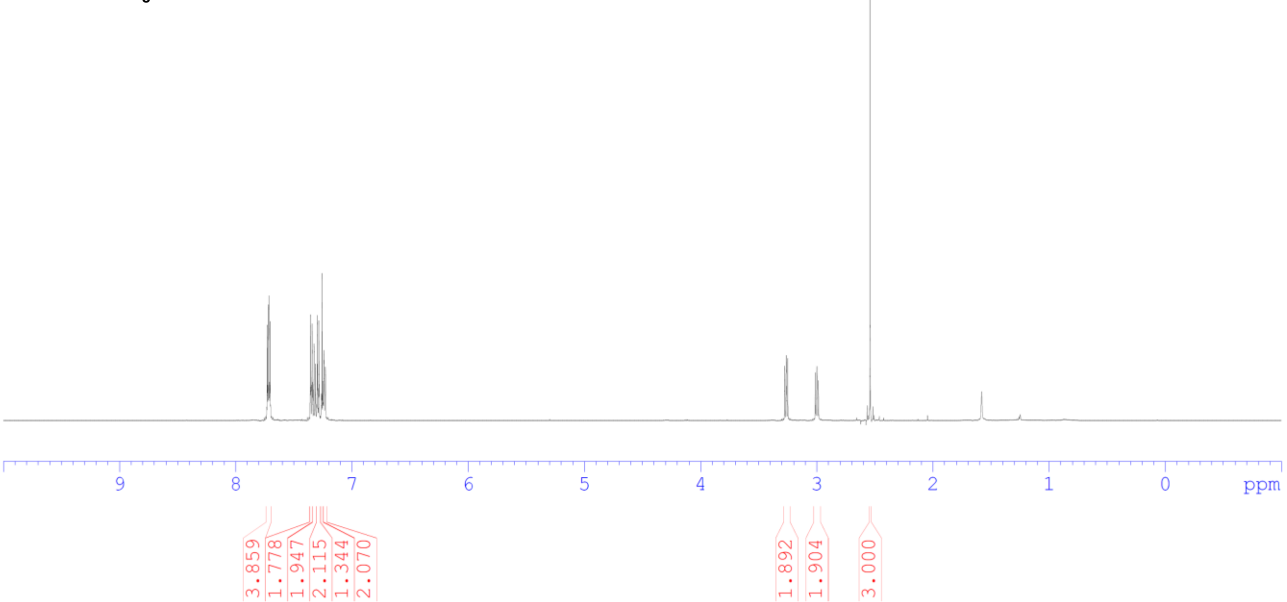

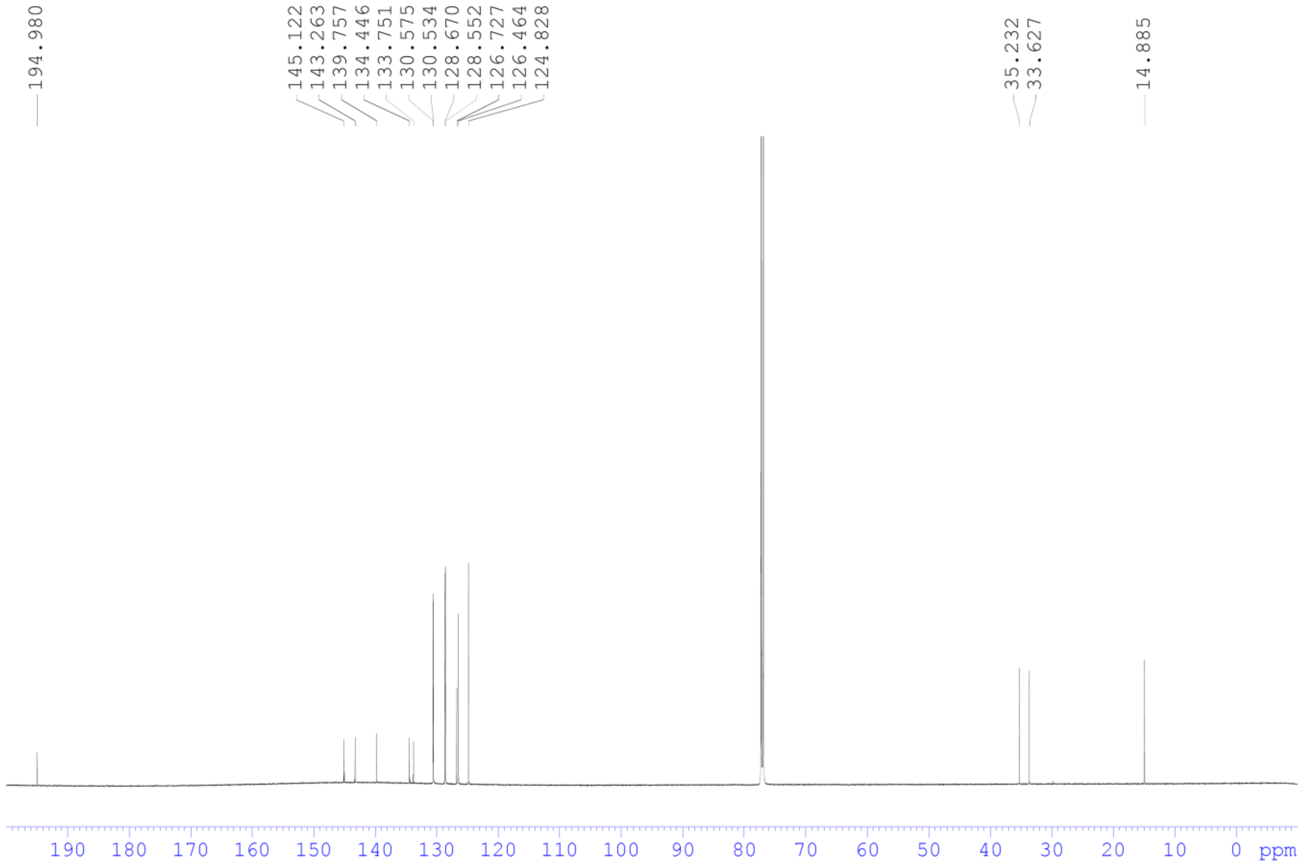

^{1}H NMR (600 MHz) and ^{13}C NMR (151 MHz) spectra of **9** ($CDCl_3$)

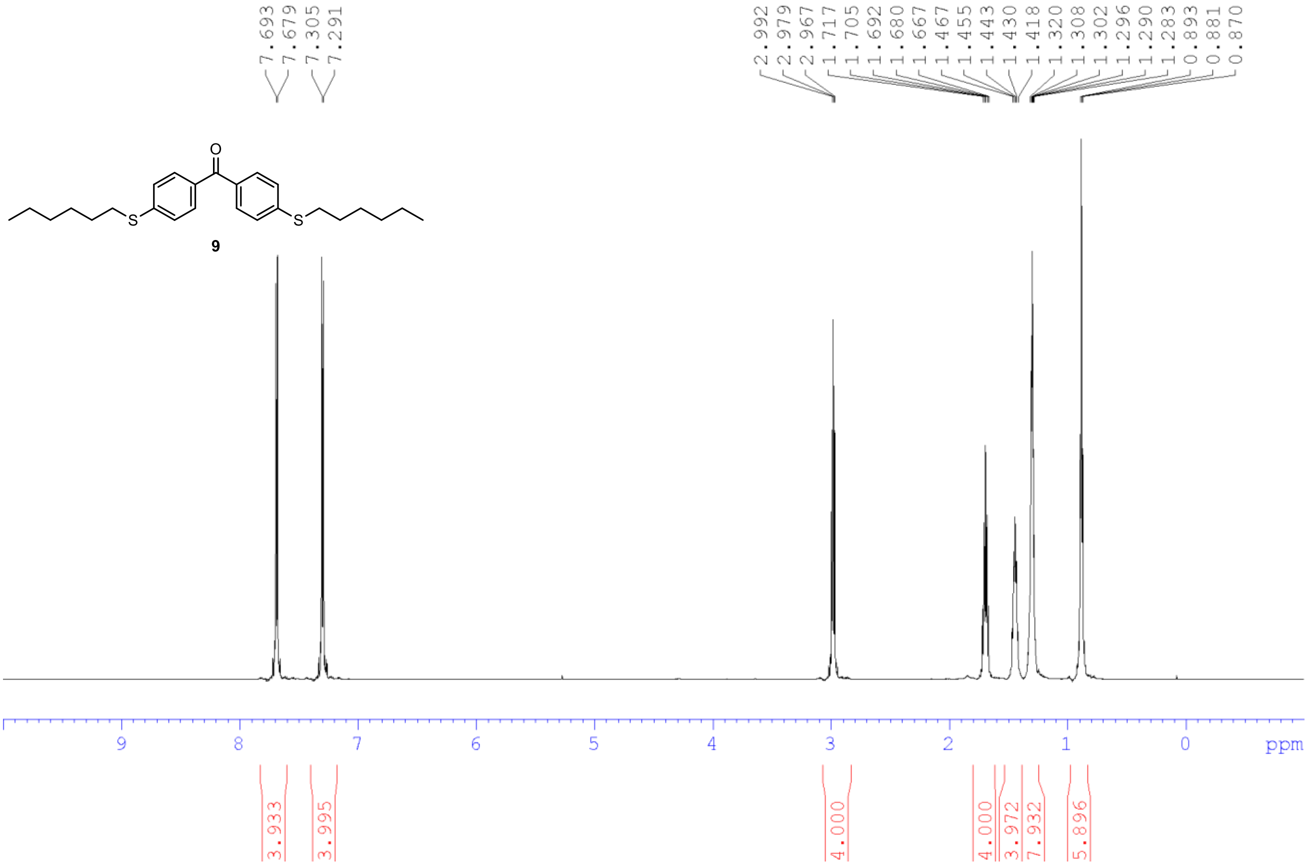

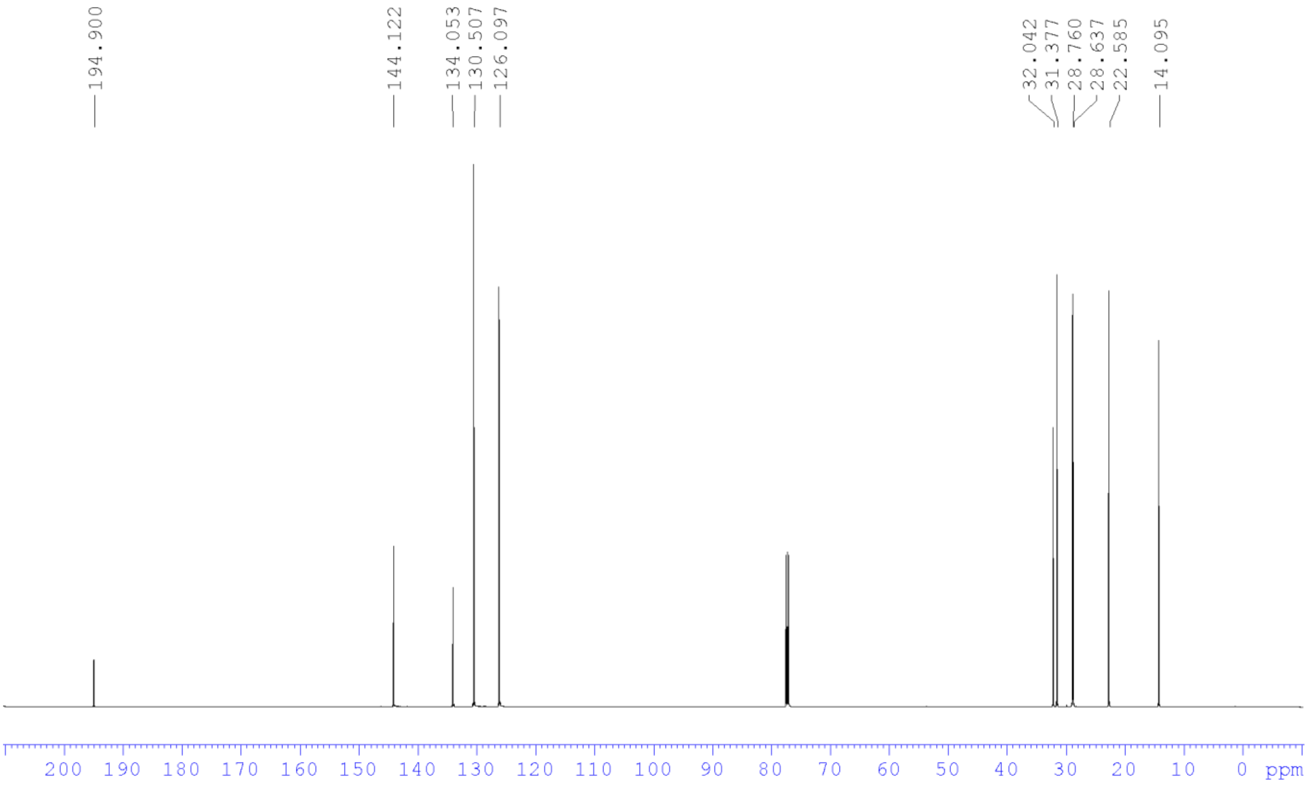

^{1}H NMR (600 MHz) and ^{13}C NMR (151 MHz) spectra of **10** ($CDCl_3$)

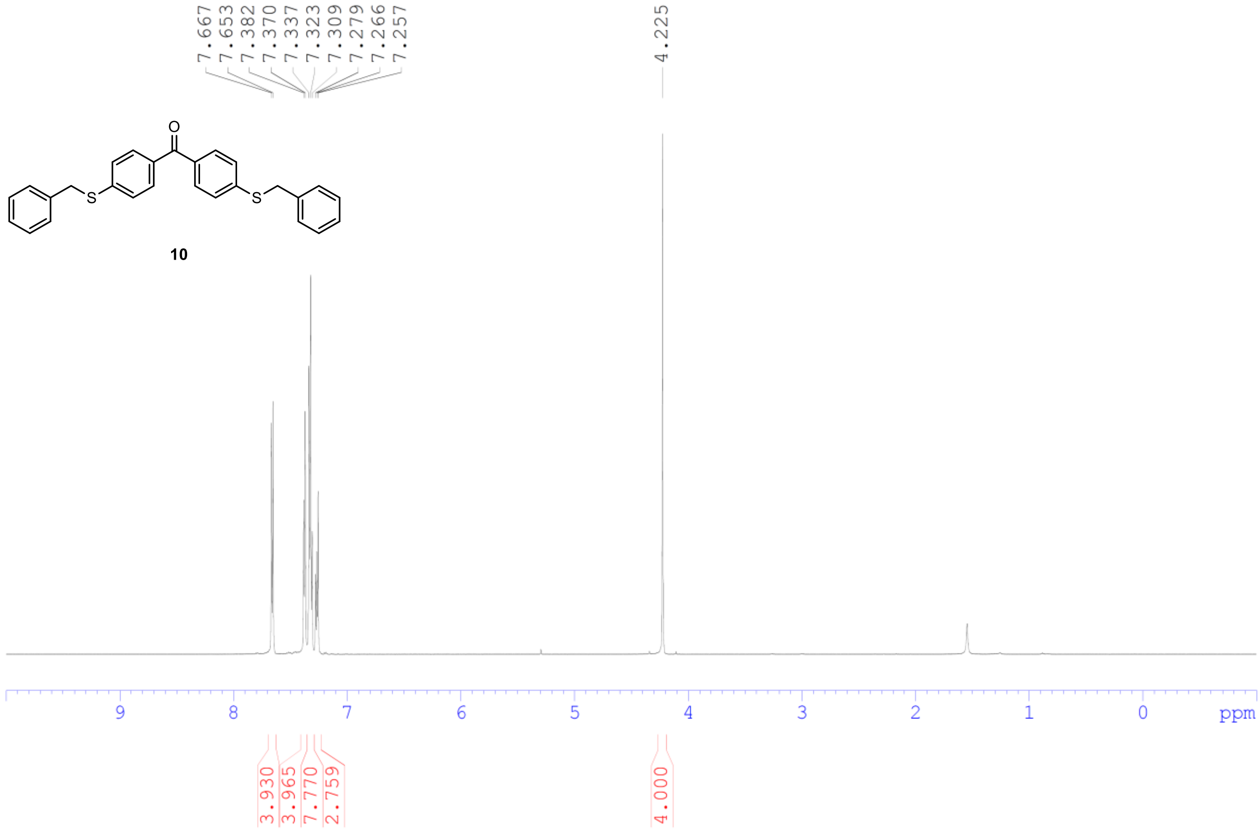

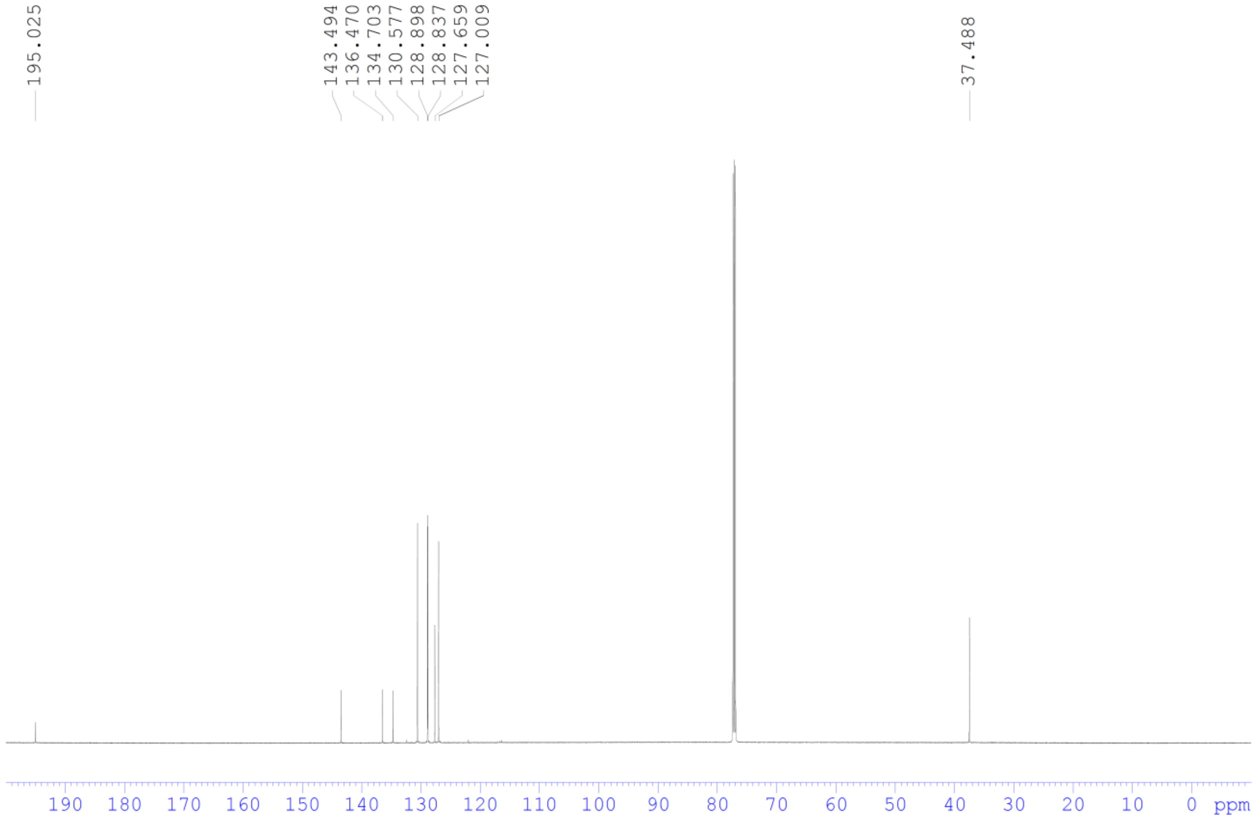

^{1}H NMR (600 MHz) and ^{13}C NMR (151 MHz) spectra of **11** ($CDCl_3$)

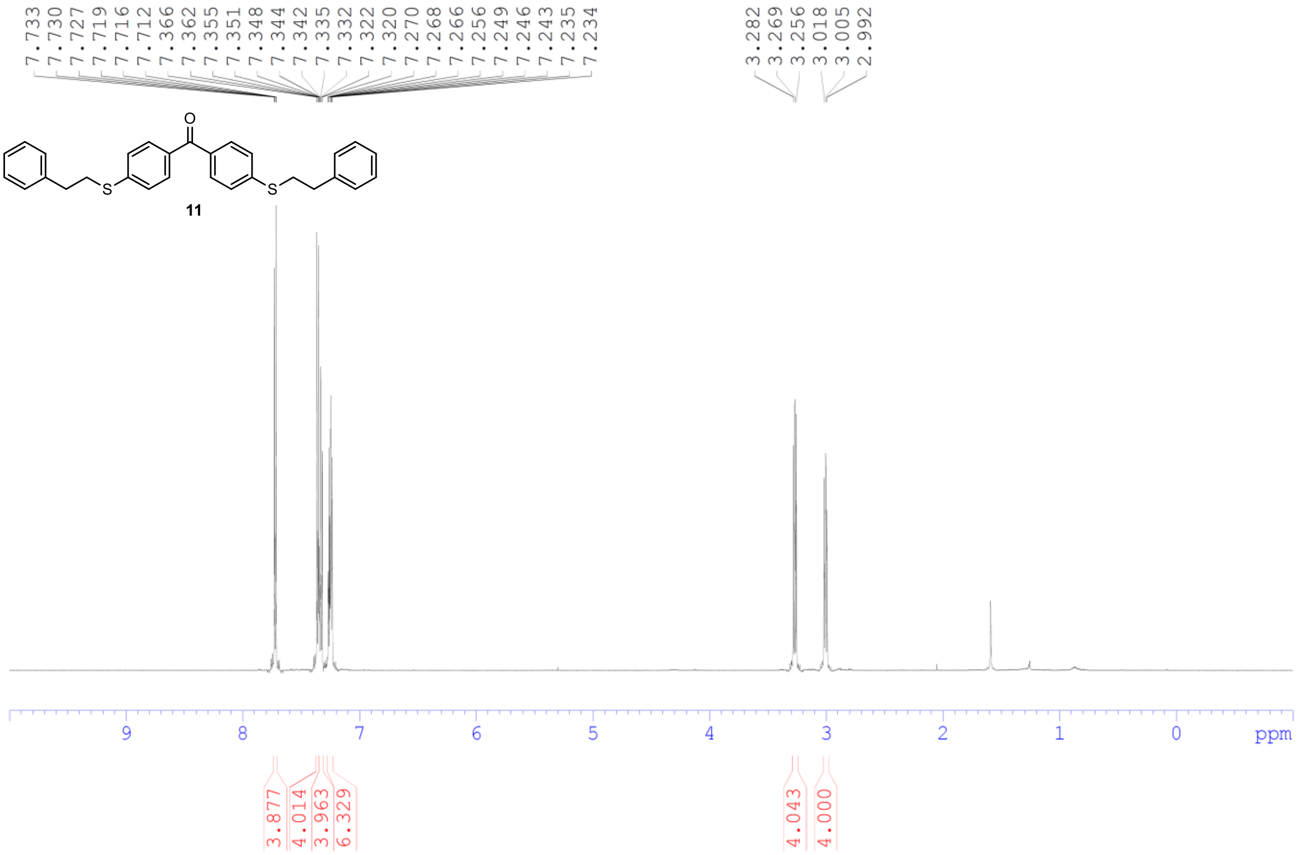

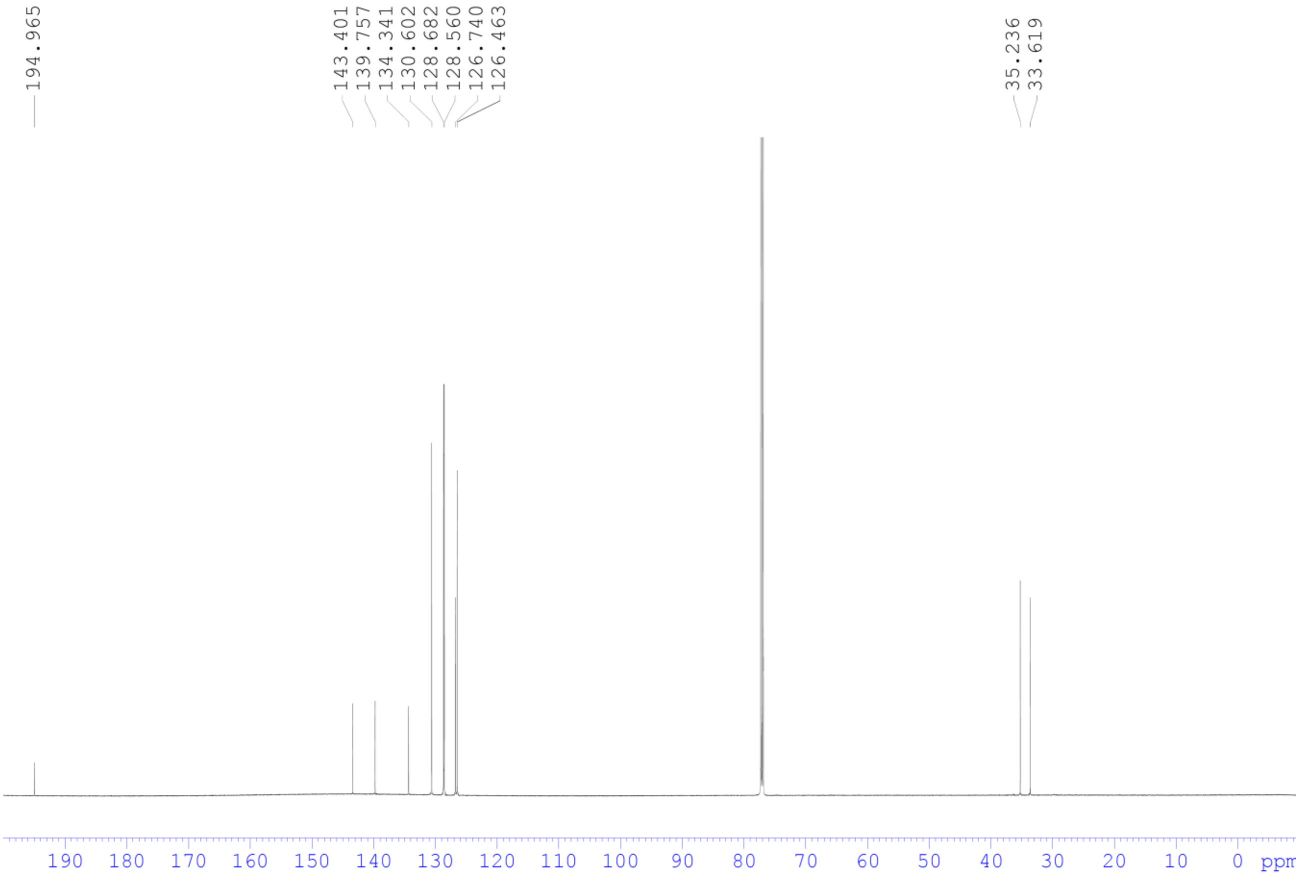

^{1}H NMR (600 MHz) and ^{13}C NMR (151 MHz) spectra of **12** ($CDCl_3$)

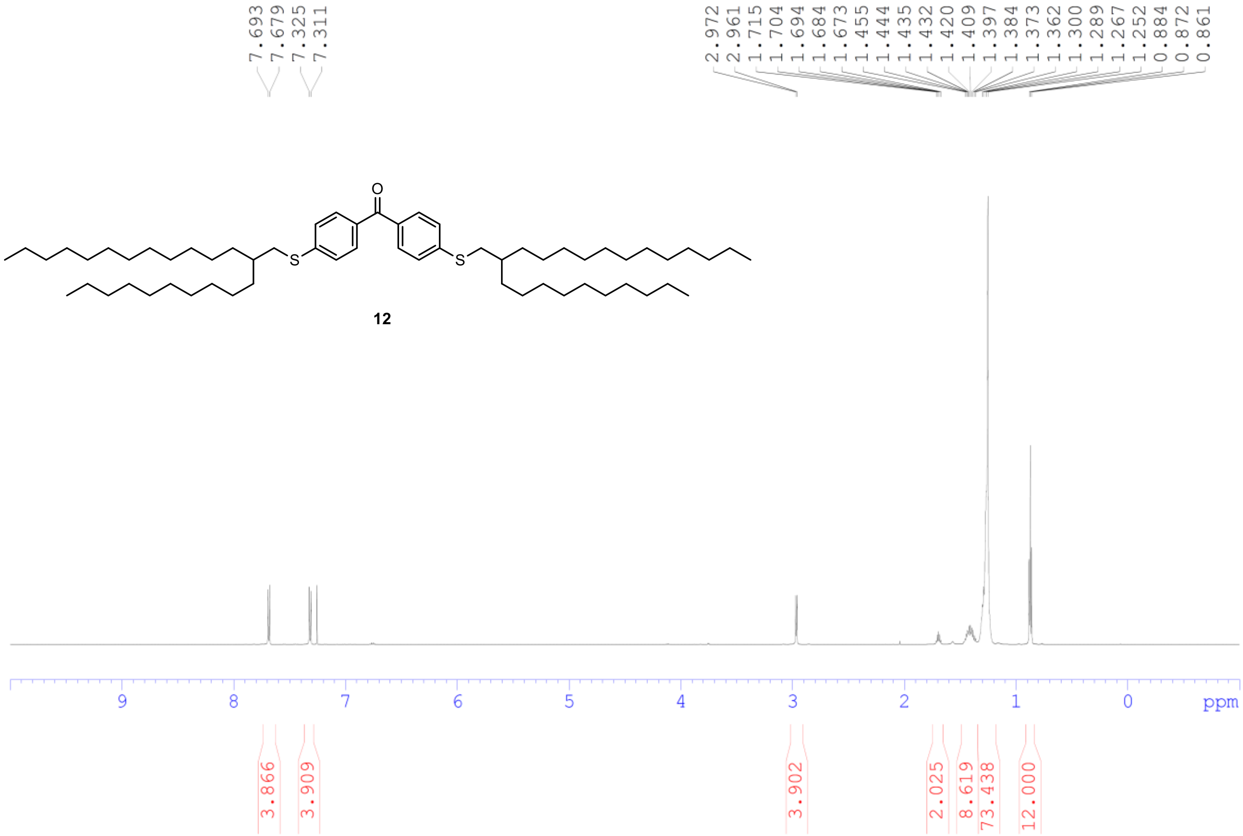

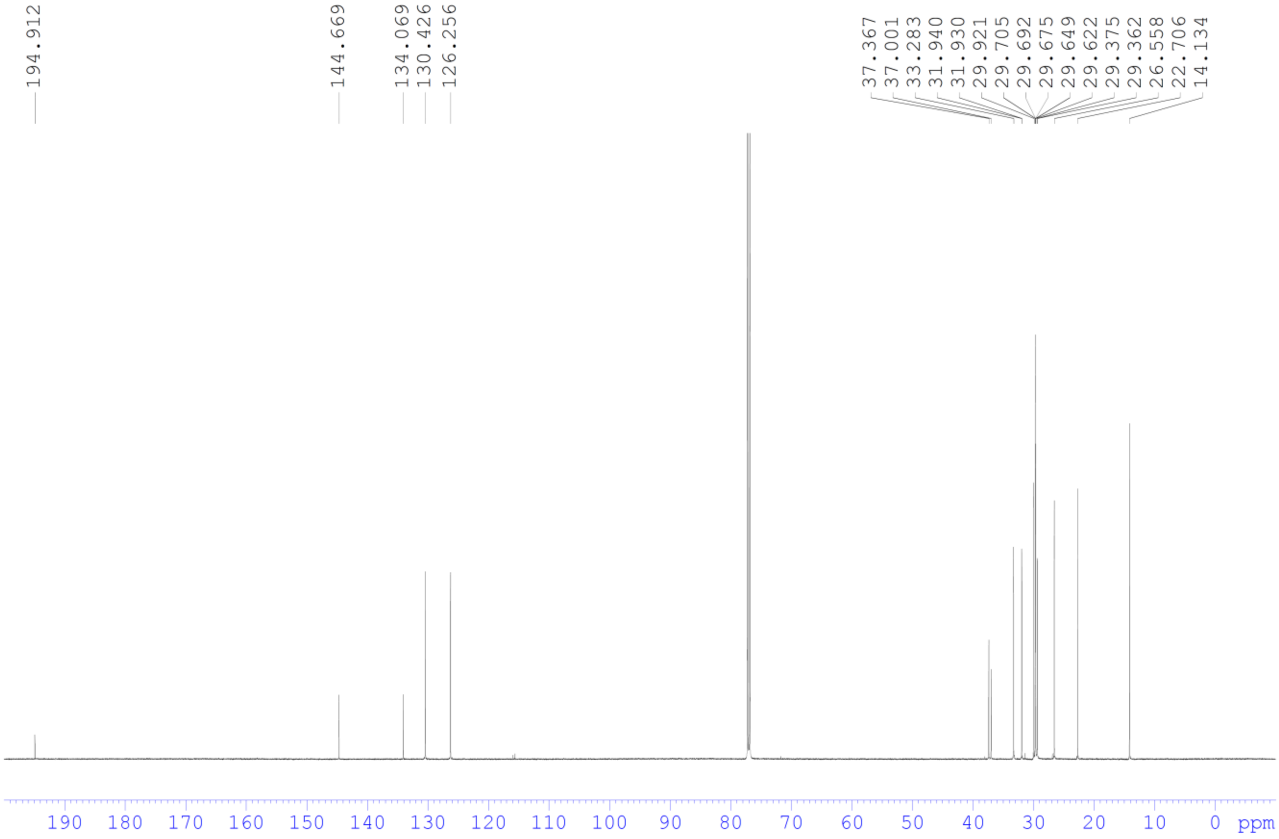

^{1}H NMR (600 MHz) and ^{13}C NMR (151 MHz) spectra of **13** ($CDCl_3$)

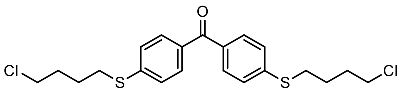

13

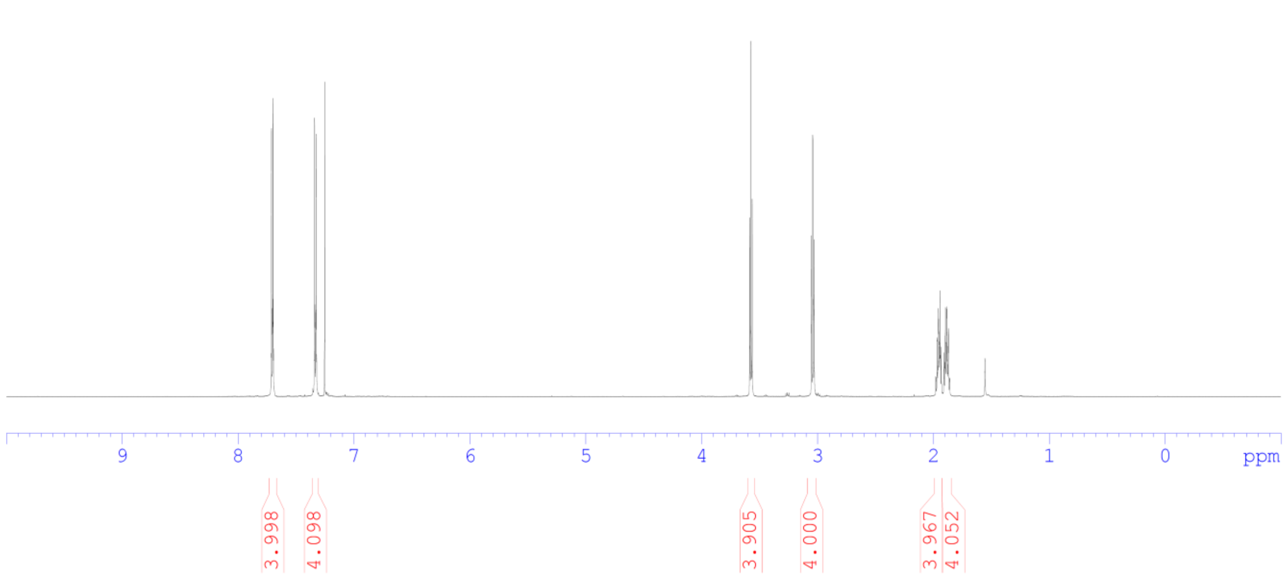

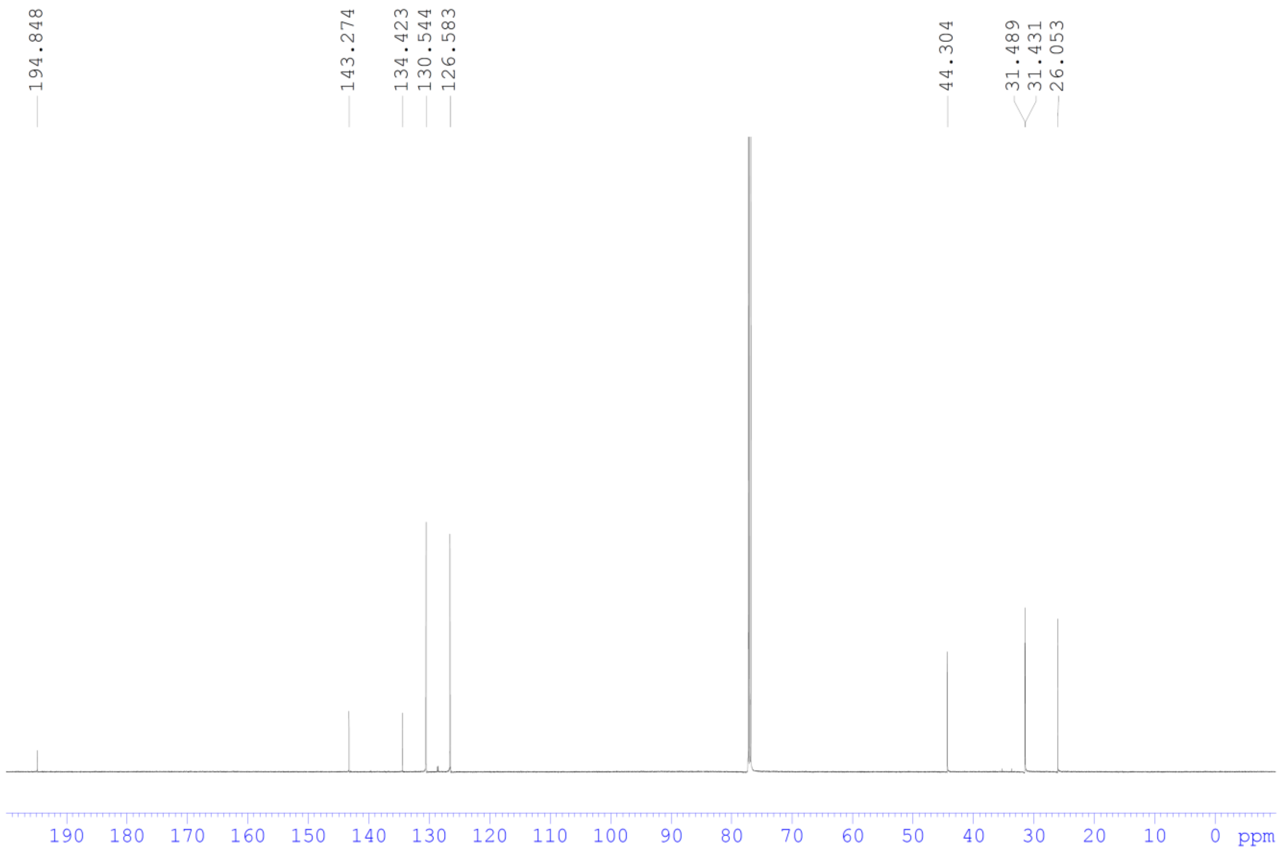

^{1}H NMR (600 MHz) and ^{13}C NMR (151 MHz) spectra of **14** ($CDCl_3$)

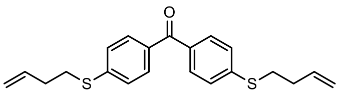

14

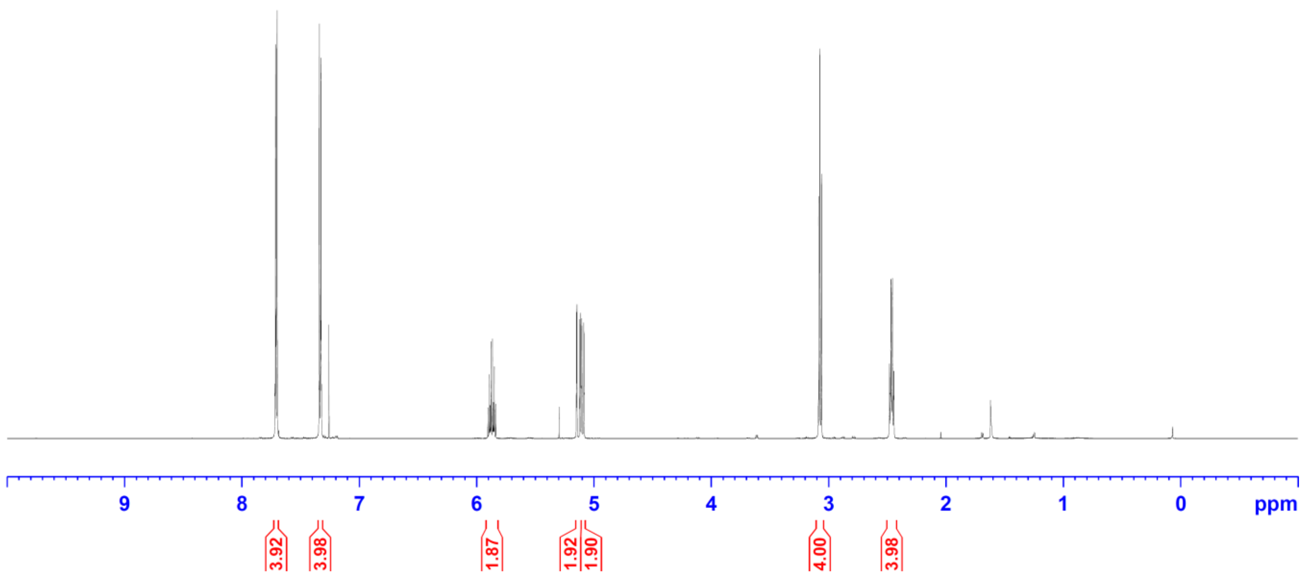

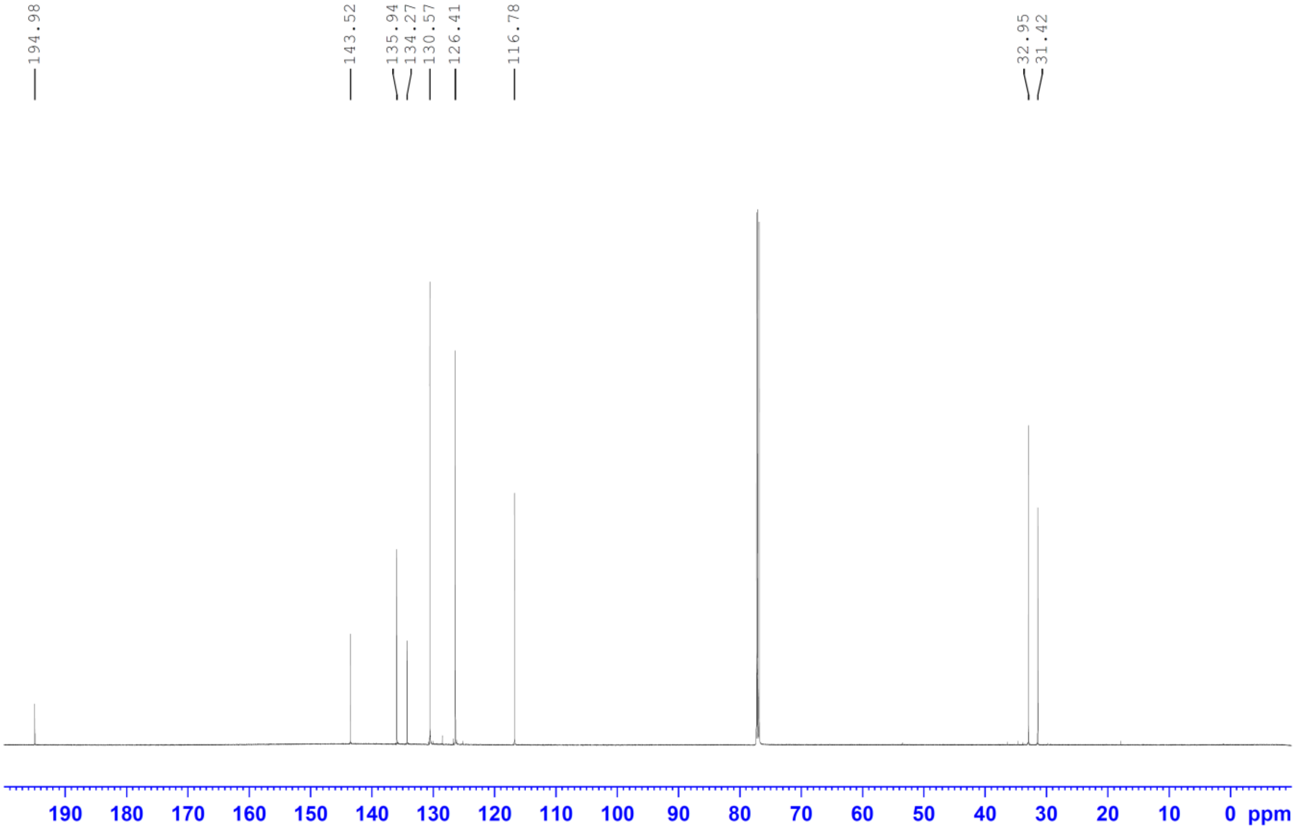

^{1}H NMR (600 MHz) and ^{13}C NMR (151 MHz) spectra of **15** ($CDCl_3$)

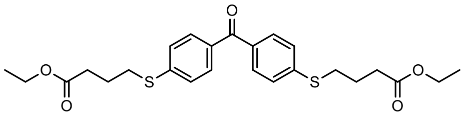

15

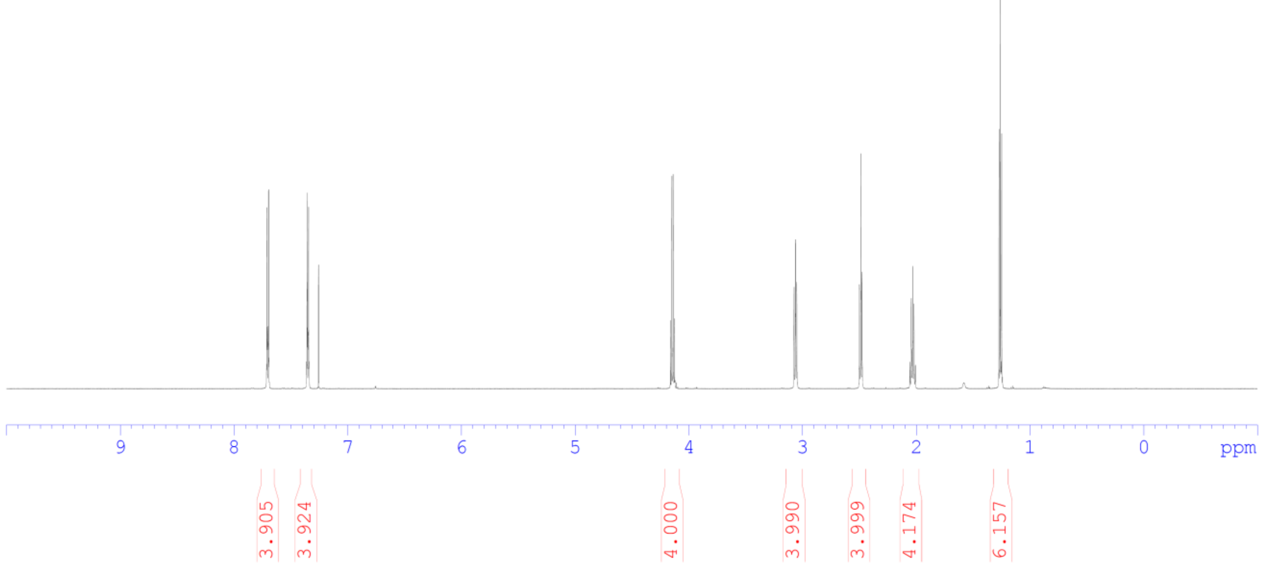

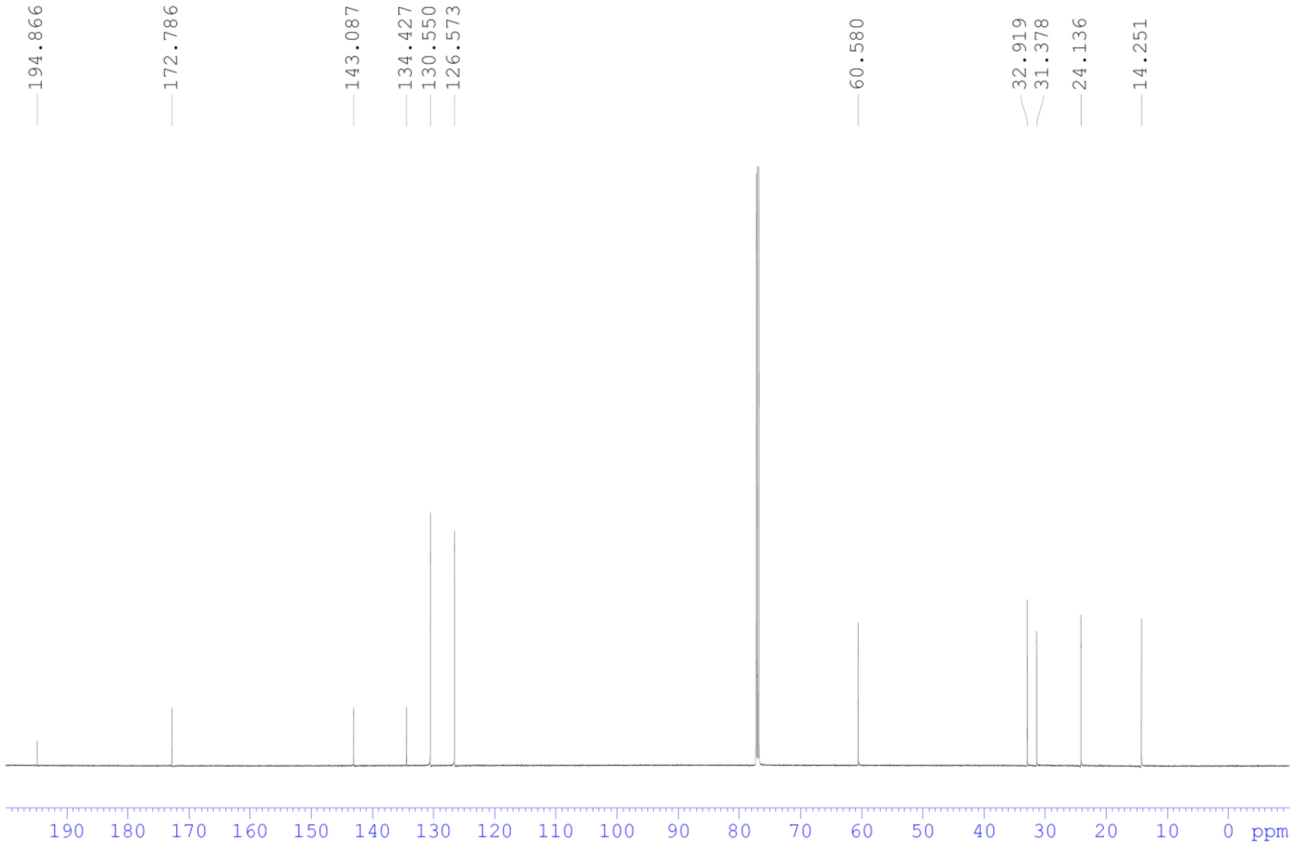

^{1}H NMR (600 MHz) and ^{13}C NMR (151 MHz) spectra of **16** ($CDCl_3$)

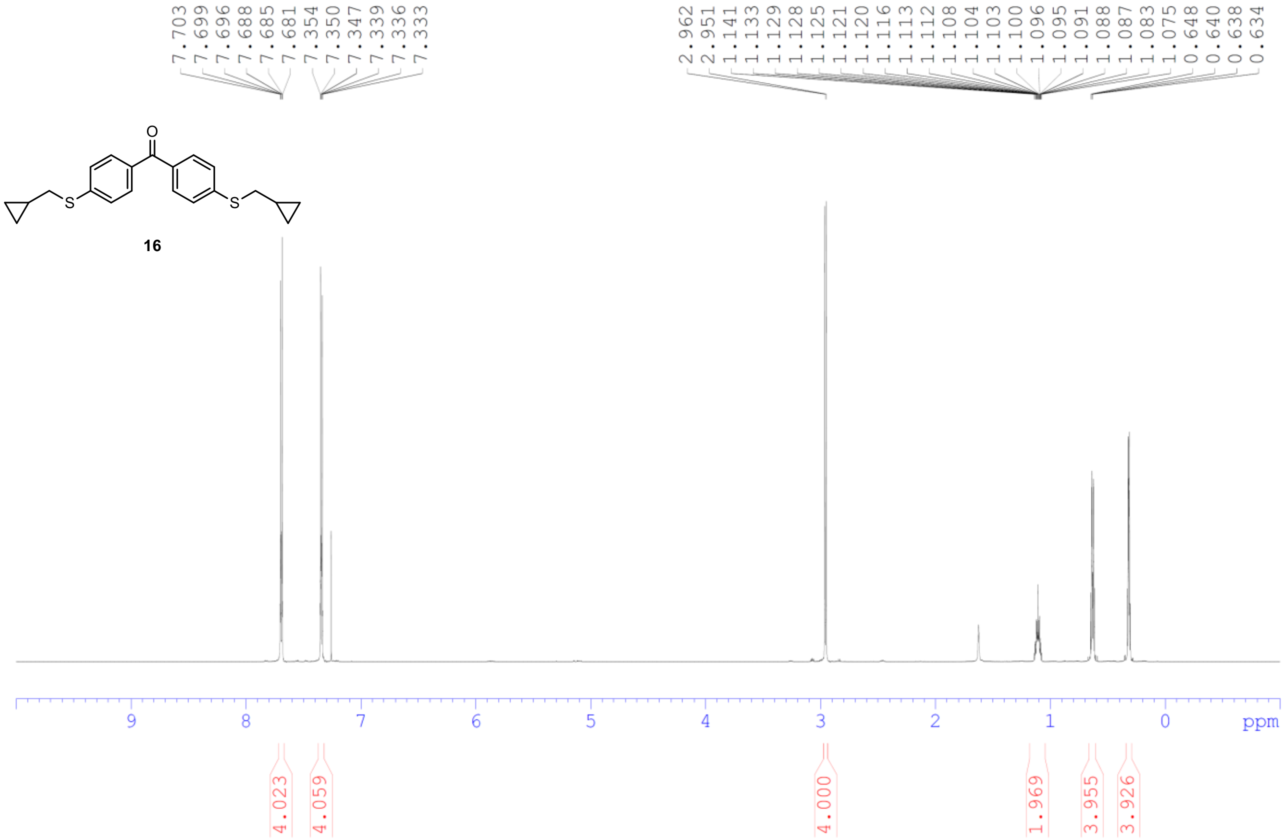

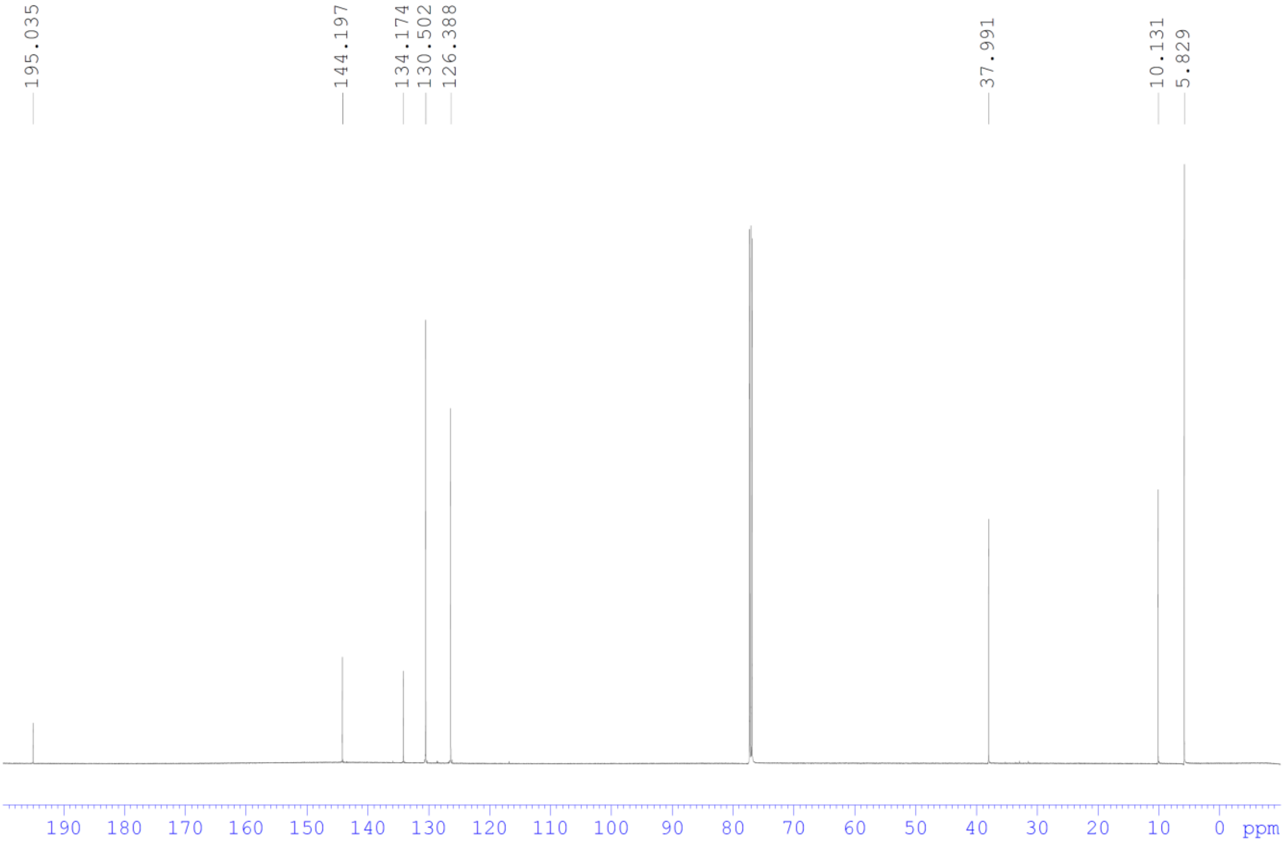

^{1}H NMR (600 MHz) and ^{13}C NMR (151 MHz) spectra of **17** ($CDCl_3$/CD_3OD (1:9))

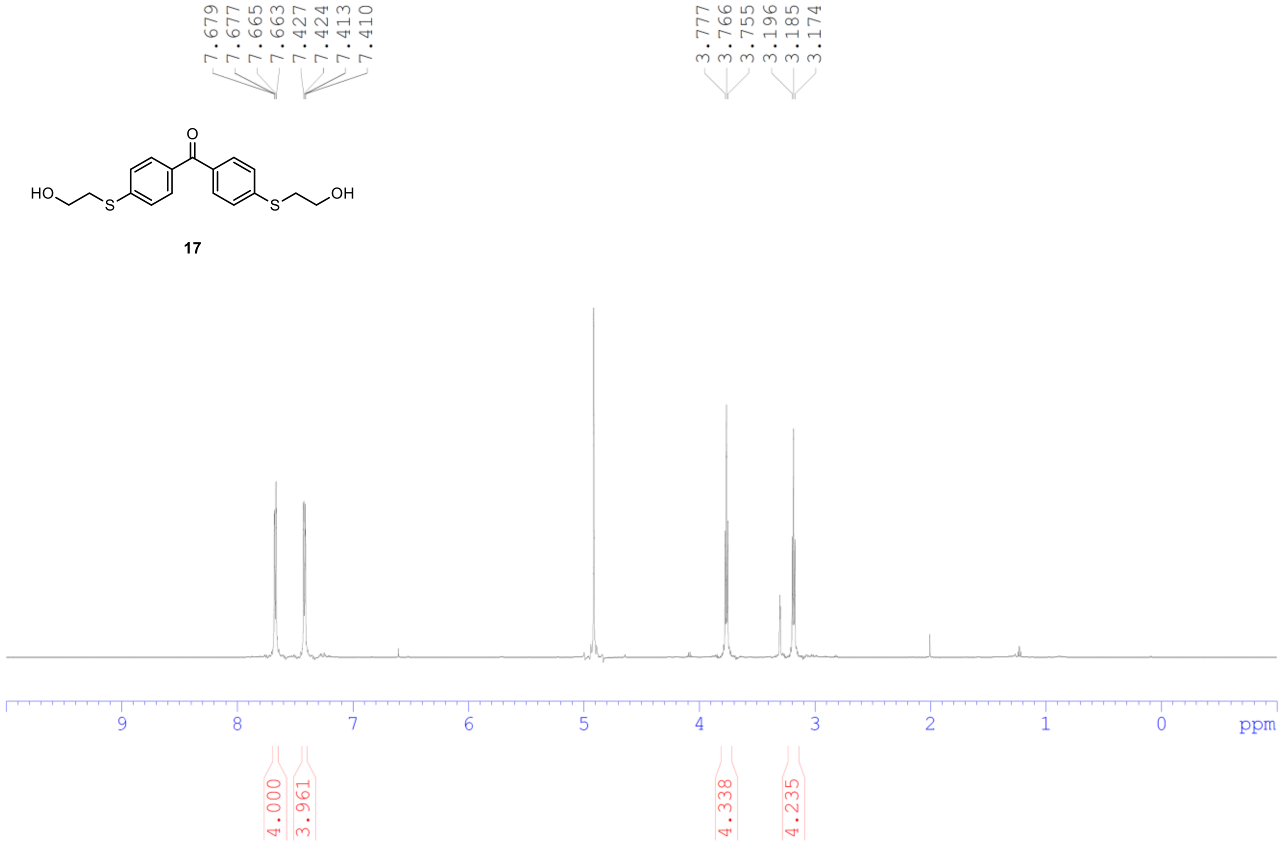

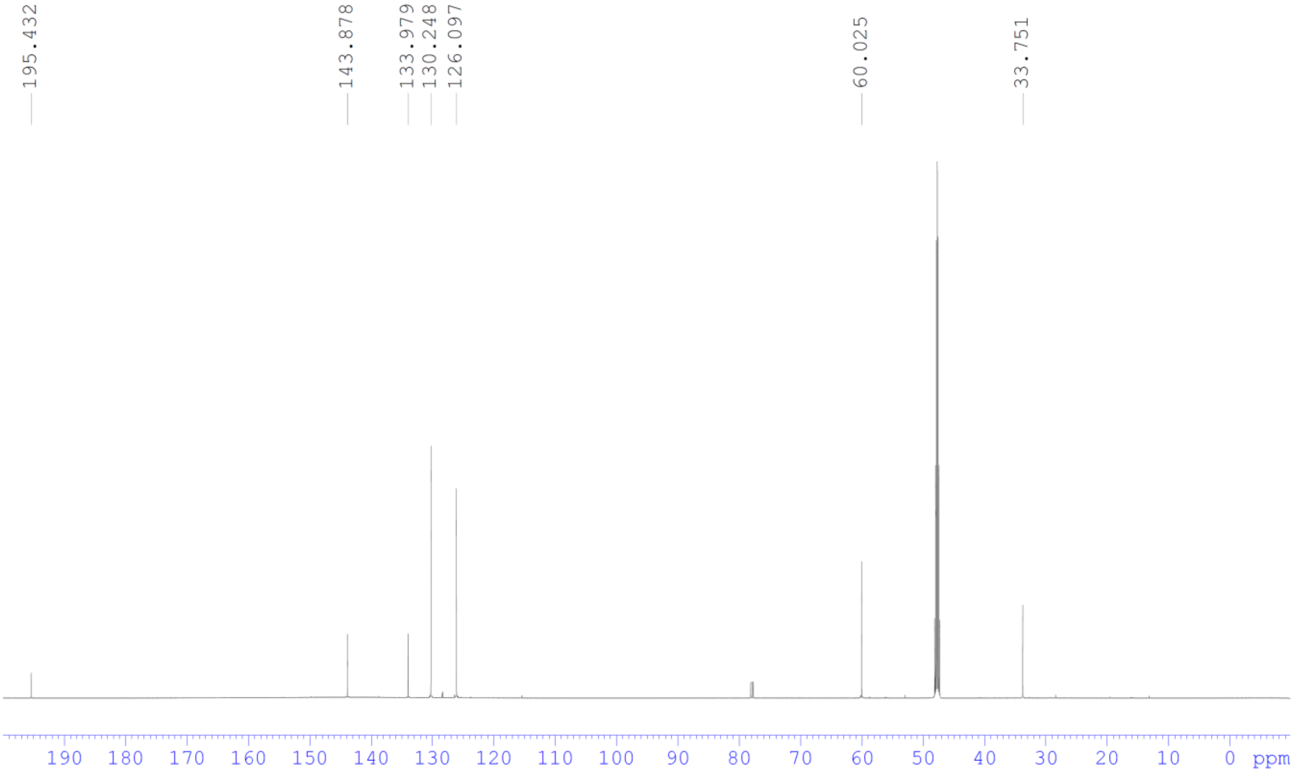

^{1}H NMR (600 MHz) and ^{13}C NMR (151 MHz) spectra of **18** ($CDCl_3$)

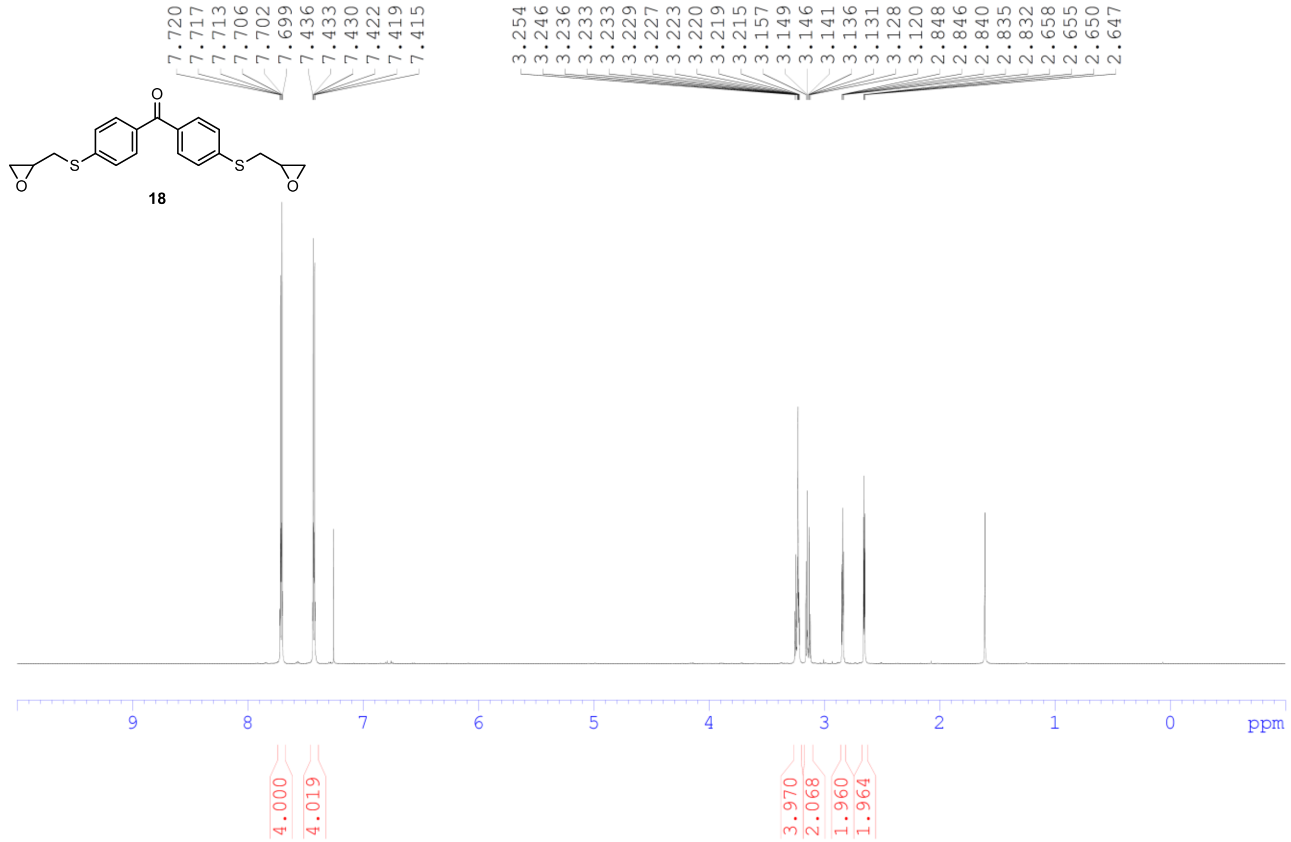

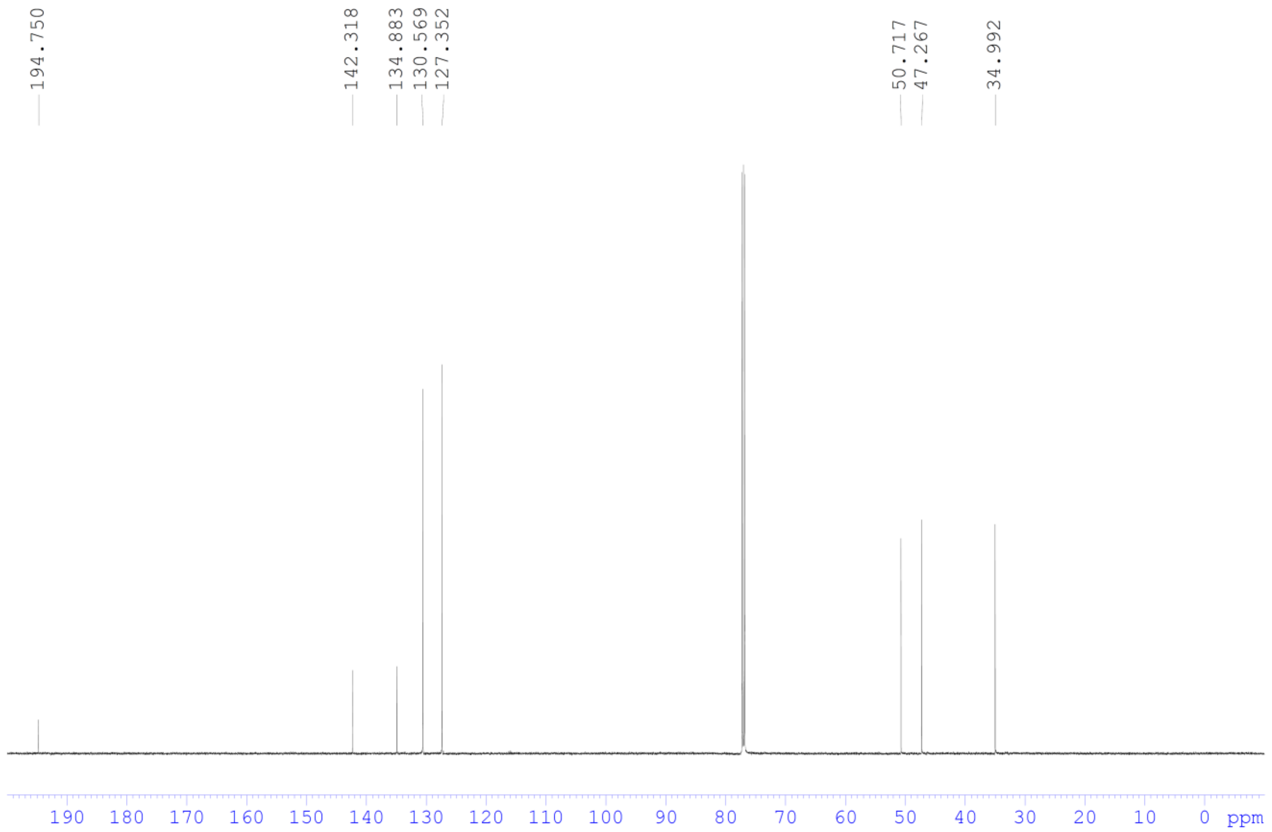

^{1}H NMR (600 MHz) and ^{13}C NMR (151 MHz) spectra of **19** ($CDCl_3$)

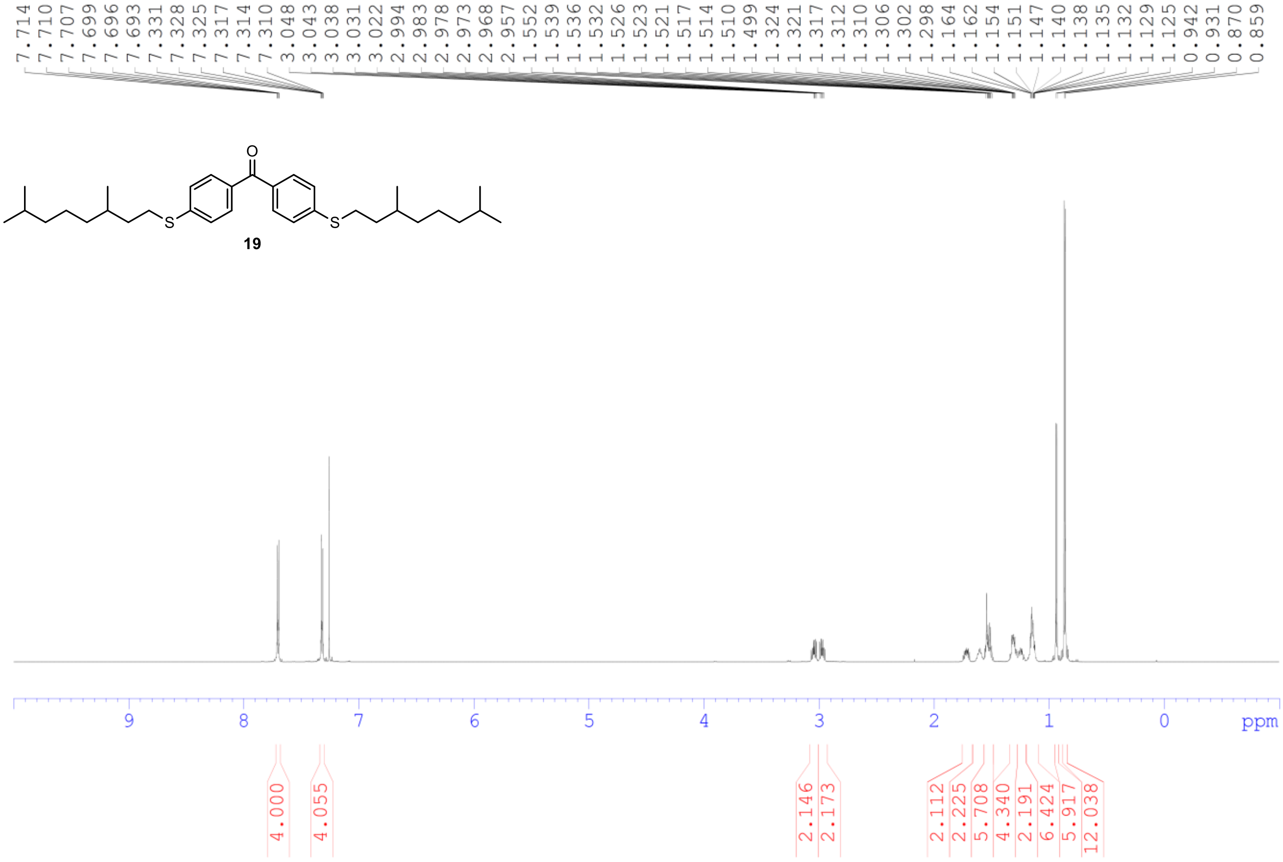

19

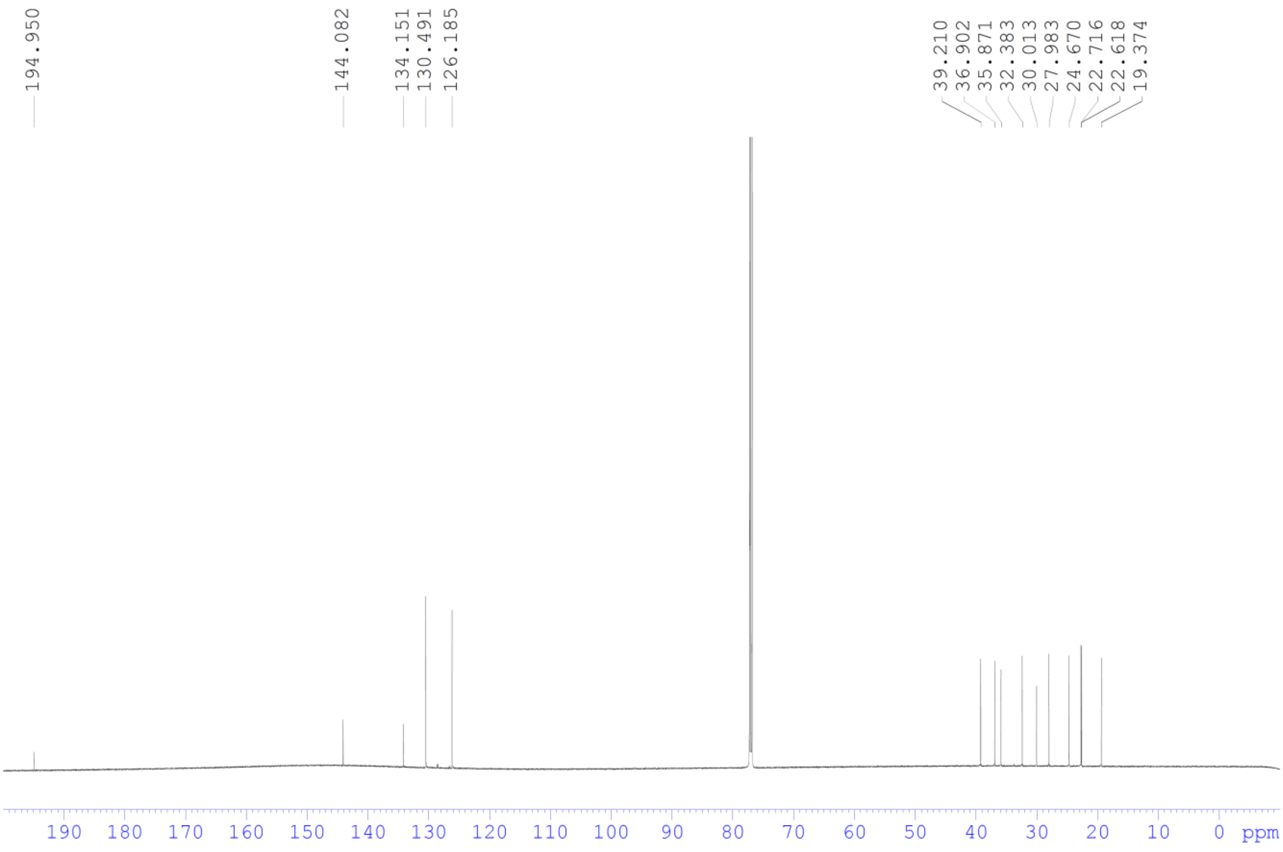

^{1}H NMR (600 MHz) and ^{13}C NMR (151 MHz) spectra of **20** ($CDCl_3$)

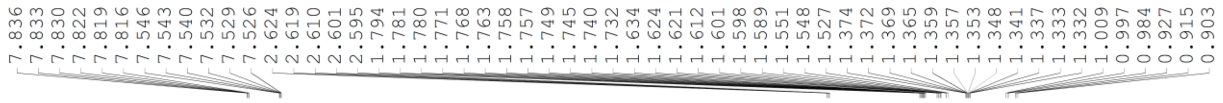

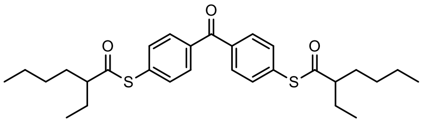

20

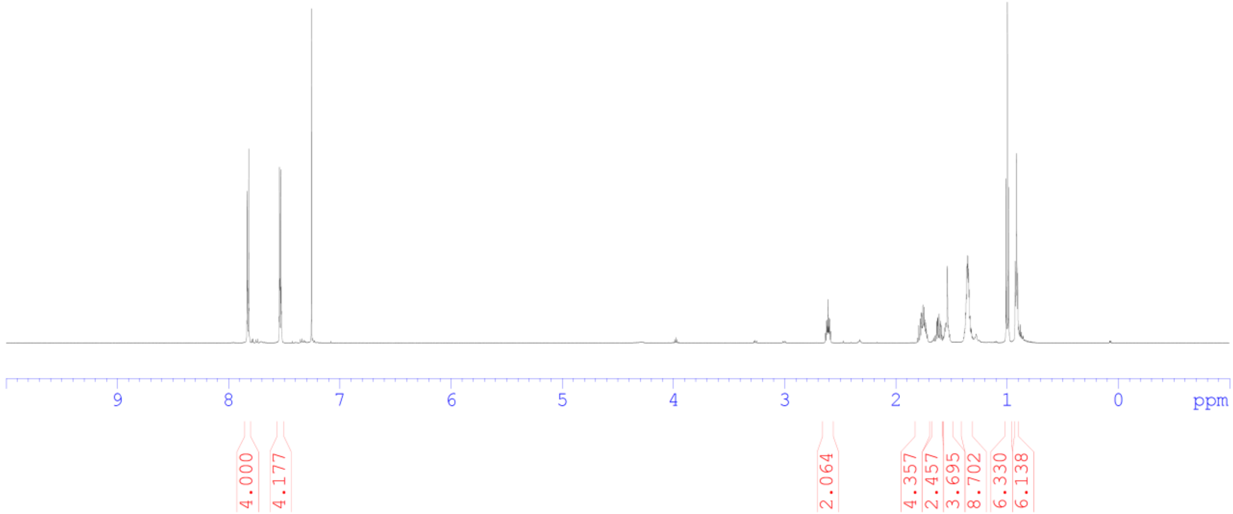

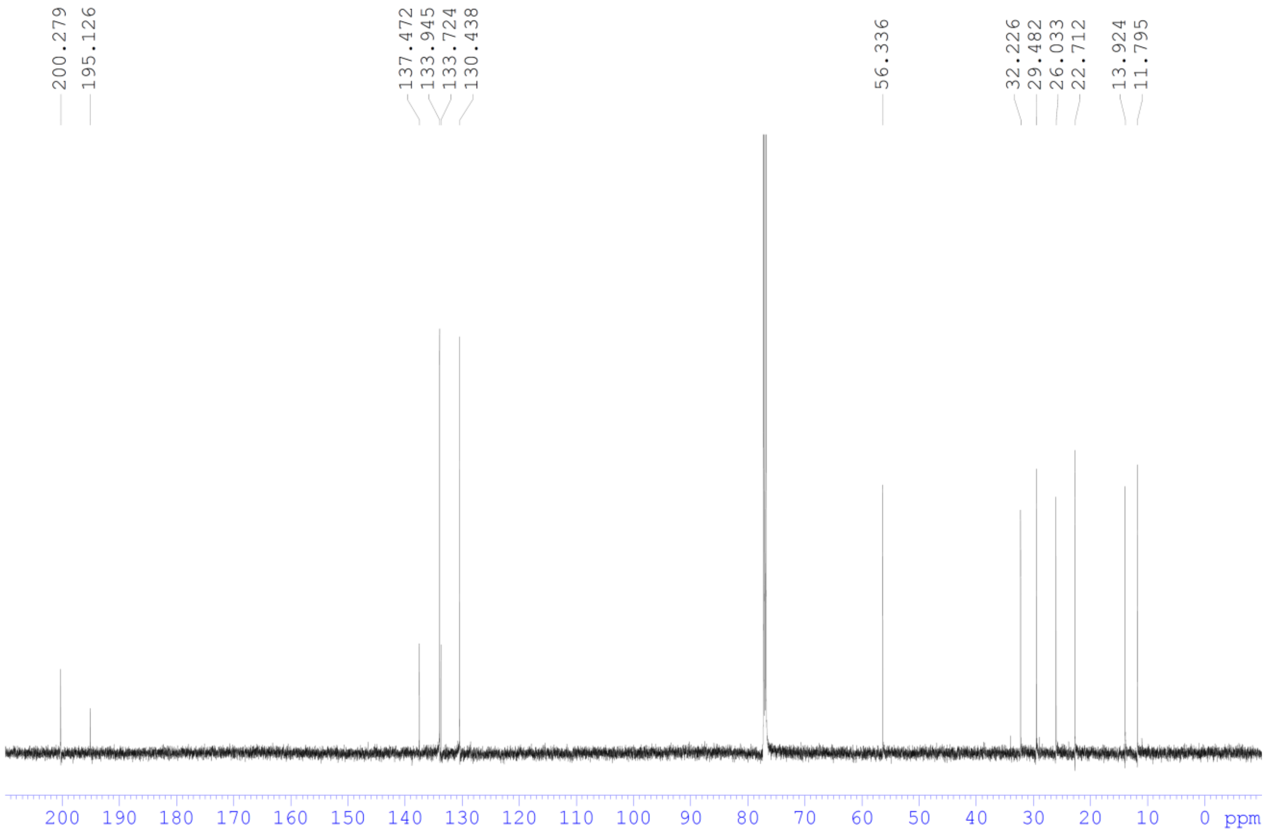

^{1}H NMR (600 MHz) and ^{13}C NMR (151 MHz) spectra of **21** ($CDCl_3$)

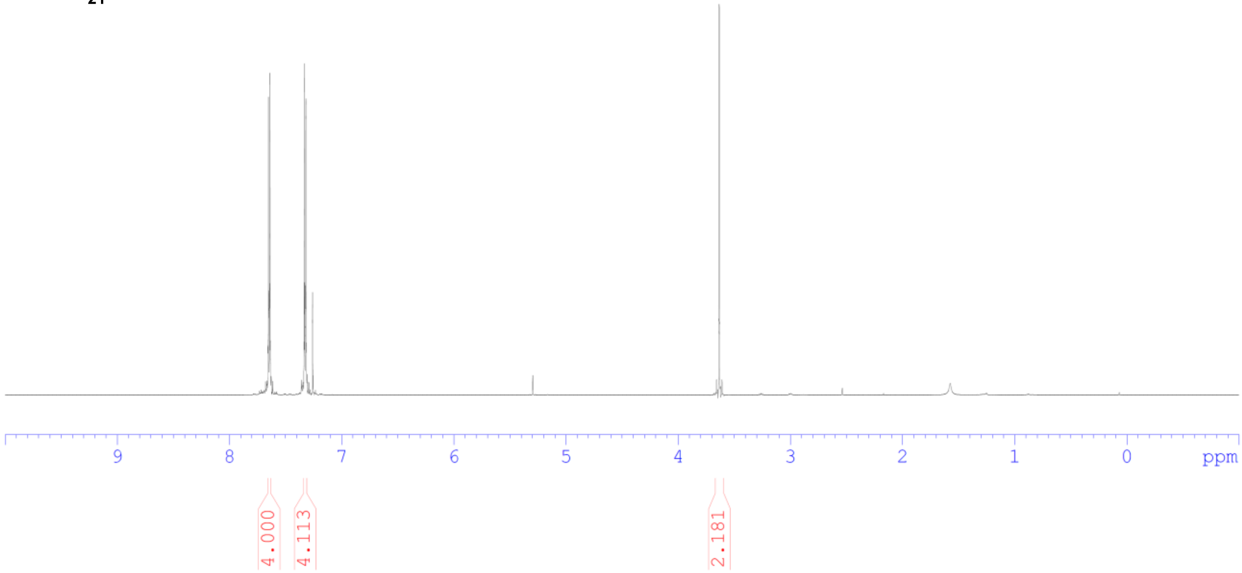

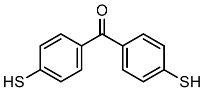

21

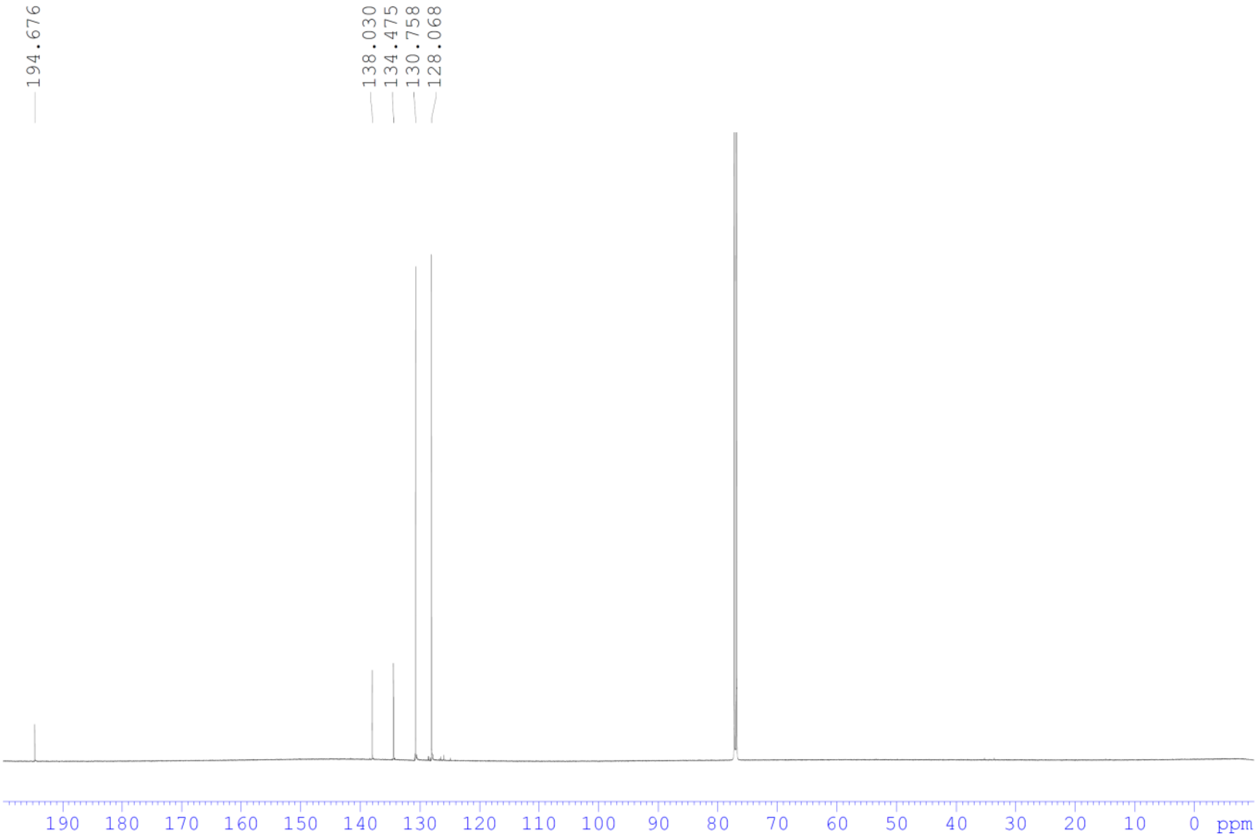

^{1}H NMR (600 MHz) and ^{13}C NMR (151 MHz) spectra of **22** ($CDCl_3$)

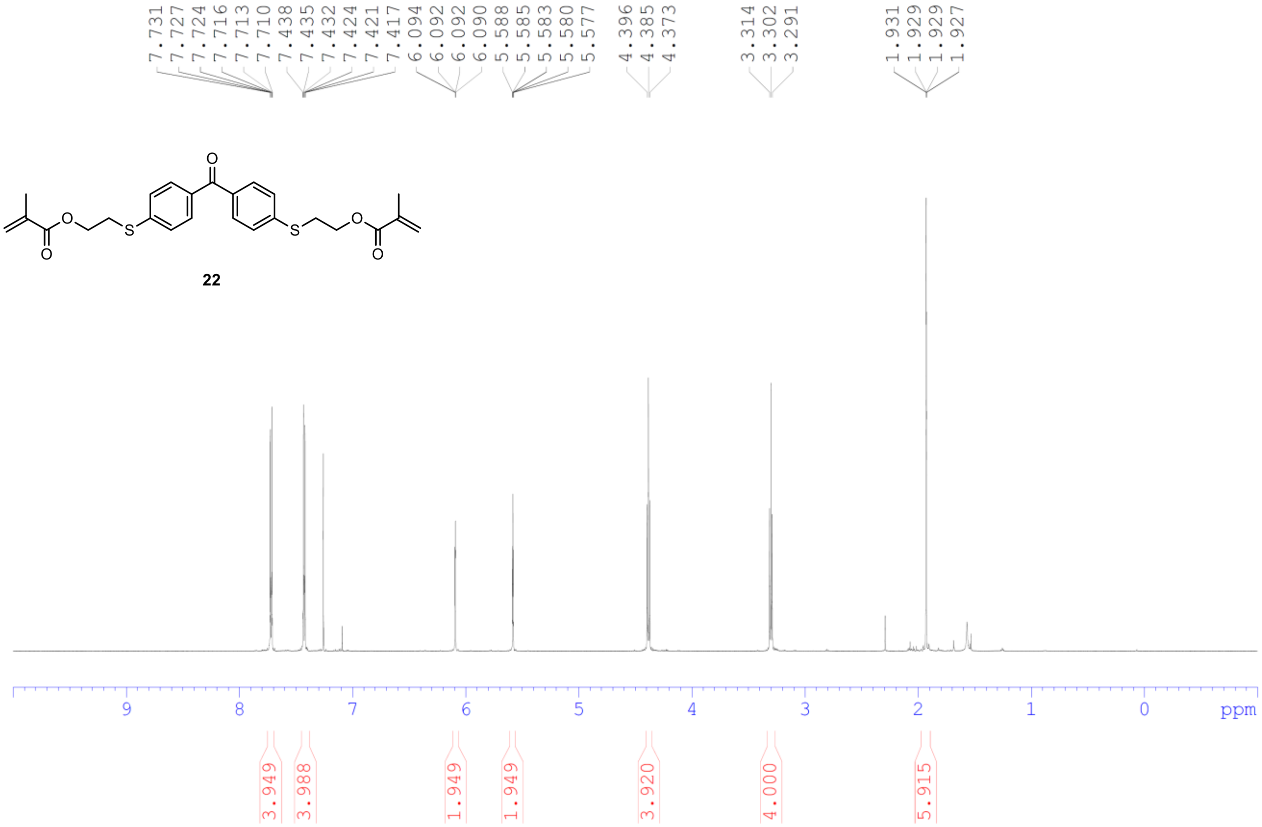

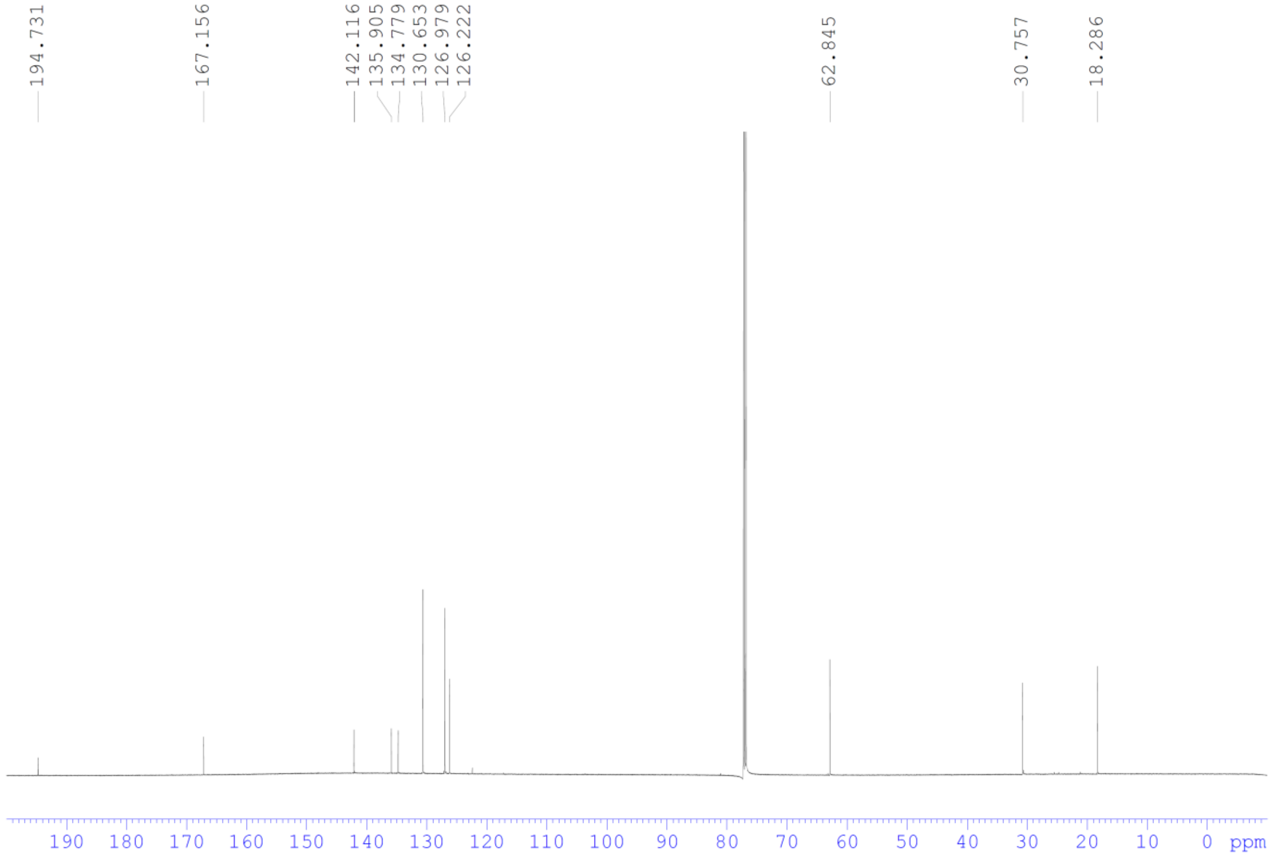

^{1}H NMR (600 MHz) and ^{13}C NMR (151 MHz) spectra of **24** (acetone-d_6)

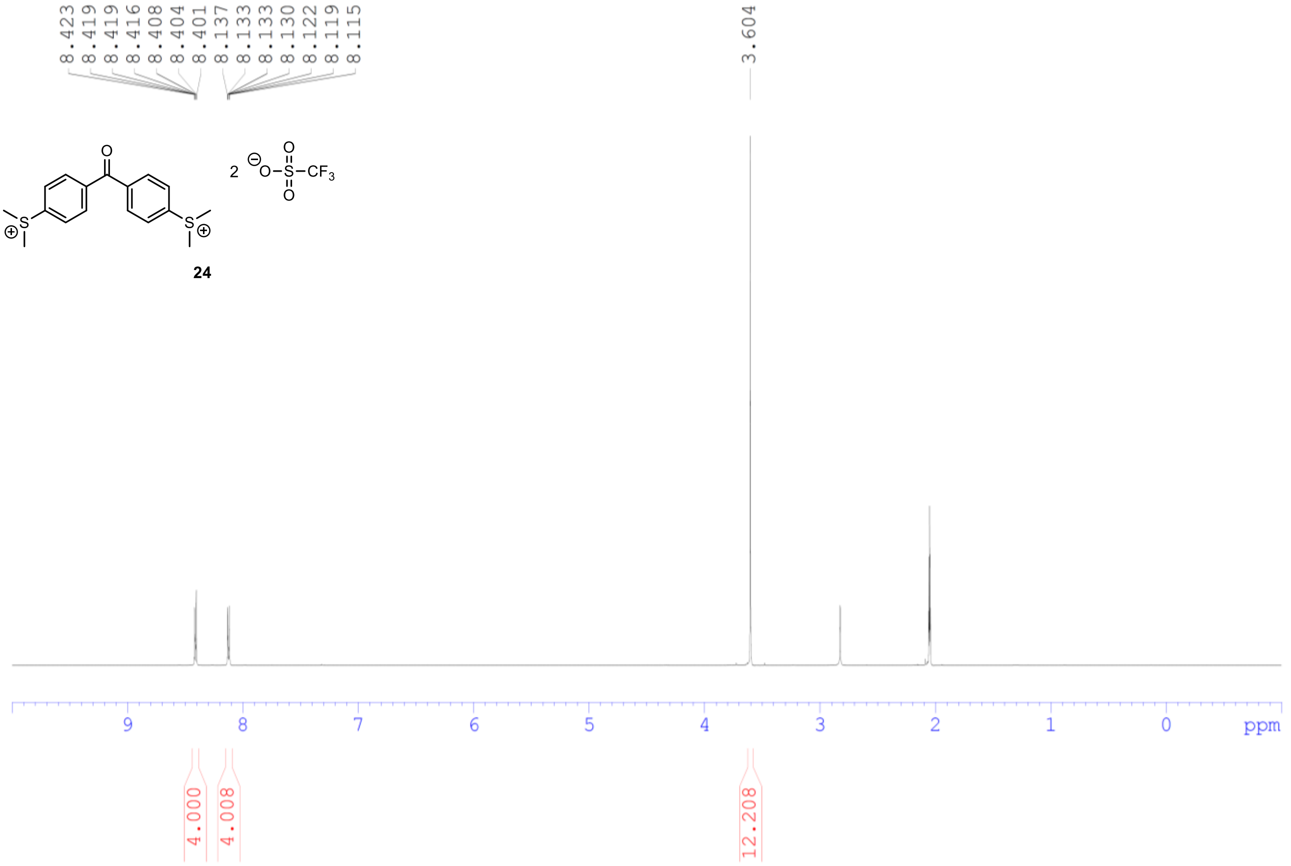

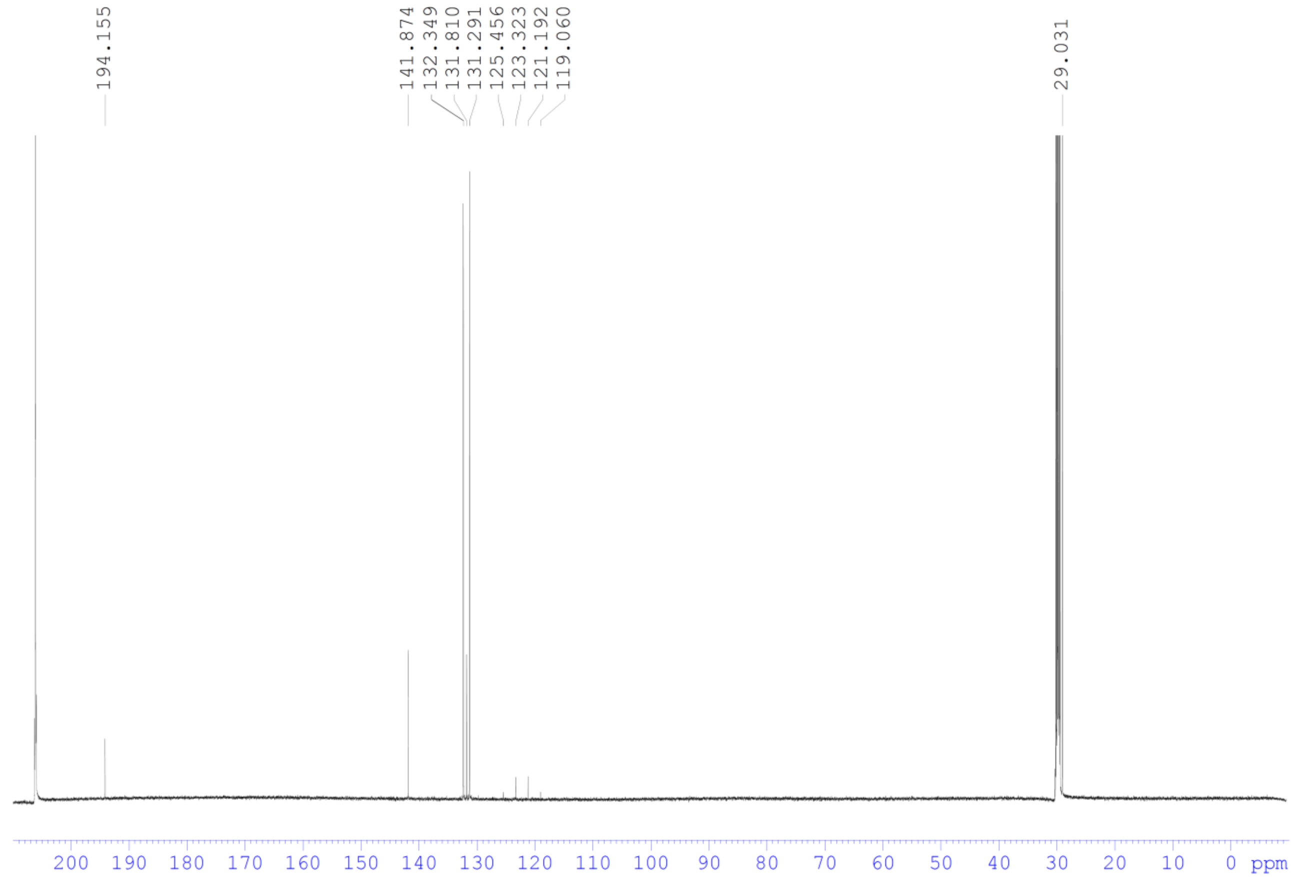

^{1}H NMR (600 MHz) and ^{13}C NMR (151 MHz) spectra of **25** ($CDCl_3$)

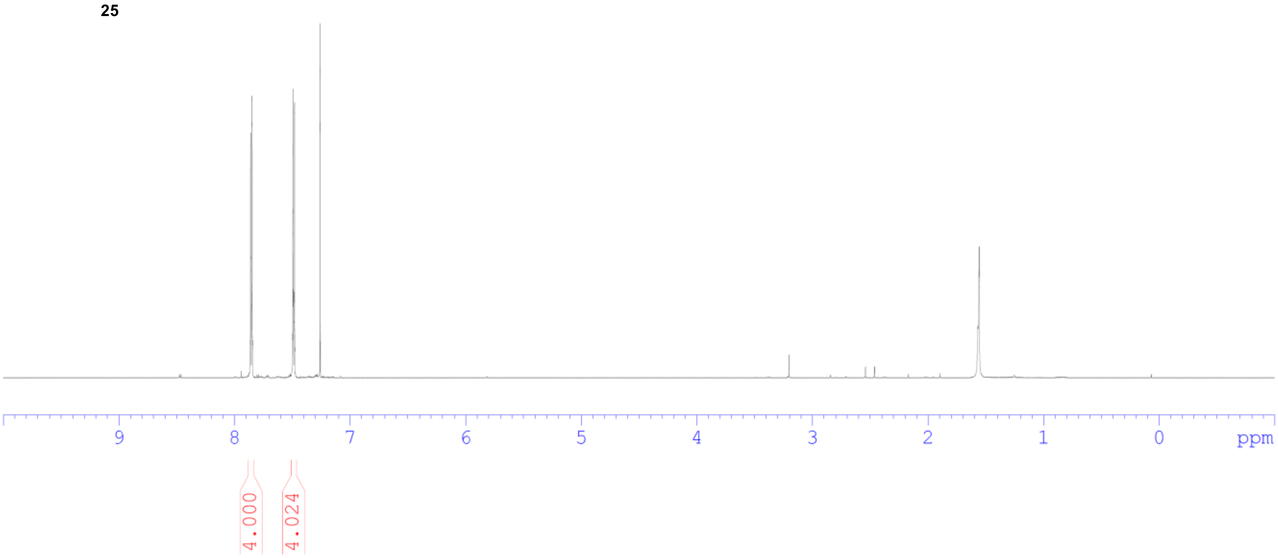

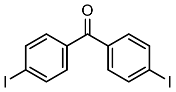

25

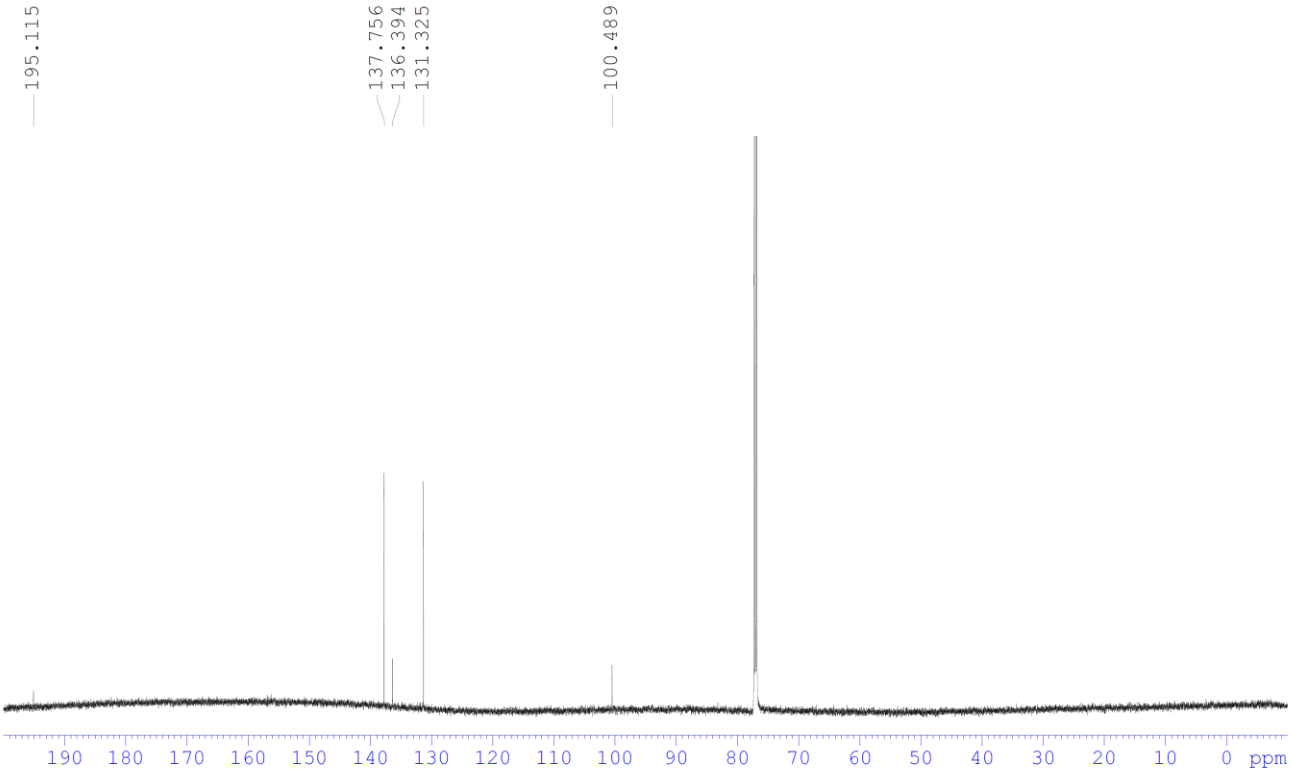

^1H NMR (600 MHz) and ^{13}C NMR (151 MHz) spectra of **27** ($CDCl_3$)

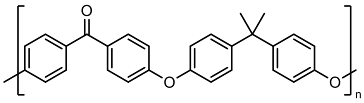

27

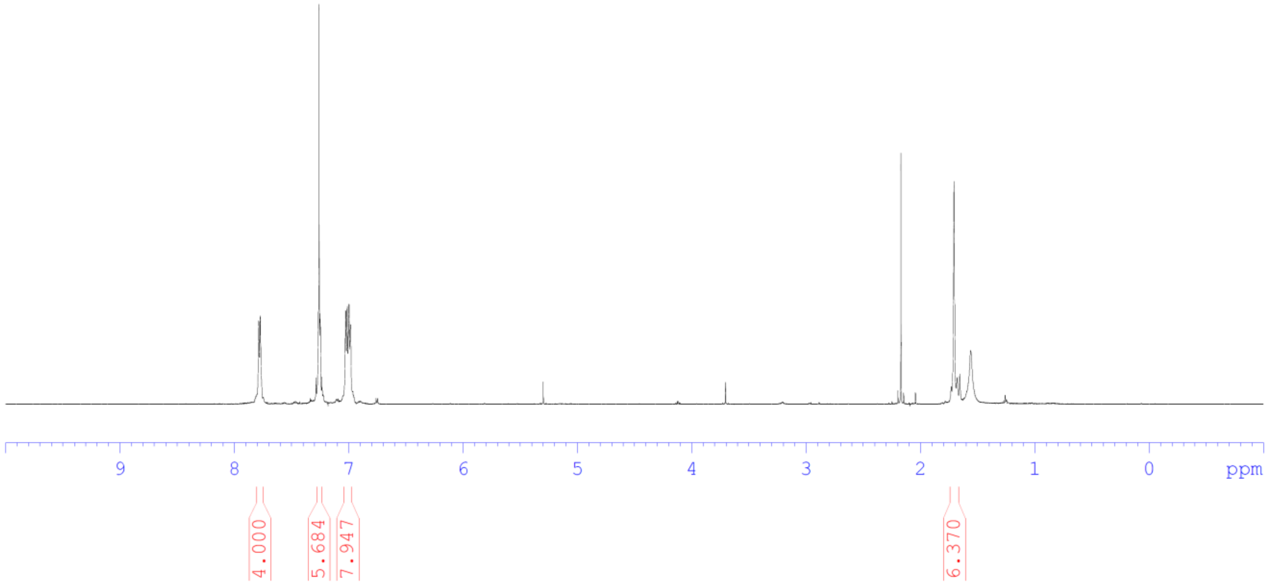

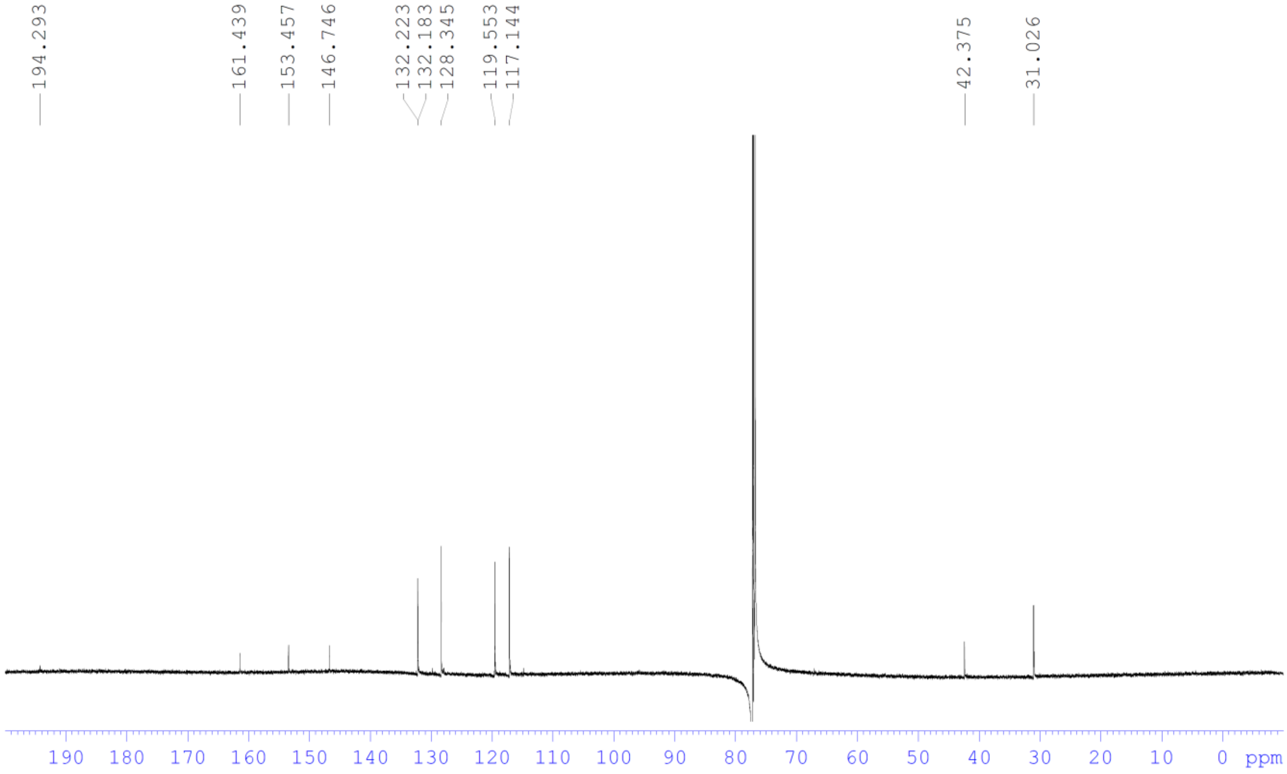

^{1}H NMR (600 MHz) and ^{13}C NMR (151 MHz) spectra of **29** ($CDCl_3$)

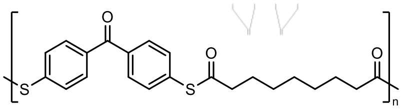

29

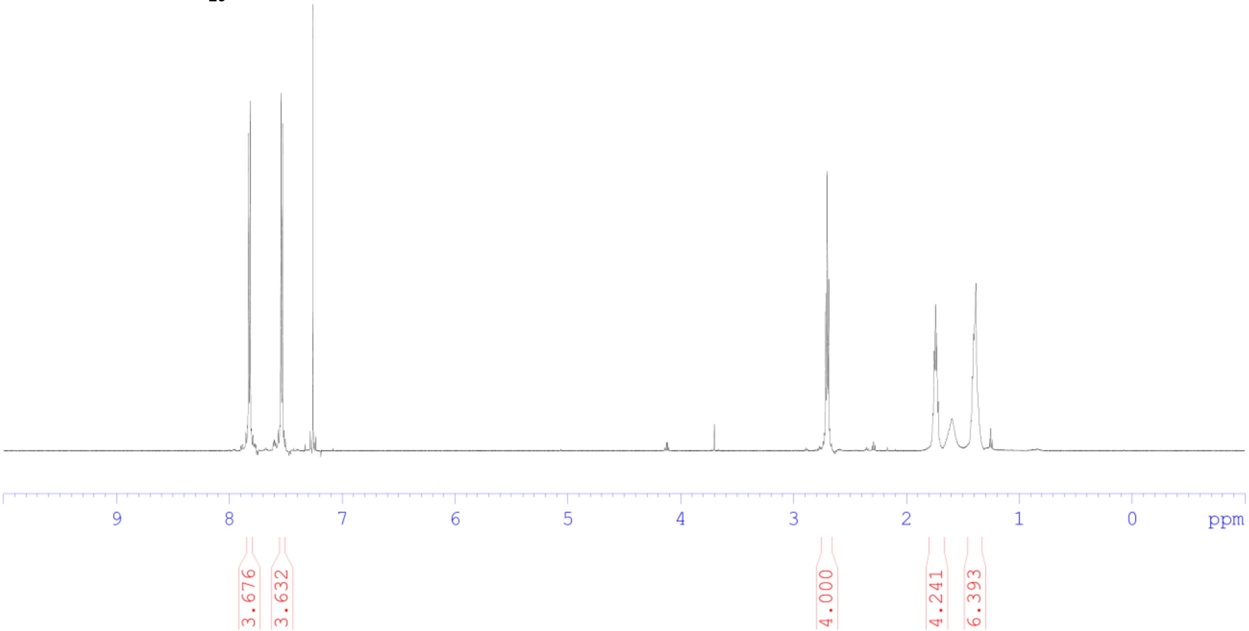

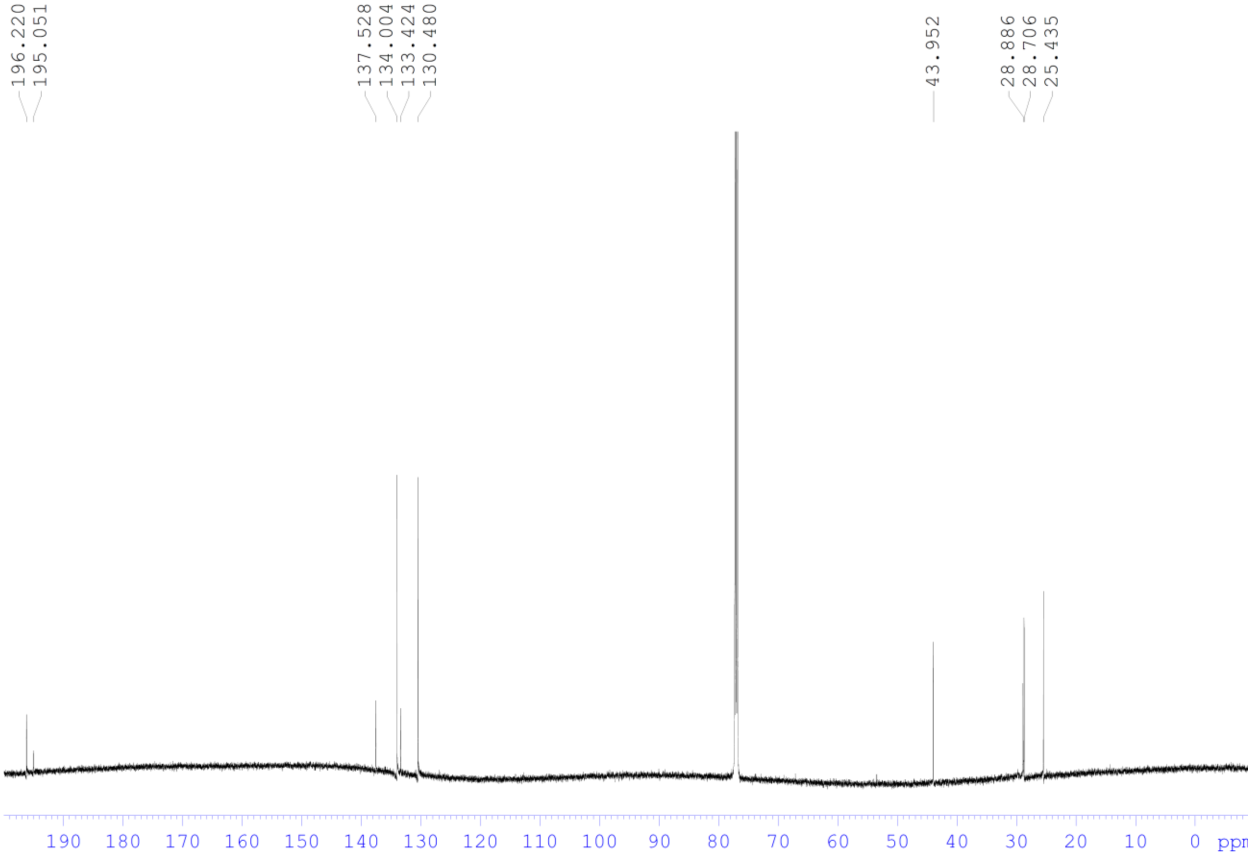

GPC charts.

GPC chart of the polymer **27**.

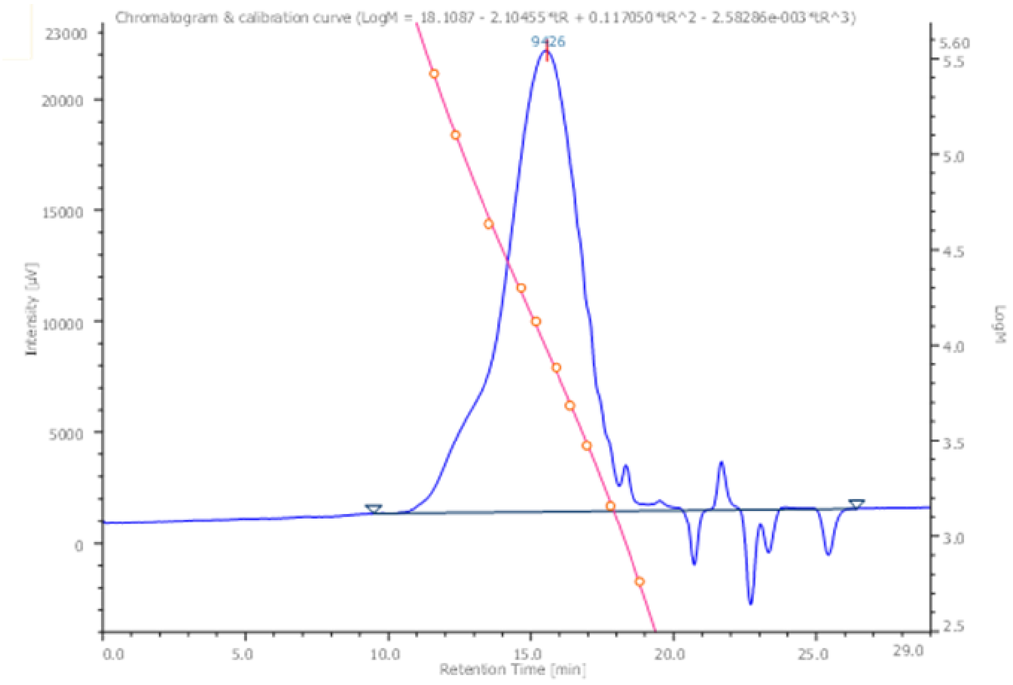

＜Result of Calculation＞ (RI)

tR	15.5500
M_n	6891
M_w	24039
M_z	105197
M_v	24039
M_p	9426
M_z/M_w	4.3761
M_w/M_n	3.4883

GPC chart of the polymer **29**.

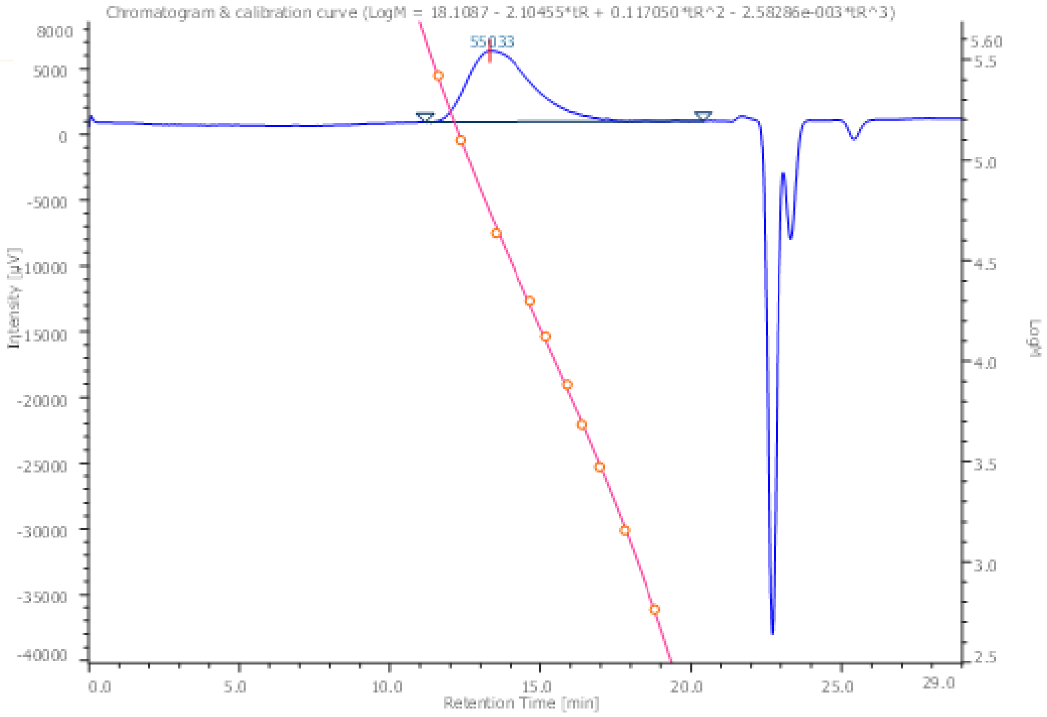

＜Result of Calculation＞ (RI)

tR	13.3167
M_n	24388
M_w	49641
M_z	78330
M_v	49641
M_p	55033
M_z/M_w	1.5779
M_w/M_n	2.0355
