# Supplementary material for: Depolymerization of robust polyetheretherketone to regenerate monomer units using sulfur reagents
Source: Commun Chem. 2023 Jan 24;6:14. doi: 10.1038/s42004-023-00814-8 (PMC9873933; doi:10.1038/s42004-023-00814-8)
Supplement: Supplementary file 5 — Supplementary Data 2 [file 42004_2023_814_MOESM5_ESM.doc]

**Supplementary Data 2**

Computed absolute electronic energies (Hartrees) and relative free Gibbs energies (kcal/mol) for all structures**.**

|  | E (B3LYP) | Correction  to G (B3LYP) | G (B3LYP) |
| --- | --- | --- | --- |
|  | -691.20305434 | 0.183228 | -691.019826 |
| Na_2_S | -722.88388525 | -0.027115 | -722.911001 |
|  | -1136.62319613 | 0.137893 | -1136.485304 |
| MeONa | -277.45762652 | 0.012445 | -277.445182 |
| MeSNa | -600.45416455 | 0.009108 | -600.445057 |
|  | -1014.17871740 | 0.178497 | -1014.000220 |
|  | -1189.23158017 | 0.302239 | -1188.929341 |
|  | -775.52228855 | 0.132323 | -775.389965 |
|  | -1679.88080205 | 0.198574 | -1679.682228 |
|  | -1696.57327319 | 0.122075 | -1696.451198 |
|  | -706.20410462 | 0.048387 | -706.155718 |
|  | -1557.43666892 | 0.239530 | -1557.197139 |
|  | -1451.68518455 | 0.202947 | -1451.482237 |
|  | -1395.68440681 | 0.255384 | -1395.429023 |
|  | -544.46377772 | 0.060716 | -544.403062 |
|  | -1574.12934302 | 0.161354 | -1573.967989 |
|  | -1518.12873355 | 0.215522 | -1517.913212 |
| Me_2_S | -478.02299857 | 0.049099 | -477.973899 |
| MeSH | -438.70661030 | 0.022006 | -438.684604 |

**Optimized Molecular Geometries**

| C | 1.71455 | 2.2034 | 1.21258 |
| --- | --- | --- | --- |
| C | 2.98562 | 2.79351 | 1.20419 |
| C | 3.56611 | 3.18995 | -0.00815 |
| C | 2.87554 | 2.99627 | -1.21211 |
| C | 1.60447 | 2.40615 | -1.20373 |
| C | 1.02397 | 2.00972 | 0.00862 |
| H | 1.27132 | 1.90071 | 2.13823 |
| H | 3.51288 | 2.94139 | 2.12344 |
| H | 4.5366 | 3.64051 | -0.01456 |
| H | 3.31876 | 3.29896 | -2.13777 |
| H | 1.0772 | 2.25828 | -2.12298 |
| C | -1.01071 | 0.92559 | 1.35008 |
| C | -2.28178 | 0.33547 | 1.35847 |
| C | -0.32014 | 1.11928 | 2.55404 |
| C | -2.86228 | -0.06096 | 2.57082 |
| H | -2.80904 | 0.18759 | 0.43922 |
| C | -0.90064 | 0.72285 | 3.76639 |
| H | 0.65035 | 1.56985 | 2.54764 |
| C | -2.17171 | 0.13273 | 3.77478 |
| H | -3.83277 | -0.51153 | 2.57722 |
| H | -0.37337 | 0.87073 | 4.68564 |
| C | -0.3728 | 1.36124 | 0.01783 |
| O | -0.99291 | 1.18732 | -1.06327 |
| C | -3.52598 | 0.81103 | 5.55211 |
| H | -4.29547 | 1.08537 | 4.86108 |
| H | -3.96921 | 0.50835 | 6.47777 |
| H | -2.88338 | 1.6496 | 5.72173 |
| O | -2.76406 | -0.27179 | 5.01187 |

Na_2_S

| S | 0 | 0 | 0.69216 |
| --- | --- | --- | --- |
| Na | 0 | 2.12774 | -0.50339 |
| Na | 0 | -2.12774 | -0.50339 |

| C | 2.66239 | -0.96795 | -0.5626 |
| --- | --- | --- | --- |
| C | 3.80563 | -1.76986 | -0.48413 |
| C | 4.93973 | -1.31675 | 0.19621 |
| C | 4.93286 | -0.04432 | 0.78352 |
| C | 3.79756 | 0.7589 | 0.6949 |
| C | 2.63862 | 0.30255 | 0.03866 |
| H | 1.8116 | -1.33119 | -1.1309 |
| H | 3.80775 | -2.7453 | -0.96256 |
| H | 5.82535 | -1.94287 | 0.2602 |
| H | 5.81332 | 0.3209 | 1.30512 |
| H | 3.7899 | 1.7476 | 1.14365 |
| C | 0.08458 | 0.66903 | -0.18848 |
| C | -0.91787 | 1.46757 | -0.77982 |
| C | -0.29912 | -0.5925 | 0.30744 |
| C | -2.22653 | 1.0191 | -0.9002 |
| H | -0.64872 | 2.45022 | -1.15764 |
| C | -1.61604 | -1.03691 | 0.20283 |
| H | 0.41295 | -1.22957 | 0.82341 |
| C | -2.61822 | -0.25375 | -0.4146 |
| H | -2.96839 | 1.65597 | -1.37414 |
| H | -1.87838 | -2.00965 | 0.60931 |
| C | 1.45537 | 1.22446 | -0.05148 |
| O | 1.63856 | 2.44857 | -0.08028 |
| S | -4.28841 | -0.84324 | -0.59164 |
| Na | -5.72291 | 0.32902 | 0.94982 |

MeONa

| O | 0 | 0.29425 | 0 |
| --- | --- | --- | --- |
| Na | -0.00092 | -1.69255 | 0 |
| C | 0.00095 | 1.65132 | 0 |
| H | -1.01798 | 2.11965 | 0 |
| H | 0.51122 | 2.11826 | 0.88362 |
| H | 0.51122 | 2.11826 | -0.88362 |

MeSNa

| C | -1.56931 | 0.65191 | 0 |
| --- | --- | --- | --- |
| H | -2.54786 | 0.21909 | -0.0002 |
| H | -1.44728 | 1.25737 | 0.87375 |
| H | -1.44704 | 1.2576 | -0.87355 |
| S | -0.34784 | -0.64285 | 0 |
| Na | 1.85668 | 0.33091 | 0 |

| C | 1.76389 | 2.20457 | 1.15206 |
| --- | --- | --- | --- |
| C | 3.03146 | 2.80221 | 1.15334 |
| C | 3.61136 | 3.21792 | -0.05282 |
| C | 2.92368 | 3.03598 | -1.26027 |
| C | 1.65611 | 2.43834 | -1.26155 |
| C | 1.07621 | 2.02263 | -0.05538 |
| H | 1.32113 | 1.88717 | 2.07299 |
| H | 3.55652 | 2.94112 | 2.07525 |
| H | 4.57918 | 3.67423 | -0.05185 |
| H | 3.36645 | 3.35338 | -2.1812 |
| H | 1.13105 | 2.29942 | -2.18346 |
| C | -0.95397 | 0.90905 | 1.26867 |
| C | -2.22154 | 0.3114 | 1.26738 |
| C | -0.26629 | 1.09099 | 2.47611 |
| C | -2.80144 | -0.1043 | 2.47355 |
| H | -2.7466 | 0.17249 | 0.34548 |
| C | -0.84619 | 0.67528 | 3.68227 |
| H | 0.70153 | 1.5473 | 2.47709 |
| C | -2.11376 | 0.07763 | 3.68099 |
| H | -3.76926 | -0.56062 | 2.47257 |
| H | -0.32113 | 0.81419 | 4.60418 |
| C | -0.31672 | 1.36587 | -0.05679 |
| O | -0.93423 | 1.2025 | -1.14102 |
| S | -2.85032 | -0.45038 | 5.21301 |
| C | -3.80764 | 0.88293 | 5.90165 |
| H | -4.57774 | 1.16178 | 5.21311 |
| H | -4.2504 | 0.56553 | 6.82259 |
| H | -3.17024 | 1.72296 | 6.08322 |

| C | -3.28639 | -1.03286 | 0 |
| --- | --- | --- | --- |
| C | -1.89123 | -1.03286 | 0 |
| C | -1.19369 | 0.17489 | 0 |
| C | -1.89134 | 1.3834 | -0.0012 |
| C | -3.28617 | 1.38332 | -0.00168 |
| C | -3.98377 | 0.17511 | -0.00068 |
| H | -3.83614 | -1.98518 | 0.00045 |
| H | -1.34172 | -1.98538 | 0.00132 |
| H | -1.34114 | 2.33554 | -0.00126 |
| H | -3.83629 | 2.3356 | -0.00263 |
| H | -5.08337 | 0.1753 | -0.00086 |
| C | 0.34631 | 0.175 | 0.00089 |
| C | 1.11377 | -1.16015 | 0.00177 |
| C | 0.41382 | -2.36702 | 0.00177 |
| C | 2.50859 | -1.16291 | 0.00247 |
| C | 1.10863 | -3.57635 | 0.00315 |
| H | -0.68583 | -2.3645 | 0.002 |
| C | 3.20376 | -2.37252 | 0.00285 |
| H | 3.06042 | -0.2118 | 0.00229 |
| C | 2.50406 | -3.57914 | 0.00332 |
| H | 0.557 | -4.52766 | 0.00378 |
| H | 4.30352 | -2.37439 | 0.00298 |
| O | 0.97975 | 1.26235 | 0.00089 |
| O | 3.21616 | -4.81923 | 0.00466 |
| C | 3.96014 | -4.94376 | -1.21021 |
| C | 5.29497 | -4.54041 | -1.25515 |
| C | 3.35143 | -5.46855 | -2.35021 |
| C | 6.02065 | -4.66123 | -2.44006 |
| H | 5.77435 | -4.12599 | -0.35643 |
| C | 4.07748 | -5.5904 | -3.53529 |
| H | 2.29944 | -5.78667 | -2.31491 |
| C | 5.41185 | -5.18672 | -3.58043 |
| H | 7.07259 | -4.34275 | -2.47574 |
| H | 3.59739 | -6.00459 | -4.43387 |
| O | 6.15617 | -5.31055 | -4.79515 |
| C | 6.87446 | -6.54706 | -4.79092 |
| C | 8.17538 | -6.59631 | -4.28928 |
| C | 6.2745 | -7.70393 | -5.28816 |
| C | 8.87636 | -7.80206 | -4.28555 |
| H | 8.64837 | -5.68392 | -3.89803 |
| C | 6.9752 | -8.91033 | -5.2836 |
| H | 5.24911 | -7.66533 | -5.68341 |
| C | 8.27599 | -8.95955 | -4.78253 |
| H | 9.902 | -7.84082 | -3.89074 |
| H | 6.50176 | -9.8224 | -5.67534 |
| H | 8.82887 | -9.91013 | -4.77943 |

| C | 1.66322 | 0.2686 | 0 |
| --- | --- | --- | --- |
| C | 3.05838 | 0.2686 | 0 |
| C | 3.75592 | 1.47635 | 0 |
| C | 3.05827 | 2.68486 | -0.0012 |
| C | 1.66344 | 2.68478 | -0.00168 |
| C | 0.96584 | 1.47657 | -0.00068 |
| H | 1.11346 | -0.68372 | 0.00045 |
| H | 3.60789 | -0.68392 | 0.00132 |
| H | 4.8556 | 1.47643 | 0.00063 |
| H | 3.60847 | 3.637 | -0.00126 |
| H | 1.11332 | 3.63706 | -0.00263 |
| O | -0.46416 | 1.47681 | -0.00092 |
| C | -0.94217 | 1.47412 | 1.34682 |
| C | -1.17714 | 0.26453 | 2.00117 |
| C | -1.17342 | 2.6807 | 2.00729 |
| C | -1.64389 | 0.26163 | 3.31545 |
| H | -0.99546 | -0.68654 | 1.47993 |
| C | -1.63938 | 2.67797 | 3.32231 |
| H | -0.98811 | 3.63414 | 1.49178 |
| C | -1.87476 | 1.46872 | 3.9764 |
| H | -1.82972 | -0.69174 | 3.83107 |
| H | -1.82123 | 3.62947 | 3.84295 |
| O | -2.35325 | 1.46542 | 5.32397 |
| Na | -4.41325 | 1.47121 | 5.32223 |

| C | 1.13705 | 0.85656 | 0.14374 |
| --- | --- | --- | --- |
| C | -0.25046 | 0.86083 | 0.28894 |
| C | -1.00759 | 2.0221 | 0.06076 |
| C | -0.32501 | 3.19817 | -0.31125 |
| C | 1.05061 | 3.20393 | -0.48092 |
| C | 1.7918 | 2.03023 | -0.2541 |
| H | 1.7084 | -0.04383 | 0.33995 |
| H | -0.74084 | -0.05132 | 0.61583 |
| H | -0.90105 | 4.10532 | -0.46695 |
| H | 1.57934 | 4.10306 | -0.78372 |
| C | -2.48139 | 2.09348 | 0.26944 |
| C | -3.33203 | 0.8671 | 0.11488 |
| C | -3.0404 | -0.16622 | -0.79292 |
| C | -4.52351 | 0.78549 | 0.85068 |
| C | -3.90662 | -1.24391 | -0.95122 |
| H | -2.14533 | -0.11483 | -1.40526 |
| C | -5.38529 | -0.30127 | 0.71672 |
| H | -4.76819 | 1.59288 | 1.53458 |
| C | -5.08577 | -1.33135 | -0.18991 |
| H | -3.67109 | -2.02147 | -1.67396 |
| H | -6.28772 | -0.33364 | 1.31782 |
| O | -3.01767 | 3.1625 | 0.56692 |
| O | 3.13925 | 2.13134 | -0.44066 |
| C | 3.95843 | 1.00477 | -0.24753 |
| C | 4.24419 | 0.16268 | -1.32189 |
| C | 4.55156 | 0.78415 | 0.99518 |
| C | 5.11664 | -0.91163 | -1.14923 |
| H | 3.7863 | 0.35575 | -2.28928 |
| C | 5.42421 | -0.29018 | 1.1675 |
| H | 4.33133 | 1.45786 | 1.81998 |
| H | 5.34424 | -1.56973 | -1.98508 |
| H | 5.89059 | -0.46488 | 2.13462 |
| S | -6.10068 | -2.76771 | -0.4574 |
| C | 5.73766 | -1.17598 | 0.10141 |
| O | 6.56373 | -2.18442 | 0.26221 |
| Na | 7.89667 | -3.64454 | 0.44985 |
| Na | -8.02238 | -2.38466 | 0.94563 |

| S | -6.10068 | -2.76771 | -0.4574 |
| --- | --- | --- | --- |
| Na | -8.02029 | -2.38678 | 0.94905 |
| C | -5.08519 | -1.33051 | -0.18976 |
| C | -4.13002 | -1.3309 | 0.82717 |
| C | -5.24423 | -0.20398 | -0.99671 |
| C | -3.33459 | -0.20471 | 1.03745 |
| H | -4.00541 | -2.21891 | 1.46368 |
| C | -4.44796 | 0.92227 | -0.78713 |
| H | -5.99688 | -0.20358 | -1.79837 |
| C | -3.49333 | 0.92213 | 0.22983 |
| H | -2.58213 | -0.20471 | 1.83939 |
| H | -4.5733 | 1.81019 | -1.4238 |
| C | -2.61483 | 2.16544 | 0.46223 |
| C | -1.56231 | 2.16208 | 1.58642 |
| C | -1.40598 | 1.03392 | 2.39221 |
| C | -0.76522 | 3.2868 | 1.79901 |
| C | -0.45322 | 1.03083 | 3.41076 |
| H | -2.03507 | 0.14765 | 2.22485 |
| C | 0.18856 | 3.28351 | 2.8172 |
| H | -0.88817 | 4.17599 | 1.1639 |
| C | 0.34458 | 2.15583 | 3.62314 |
| H | -0.33037 | 0.14187 | 4.04634 |
| H | 0.81717 | 4.1703 | 2.98431 |
| O | -2.75407 | 3.18414 | -0.26333 |
| S | 1.56049 | 2.15147 | 4.92311 |
| Na | 3.59139 | 3.17722 | 4.1285 |

| C | 1.36742 | 0.28356 | 0.53462 |
| --- | --- | --- | --- |
| C | 0.66098 | -0.91753 | 0.2552 |
| C | -0.66027 | -0.91832 | -0.25475 |
| C | -1.36762 | 0.28192 | -0.53536 |
| C | -0.64681 | 1.48785 | -0.25304 |
| C | 0.6456 | 1.48863 | 0.25136 |
| H | 1.1298 | -1.86845 | 0.52886 |
| H | -1.12849 | -1.86987 | -0.52722 |
| H | -1.15479 | 2.42905 | -0.45873 |
| H | 1.1528 | 2.43044 | 0.45619 |
| O | -2.62555 | 0.29231 | -0.95274 |
| O | 2.62541 | 0.29527 | 0.9517 |
| Na | -3.19089 | -0.7291 | 0.78444 |
| Na | 3.19143 | -0.73076 | -0.78253 |

| C | 1.13705 | -0.85656 | -0.14374 |
| --- | --- | --- | --- |
| C | -0.25046 | -0.86083 | -0.28894 |
| C | -1.00759 | -2.0221 | -0.06076 |
| C | -0.32501 | -3.19817 | 0.31125 |
| C | 1.05061 | -3.20393 | 0.48092 |
| C | 1.7918 | -2.03023 | 0.2541 |
| H | 1.7084 | 0.04383 | -0.33995 |
| H | -0.74084 | 0.05132 | -0.61583 |
| H | -0.90105 | -4.10532 | 0.46695 |
| H | 1.57934 | -4.10306 | 0.78372 |
| C | -2.48139 | -2.09348 | -0.26944 |
| C | -3.33203 | -0.8671 | -0.11488 |
| C | -3.0404 | 0.16622 | 0.79292 |
| C | -4.52351 | -0.78549 | -0.85068 |
| C | -3.90662 | 1.24391 | 0.95122 |
| H | -2.14533 | 0.11483 | 1.40526 |
| C | -5.38529 | 0.30127 | -0.71672 |
| H | -4.76819 | -1.59288 | -1.53458 |
| C | -5.08577 | 1.33135 | 0.18991 |
| H | -3.67109 | 2.02147 | 1.67396 |
| H | -6.28772 | 0.33364 | -1.31782 |
| O | -3.01767 | -3.1625 | -0.56692 |
| O | 3.13925 | -2.13134 | 0.44066 |
| C | 3.95843 | -1.00477 | 0.24753 |
| C | 4.24419 | -0.16268 | 1.32189 |
| C | 4.55156 | -0.78415 | -0.99518 |
| C | 5.11664 | 0.91163 | 1.14923 |
| H | 3.7863 | -0.35575 | 2.28928 |
| C | 5.42421 | 0.29018 | -1.1675 |
| H | 4.33133 | -1.45786 | -1.81998 |
| H | 5.34424 | 1.56973 | 1.98508 |
| H | 5.89059 | 0.46488 | -2.13462 |
| S | -6.10068 | 2.76771 | 0.4574 |
| C | -7.55407 | 2.47801 | -0.60372 |
| H | -8.08612 | 1.56625 | -0.31733 |
| H | -8.21142 | 3.33618 | -0.43952 |
| H | -7.28342 | 2.44049 | -1.66284 |
| C | 5.73766 | 1.17598 | -0.10141 |
| O | 6.56373 | 2.18442 | -0.26221 |
| Na | 7.89667 | 3.64454 | -0.44985 |

| C | -3.86973 | -0.60702 | -0.12384 |
| --- | --- | --- | --- |
| C | -2.75091 | -1.2063 | 0.50017 |
| C | -3.65893 | 0.64941 | -0.74497 |
| C | -1.50016 | -0.59296 | 0.50211 |
| H | -2.87267 | -2.16527 | 0.99747 |
| C | -2.41734 | 1.27371 | -0.71721 |
| H | -4.48457 | 1.13623 | -1.26045 |
| C | -1.30442 | 0.66361 | -0.1043 |
| H | -0.67737 | -1.08586 | 1.01206 |
| H | -2.29269 | 2.24376 | -1.19102 |
| C | -0.01208 | 1.40274 | -0.05637 |
| C | 1.28127 | 0.66789 | 0.0132 |
| C | 1.48225 | -0.6025 | -0.56211 |
| C | 2.3898 | 1.29336 | 0.61851 |
| C | 2.73347 | -1.21396 | -0.53763 |
| H | 0.66329 | -1.10812 | -1.06583 |
| C | 3.63139 | 0.67091 | 0.66986 |
| H | 2.26141 | 2.27461 | 1.06772 |
| C | 3.84748 | -0.60038 | 0.08186 |
| H | 2.85929 | -2.18424 | -1.01148 |
| H | 4.45132 | 1.17165 | 1.18104 |
| O | -0.01348 | 2.6443 | -0.07307 |
| S | -5.45313 | -1.41999 | -0.14206 |
| S | 5.42831 | -1.41722 | 0.12793 |
| C | -6.62979 | -0.44978 | 0.77584 |
| H | -6.8495 | 0.44806 | 0.23685 |
| H | -7.52899 | -1.01377 | 0.91095 |
| H | -6.2182 | -0.2004 | 1.73151 |
| C | 6.61979 | -0.45744 | -0.7818 |
| H | 6.83563 | 0.44352 | -0.24645 |
| H | 7.51874 | -1.02531 | -0.90141 |
| H | 6.22122 | -0.21357 | -1.74439 |

| C | 1.13705 | -0.85656 | -0.14374 |
| --- | --- | --- | --- |
| C | -0.25046 | -0.86083 | -0.28894 |
| C | -1.00759 | -2.0221 | -0.06076 |
| C | -0.32501 | -3.19817 | 0.31125 |
| C | 1.05061 | -3.20393 | 0.48092 |
| C | 1.7918 | -2.03023 | 0.2541 |
| H | 1.7084 | 0.04383 | -0.33995 |
| H | -0.74084 | 0.05132 | -0.61583 |
| H | -0.90105 | -4.10532 | 0.46695 |
| H | 1.57934 | -4.10306 | 0.78372 |
| C | -2.48139 | -2.09348 | -0.26944 |
| C | -3.33203 | -0.8671 | -0.11488 |
| C | -3.0404 | 0.16622 | 0.79292 |
| C | -4.52351 | -0.78549 | -0.85068 |
| C | -3.90662 | 1.24391 | 0.95122 |
| H | -2.14533 | 0.11483 | 1.40526 |
| C | -5.38529 | 0.30127 | -0.71672 |
| H | -4.76819 | -1.59288 | -1.53458 |
| C | -5.08577 | 1.33135 | 0.18991 |
| H | -3.67109 | 2.02147 | 1.67396 |
| H | -6.28772 | 0.33364 | -1.31782 |
| O | -3.01767 | -3.1625 | -0.56692 |
| O | 3.13925 | -2.13134 | 0.44066 |
| C | 3.95843 | -1.00477 | 0.24753 |
| C | 4.24419 | -0.16268 | 1.32189 |
| C | 4.55156 | -0.78415 | -0.99518 |
| C | 5.11664 | 0.91163 | 1.14923 |
| H | 3.7863 | -0.35575 | 2.28928 |
| C | 5.42421 | 0.29018 | -1.1675 |
| H | 4.33133 | -1.45786 | -1.81998 |
| H | 5.34424 | 1.56973 | 1.98508 |
| H | 5.89059 | 0.46488 | -2.13462 |
| S | -6.10068 | 2.76771 | 0.4574 |
| C | -7.55407 | 2.47801 | -0.60372 |
| H | -8.08612 | 1.56625 | -0.31733 |
| H | -8.21142 | 3.33618 | -0.43952 |
| H | -7.28342 | 2.44049 | -1.66284 |
| C | 5.73766 | 1.17598 | -0.10141 |
| O | 6.55608 | 2.17508 | -0.26072 |
| H | 7.45714 | 1.87755 | -0.11521 |

| C | 0.06132 | 2.13839 | 0 |
| --- | --- | --- | --- |
| C | -1.1583 | 1.45295 | 0 |
| C | -1.18764 | 0.05851 | 0 |
| C | 0 | -0.7301 | 0 |
| C | 1.21991 | 0.0039 | 0 |
| C | 1.24932 | 1.40064 | 0 |
| H | -2.08465 | 2.02398 | 0 |
| H | -2.1452 | -0.46009 | 0 |
| H | 2.15511 | -0.55369 | 0 |
| H | 2.20688 | 1.92213 | 0 |
| O | -0.02958 | -2.03457 | 0 |
| Na | -0.20292 | -4.05514 | 0 |
| O | 0.03451 | 3.52068 | 0 |
| H | 0.95294 | 3.83955 | 0 |

| C | -2.6707 | -1.18677 | -0.12918 |
| --- | --- | --- | --- |
| C | -1.45415 | -0.495 | -0.05573 |
| C | -1.44428 | 0.90635 | -0.04842 |
| C | -2.65094 | 1.61592 | -0.11457 |
| C | -3.86749 | 0.92415 | -0.18802 |
| C | -3.87737 | -0.47719 | -0.19532 |
| H | -2.67824 | -2.25673 | -0.13475 |
| H | -0.53284 | -1.03678 | -0.00522 |
| H | -2.6434 | 2.68588 | -0.109 |
| H | -4.78881 | 1.46593 | -0.23853 |
| C | -0.10741 | 1.66653 | 0.03229 |
| C | 1.21859 | 0.88678 | 0.10497 |
| C | 1.20872 | -0.51457 | 0.09767 |
| C | 2.43514 | 1.57855 | 0.17842 |
| C | 2.41539 | -1.22415 | 0.16381 |
| H | 0.27986 | -1.04275 | 0.04159 |
| C | 3.64181 | 0.86897 | 0.24456 |
| H | 2.44268 | 2.64851 | 0.184 |
| C | 3.63193 | -0.53238 | 0.23726 |
| H | 2.40784 | -2.2941 | 0.15823 |
| H | 4.57067 | 1.39715 | 0.30064 |
| O | -0.09854 | 2.92488 | 0.03885 |
| S | 5.16459 | -1.43365 | 0.32126 |
| S | -5.42257 | -1.35585 | -0.28862 |
| C | -6.70252 | -0.38883 | 0.48275 |
| H | -6.79463 | 0.54943 | -0.02329 |
| H | -7.63138 | -0.91701 | 0.42667 |
| H | -6.45095 | -0.21761 | 1.50857 |
| Na | 6.9159 | -0.13811 | -0.70955 |

| C | 1.73051 | 0.50775 | 0.39313 |
| --- | --- | --- | --- |
| C | 0.34178 | 0.65384 | 0.34424 |
| C | -0.25264 | 1.8606 | -0.06569 |
| C | 0.59235 | 2.93109 | -0.42206 |
| C | 1.97296 | 2.80316 | -0.38042 |
| C | 2.54239 | 1.58574 | 0.02772 |
| H | 2.15944 | -0.4351 | 0.71886 |
| H | -0.27448 | -0.17974 | 0.67047 |
| H | 0.15007 | 3.86983 | -0.74555 |
| H | 2.61664 | 3.61746 | -0.6983 |
| C | -1.71859 | 2.10575 | -0.03319 |
| C | -2.68148 | 0.96369 | -0.08774 |
| C | -2.41806 | -0.24382 | -0.77103 |
| C | -3.9572 | 1.12586 | 0.48213 |
| C | -3.38617 | -1.23919 | -0.86094 |
| H | -1.45947 | -0.4158 | -1.24505 |
| C | -4.90111 | 0.10925 | 0.43524 |
| H | -4.18801 | 2.03898 | 1.02483 |
| C | -4.64722 | -1.10575 | -0.23476 |
| H | -3.15479 | -2.15791 | -1.39562 |
| H | -5.84383 | 0.29707 | 0.94916 |
| O | -2.14804 | 3.2925 | 0.0152 |
| O | 3.90898 | 1.53037 | -0.03451 |
| C | 4.56109 | 0.30259 | 0.05005 |
| C | 4.68826 | -0.45076 | -1.12168 |
| C | 5.14275 | -0.11334 | 1.25361 |
| C | 5.48166 | -1.6032 | -1.10004 |
| H | 4.2214 | -0.10881 | -2.04093 |
| C | 5.86188 | -1.3173 | 1.26757 |
| H | 5.00518 | 0.46005 | 2.16881 |
| H | 5.67029 | -2.16577 | -2.01063 |
| H | 6.31482 | -1.67477 | 2.18925 |
| S | -5.7978 | -2.4378 | -0.30839 |
| C | 6.0645 | -2.04372 | 0.0908 |
| O | 6.85215 | -3.17237 | 0.02443 |
| H | 7.06933 | -3.54948 | 0.8992 |
| Na | -7.68477 | -1.3444 | 0.80065 |

Me_2_S

| S | 0 | 0 | 0.66411 |
| --- | --- | --- | --- |
| C | 0 | 1.39417 | -0.51554 |
| H | -0.89508 | 1.38144 | -1.14669 |
| H | 0 | 2.31572 | 0.07375 |
| H | 0.89507 | 1.38144 | -1.14669 |
| C | 0 | -1.39417 | -0.51554 |
| H | 0 | -2.31572 | 0.07375 |
| H | -0.89507 | -1.38144 | -1.14669 |
| H | 0.89508 | -1.38144 | -1.14669 |

MeSH

| C | -1.56924 | 0.65186 | -0.00001 |
| --- | --- | --- | --- |
| H | -2.54784 | 0.21914 | -0.00016 |
| H | -1.44711 | 1.2573 | 0.87373 |
| H | -1.44696 | 1.25754 | -0.87357 |
| S | -0.34791 | -0.64302 | -0.00008 |
| H | 0.85019 | -0.11324 | 0.00009 |
